# Towards a Natural Classification of *Hyphodontia* Sensu Lato and the Trait Evolution of Basidiocarps within *Hymenochaetales* (*Basidiomycota*)

**DOI:** 10.3390/jof7060478

**Published:** 2021-06-12

**Authors:** Xue-Wei Wang, Tom W. May, Shi-Liang Liu, Li-Wei Zhou

**Affiliations:** 1 State Key Laboratory of Mycology, Institute of Microbiology, Chinese Academy of Sciences, Beijing 100101, China; xuewei_wang1995@im.ac.cn (X.-W.W.); liushiliang@im.ac.cn (S.-L.L.); 2 Institute of Applied Ecology, Chinese Academy of Sciences, Shenyang 110016, China; 3 University of Chinese Academy of Sciences, Beijing 100049, China; 4 Royal Botanic Gardens Victoria, Birdwood Avenue, Melbourne 3004, Australia; tom.may@rbg.vic.gov.au

**Keywords:** *Chaetoporellaceae*, corticioid fungi, *Hyphodontiaceae*, molecular clock, *Schizoporaceae*, wood-inhabiting fungi, 17 new taxa

## Abstract

*Hyphodontia* sensu lato, belonging to *Hymenochaetales*, accommodates corticioid wood-inhabiting basidiomycetous fungi with resupinate basidiocarps and diverse hymenophoral characters. Species diversity of *Hyphodontia* sensu lato has been extensively explored worldwide, but in previous studies the six accepted genera in *Hyphodontia* sensu lato, viz. *Fasciodontia*, *Hastodontia*, *Hyphodontia*, *Kneiffiella*, *Lyomyces* and *Xylodon* were not all strongly supported from a phylogenetic perspective. Moreover, the relationships among these six genera in *Hyphodontia* sensu lato and other lineages within *Hymenochaetales* are not clear. In this study, we performed comprehensive phylogenetic analyses on the basis of multiple loci. For the first time, the independence of each of the six genera receives strong phylogenetic support. The six genera are separated in four clades within *Hymenochaetales*: *Fasciodontia*, *Lyomyces* and *Xylodon* are accepted as members of a previously known family *Schizoporaceae*, *Kneiffiella* and *Hyphodontia* are, respectively, placed in two monotypic families, viz. a previous name *Chaetoporellaceae* and a newly introduced name *Hyphodontiaceae*, and *Hastodontia* is considered to be a genus with an uncertain taxonomic position at the family rank within *Hymenochaetales*. The three families emerged between 61.51 and 195.87 million years ago. Compared to other families in the *Hymenochaetales*, these ages are more or less similar to those of *Coltriciaceae*, *Hymenochaetaceae* and *Oxyporaceae*, but much older than those of the two families *Neoantrodiellaceae* and *Nigrofomitaceae*. In regard to species, two, one, three and 10 species are newly described from *Hyphodontia*, *Kneiffiella*, *Lyomyces* and *Xylodon*, respectively. The taxonomic status of additional 30 species names from these four genera is briefly discussed; an epitype is designated for *X. australis*. The resupinate habit and poroid hymenophoral configuration were evaluated as the ancestral state of basidiocarps within *Hymenochaetales*. The resupinate habit mainly remains, while the hymenophoral configuration mainly evolves to the grandinioid-odontioid state and also back to the poroid state at the family level. Generally, a taxonomic framework for *Hymenochaetales* with an emphasis on members belonging to *Hyphodontia* sensu lato is constructed, and trait evolution of basidiocarps within *Hymenochaetales* is revealed accordingly.

## 1. Introduction

*Hyphodontia* was erected with *Gonatobotrys pallidulus* as the generic type in 1958 [1] and was put in the family *Chaetoporellaceae* that is typified by *Chaetoporellus* [2]. Since its erection, the genus name *Hyphodontia* has been accepted worldwide and widely used in many papers. For this reason, *Chaetoporellus*, *Grandinia*, *Kneiffiella*, *Lyomyces*, *Schizopora* and *Xylodon*, once considered to be former synonyms of *Hyphodontia*, were all rejected against a conserved *Hyphodontia* for a stable nomenclature [3,4,5]. When molecular phylogeny indicated that the enlarged concept of *Hyphodontia* was polyphyletic [6,7,8], these generic names except *Grandinia* were reintroduced for species of *Hyphodontia* [9,10]. Moreover, additional genera, such as *Alutaceodontia*, *Basidioradulum*, *Deviodontia*, *Fasciodontia*, *Hastodontia*, *Lagarobasidium*, *Palifer* and *Rogersella*, have also been used to accommodate certain species formerly placed in *Hyphodontia* [2,10,11,12,13]. Therefore, many species of these genera sharing similar morphological characters are normally placed in a widely delimited ‘*Hyphodontia* sensu lato’.

*Hyphodontia* sensu lato includes corticioid, wood-inhabiting, basidiomycetous fungi possessing resupinate basidiomes with diverse hymenophoral and cystidial morphology [14,15,16,17,18]. The members of *Hyphodontia* sensu lato are widely distributed in various forest ecosystems from boreal, temperate, subtropical to tropical zones and cause a white rot of woody substrates [19].

Species diversity of *Hyphodontia* sensu lato has been systematically surveyed in Europe for the last several decades [19,20,21,22]. These researches on European species laid the groundwork for treatments of species from North America [23,24], South America [25,26,27], Africa [9,28], Central Asia [29] and East Asia [30,31]. With reference to these works, many new species of *Hyphodontia* sensu lato were introduced worldwide in scattered publications [32,33,34]. Recently, Riebesehl & Langer [16] summarized the information on all 120 accepted species of *Hyphodontia* sensu lato and made combinations according to the combined evidence of morphology and phylogeny, which provided a foundation for further species identification of this fungal group. After this summary work, 32 species of *Hyphodontia* sensu lato have been newly described [13,15,16,17,18,35,36,37,38,39,40,41,42,43], which reflects the taxonomic study on *Hyphodontia* sensu lato as a hot topic. Moreover, *Deviodontia pilaecystidiata* was included in *Kneiffiella* [13], while excluding *Hyphodontia bubalina*, *H. chinensis* and *H. mongolica*, three later synonyms [13,18], and *Lagarobasidium calongei*, which possibly belongs in *Polyporales* [40]. Eventually, the number of species in *Hyphodontia* sensu lato is 149 before the current study.

At the generic level, after a series of phylogenetic studies, six genera, viz. *Fasciodontia*, *Hastodontia*, *Hyphodontia*, *Lyomyces*, *Kneiffiella* and *Xylodon* were eventually accepted to accommodate the members of *Hyphodontia* sensu lato [13,16,17,18,35,40,44]. However, their generic delimitations did not all receive strong support from phylogenies based on ITS and/or nLSU [16,17,18,40,44].

Besides *Chaetoporellaceae* being typified by *Chaetoporellus* and also including *Hyphodontia*, another family *Schizoporaceae* was also erected by Walter Jülich with *Schizopora* as the type genus [2]. Except for the type genus *Schizopora*, all the other three genera *Fibriciellum*, *Fibricium* and *Fibrodontia* originally exemplified in *Schizoporaceae* [2] have been considered to be outside this family [8]. Although these two family names are both legitimate in the order *Hymenochaetales* [45,46], they are not mentioned to accommodate any genera of *Hyphodontia* sensu lato in recent phylogenetic analyses. Indeed, almost all taxonomic studies did not try to address the taxonomic status of species of *Hyphodontia* sensu lato at the family level, including “Die Gattung *Hyphodontia* John Eriksson” the most important monograph to date [19] and “A key to the species of *Hyphodontia* sensu lato” the first comprehensive key to all genera and species [14].

The aim of the current study is to further explore the species diversity of *Hyphodontia* sensu lato in the Asia-Pacific region, and more importantly, to construct a more natural taxonomic system of *Hyphodontia* sensu lato within the *Hymenochaetales*, based on multilocus phylogenetic analyses. In addition, the trait evolution of basidiocarps within *Hymenochaetales* is explored.

## 2. Materials and Methods

### 2.1. Voucher Deposition

Specimens studied are deposited at the Fungarium, Institute of Microbiology, Chinese Academy of Sciences (HMAS), Beijing, China, the herbarium of the Institute of Applied Ecology, Chinese Academy of Sciences (IFP), Shenyang, China, Institute of Microbiology, Beijing Forestry University (BJFC), Beijing, China, the National Herbarium of Victoria (MEL), Melbourne, Australia, and Josef Vlasák’s private herbarium (JV), České Budějovice, Czech Republic. Regarding other herbarium codes mentioned in this study, their full names can be found in Index Herbariorum (http://sweetgum.nybg.org/science/ih/, accessed on 18 May 2021).

### 2.2. Morphological Examination

Morphological observations were made with Nikon SMZ 645 and SMZ 1000 stereomicroscopes (Tokyo, Japan), and a Nikon Eclipse 80i light microscope (Tokyo, Japan) at magnifications up to 1000×. The microscopic procedure followed Wang et al. [44]. Specimen sections were mounted in Cotton Blue (CB), Melzer’s reagent (IKI) and 5% potassium hydroxide (KOH). All measurements were made from materials in CB. When presenting the variation of basidiospore sizes, 5% of the measurements were excluded from each end of the range and are given in parentheses. Special color terms follow Petersen [47]. Drawings were made with the aid of a drawing tube. The following abbreviations are used in the text: L = mean basidiospore length (arithmetic average of all measured basidiospores), W = mean basidiospore width (arithmetic average of all measured basidiospores), Q = variation in the L/W ratios between the specimens studied, and (a/b) = number of basidiospores (a) measured from a given number (b) of specimens.

### 2.3. Molecular Sequencing

Crude DNA was extracted as templates for subsequent PCR amplifications from basidiocarps of dry specimens using CTAB rapid plant genome extraction kit-DN14 (Aidlab Biotechnologies Co. Ltd., Beijing, China). The primer pairs ITS1F/ITS4 and ITS5/ITS4 [48,49], LR0R/LR7 [50], MS1/MS2 [48], EF1-526F/EF1-1567R and EF1-983F/EF1-1953R [51,52], RPB1-Af/RPB1-Cr [53], fRPB2-5F/fRPB2-7cR [54] and bRPB2-6F/bRPB2-7.1R [55], and ATP6-1/ATP6-2 and ATP6-3/ATP6-4 [56] were selected for amplifying ITS, nLSU, mt-SSU, *tef1α*, *rpb1*, *rpb2* and *atp6* regions, respectively. The PCR procedures were as follows: for ITS region, initial denaturation at 95 °C for 3 min, followed by 34 cycles at 94 °C for 40 s, 57.2 °C for 45 s and 72 °C for 1 min, and a final extension at 72 °C for 10 min; for nLSU region: initial denaturation at 94 °C for 1 min, followed by 34 cycles at 94 °C for 30 s, 47.2 °C for 1 min and 72 °C for 1.5 min, and a final extension at 72 °C for 10 min; for mt-SSU region, initial denaturation at 94 °C for 3 min, followed by 34 cycles at 94 °C for 40 s, 55 °C for 45 s and 72 °C for 1 min, and a final extension at 72 °C for 10 min; for *tef1α*, *rpb1* and *rpb2* regions, initial denaturation at 94 °C for 2 min, followed by 9 cycles at 94 °C for 40 s, 60 °C for 40 s and 72°C for 2 min and 36 cycles at 94°C for 45 s, 55 °C for 1.5 min and 72 °C for 2 min, and a final extension at 72 °C for 10 min; for *apt6* region, initial denaturation at 94 °C for 2 min, followed by 4 cycles at 94 °C for 35 s, 37 °C for 55 s and 72 °C for 1 min and 29 cycles at 94 °C for 35 s, 45 °C for 55 s and 72 °C for 1 min, and a final extension at 72 °C for 10 min. The PCR products were sequenced with the same primers in PCR amplifications at the Beijing Genomics Institute, Beijing, China. All newly generated sequences were deposited in GenBank (https://www.ncbi.nlm.nih.gov/genbank/; Table S1).

### 2.4. Phylogenetic Analyses

Besides the newly generated sequences, additional reliable sequences downloaded from GenBank (Tables S1 and S2) were also incorporated in the datasets for phylogenetic analyses. Seven datasets were employed. (1) The ITS dataset was used to differentiate the species identities of studied specimens belonging to *Hyphodontia* sensu lato. All vouchers belonging to *Hyphodontia* sensu lato as listed in Table S1 were included and *Auricularia cornea* was included as an outgroup taxon. (2) The combined dataset of ITS, nLSU and mt-SSU regions was used to explore the phylogenetic relationships of members belonging to *Hyphodontia* sensu lato with each other and other main lineages within *Hymenochaetales*. All vouchers of *Hymenochaetales* listed in Table S1, each with at least both ITS and nLSU sequences available, were included as ingroup taxa. Moreover, even nLSU sequences unavailable, the generic types *Chaetoporellus latitans* (a synonym of *Kneiffiella abdita*) and *Hyphodontia pallidula* were also included to confirm phylogenetic position of these genera. In addition, members of *Polyporales* and *Thelephorales* listed in Table S1 were included also as additional ingroup taxa, and *Auricularia cornea* was included as an outgroup taxon. (3–5) As certain recently speciated wood-inhabiting fungi may have nearly congruent ribosomal DNA sequences [44], three multilocus (six to seven genes) combined datasets for *Hyphodontia*, *Lyomyces* and *Xylodon* were separately used to supplementally differentiate species identities within each of these genera. All newly studied specimens with at least two genes available from these three genera, and the collections of *Xylodon* with *tef1α* and *rpb2* sequences downloaded from GenBank were included (Table S1). (6) The combined dataset of ITS, nLSU, *tef1α*, *rpb1* and *rpb2* regions was selected for estimating divergence times of families within *Hymenochaetales*. The vouchers of (described or undescribed) species with at least two (mostly three to five) of these five genes available in families of *Hymenochaetales* were included (Table S1), besides those having been normally used for molecular clock analysis in previous analyses and representing all main lineages in *Basidiomycota* (Table S2). *Neurospora crassa* from *Ascomycota* was designated as an outgroup taxon. (7) Finally, the combined dataset of ITS, nLSU and mt-SSU regions was employed for ancestral state reconstruction. A single example of each (described and undescribed) species with sequences of all these three genes available and a confirmed taxonomic position at the family level within *Hymenochaetales* was selected from the dataset (2) with *Auricularia cornea* as an outgroup taxon (Table S1).

All datasets were aligned using MAFFT 7.110 [57] under the G-INS-i option [58]. Regarding combined multilocus datasets, each locus was aligned separately and then concatenated as a single alignment. All resulting alignments were deposited in TreeBASE (http://www.treebase.org; accessed on 18 May 2021). jModelTest [59,60] with calculation under Akaike information criterion was used to estimate the best-fit evolutionary model for each alignment subjected to phylogenetic analysis.

Maximum likelihood (ML) and Bayesian inference (BI) algorithms were utilized for phylogenetic analyses of the alignments of datasets (1–5). The ML algorithm was conducted using raxmlGUI 1.2 [61,62] with the calculation of bootstrap (BS) replicates under the auto FC option [63]. The BI algorithm was conducted using MrBayes 3.2 [64]. Two independent runs were employed, each including four chains and starting from random trees. The first 25% of the sampled trees every 1000th generation were removed, and the other 75% of trees were retained for constructing a 50% majority consensus tree and calculating Bayesian posterior probabilities (BPPs). Tracer 1.5 (http://tree.bio.ed.ac.uk/software/tracer/, accessed on 18 May 2021) was used to judge whether chains converged.

Molecular clock analysis for the alignment of dataset (6) was performed using BEAST v2.6.0 [65]. The lognormal relaxed molecular clock model and the Yule speciation prior were set to evaluate the divergence times and their corresponding credibility intervals. Four time points were selected for calibration: (1) 90 million years ago (Mya) representing the minimum age of *Agaricales* by *Archaeomarasmius leggetti*, a fossil agaricoid species preserved in a Dominican amber [66,67]; (2) 125 Mya representing the minimum age of *Hymenochaetaceae* by *Quatsinoporites cranhamii*, a fossil poroid species collected from Apple Bay on Vancouver Island [68,69]; (3) 400 Mya representing the divergence time between *Ascomycota* and *Basidiomycota* by *Paleopyrenomycites devonicus*, a fossil fungi found in Great Britain [70,71]; and (4) 290 Mya representing the mean age of *Agaricomycetes* by the analyses of genome data [72]. According to these time points, the offset age with a gamma distribution prior (scale = 20, shape = 1) for *Agaricales* was set as 90 Mya, for *Hymenochaetaceae* as 125 Mya, and for *Basidiomycota* as 400 Mya, while the mean age with a normal distribution prior (SD = 1) for *Agaricomycetes* was set as 290 Mya. After 400 million generations, the first 10% of the sampled trees every 1000th generation were removed as burn-in. The resulting log file was checked for chain convergence using Tracer 1.5.

A consensus tree for the alignment of dataset (7) was generated by BI algorithm with 50 million generations and the first 10% of the sampled trees every 1000th generation as burn-in using BEAST v1.10.4 [73] and then used for ancestral state reconstruction. The resulting log file was checked for chain convergence using Tracer 1.5. The trait evolution of basidiocarps was evaluated using RASP 4.2 under the Bayesian Binary MCMC model [74,75]. Two kinds of morphological traits of basidiocarps were set for selected species: one is the basidiocarp shape, including pileate, pileate-resupinate and resupinate states; the other is hymenophoral configuration, including poroid, smooth, grandinioid and odontioid states.

## 3. Results

In this study, 277 specimens belonging to *Hyphodontia* sensu lato were newly examined and sequenced. Moreover, 20 additional specimens outside *Hyphodontia* sensu lato were also newly sequenced for phylogenetic analyses. From these 297 specimens, we generated 285 ITS, 269 nLSU, 154 mt-SSU, 54 *tef1α*, 50 *rpb1*, 72 *rpb2* and 20 *atp6* sequences (Table S1).

**Figure 1:**
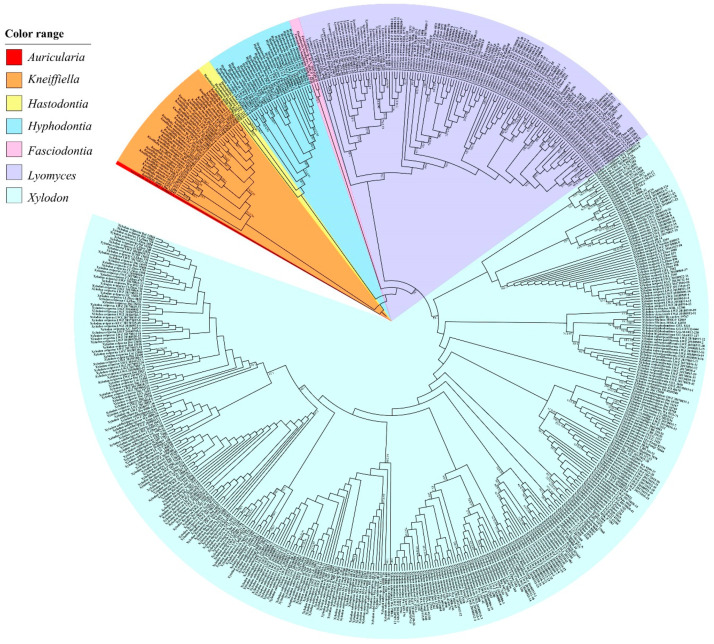
Identity of species belonging to Hyphodontia sensu lato differentiated by ITS-based phylogeny. The tree generated by the maximum likelihood algorithm is presented along with the bootstrap values and the Bayesian posterior probabilities above 50% and 0.8, respectively, at the nodes. The genus represented by each color is indicated in the upper-left of the tree.

The ITS dataset (1) including 582 collections resulted in an alignment of 925 characters with GTR+I+G as the best-fit evolutionary model. The ML search stopped after 350 BS replicates. In BI, after 50 million generations with an average standard deviation of split frequencies of 0.004289, all chains converged which was indicated by the effective sample sizes (ESSs) above 1400 and the potential scale reduction factors (PSRFs) close to 1.000. ML and BI algorithms generated similar topologies in main lineages with minor differences in statistical supports. Therefore, the tree generated by the ML algorithm was presented along with BS value above 50% and BPPs above 0.8 at the nodes (Figure 1). In general, the ITS-based phylogeny delimited species well; at the generic rank, *Hyphodontia* and *Fasciodontia* were strongly supported (BS > 90%, BPP = 1), while *Hastodontia*, *Kneiffiella*, *Lyomyces* and *Xylodon* did not receive reliable support; the clade of *Lyomyces* and *Xylodon* (BS = 74%, BPP = 1) was moderately supported, while no affinity among genera was clarified with strong support (Figure 1). Among the genera *Hyphodontia*, *Kneiffiella*, *Lyomyces* and *Xylodon*, 28 undescribed independent lineages emerged from the newly studied specimens, of which 14 lineages are composed of at least two specimens and 14 are single-specimen lineages.

**Figure 2:**
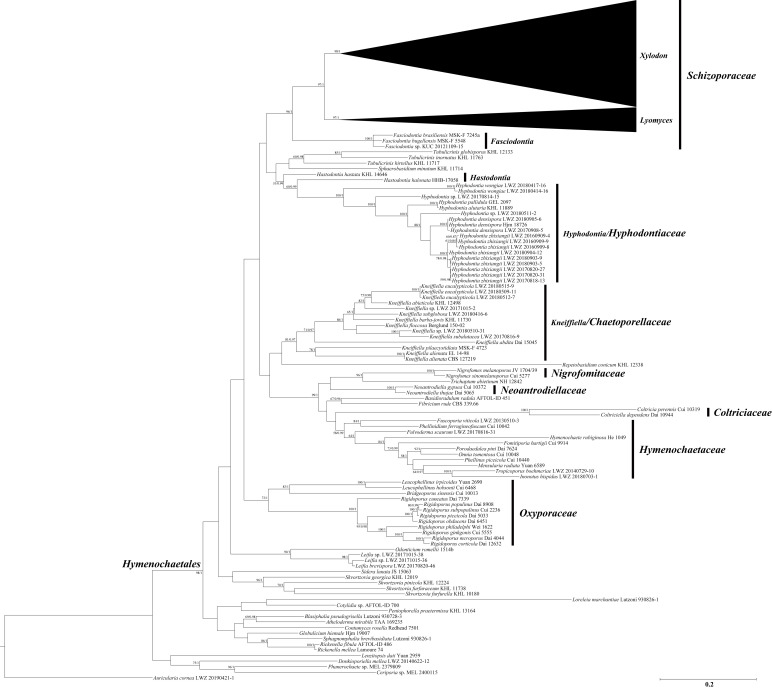

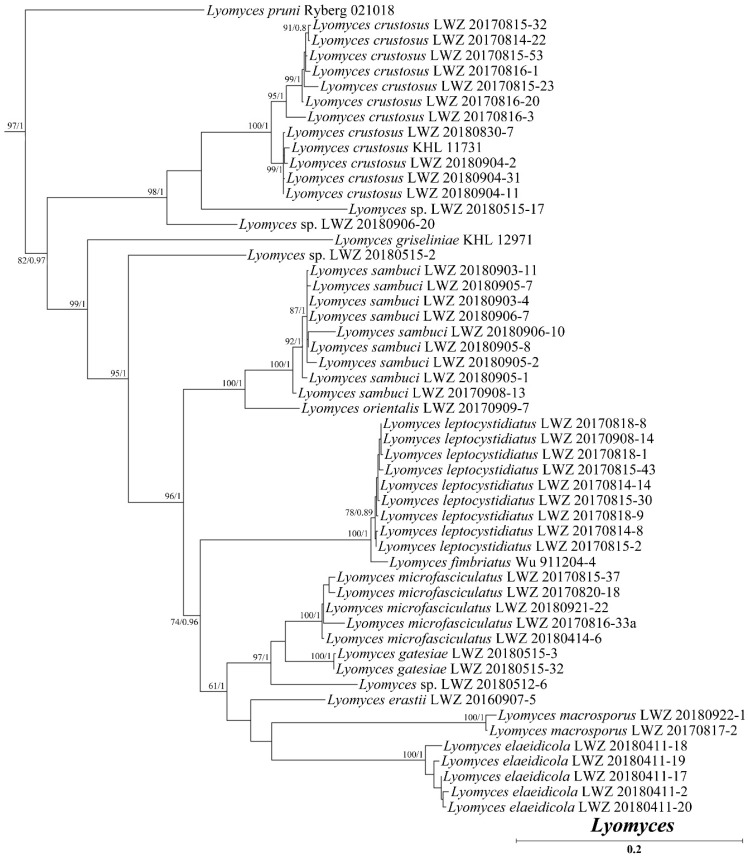

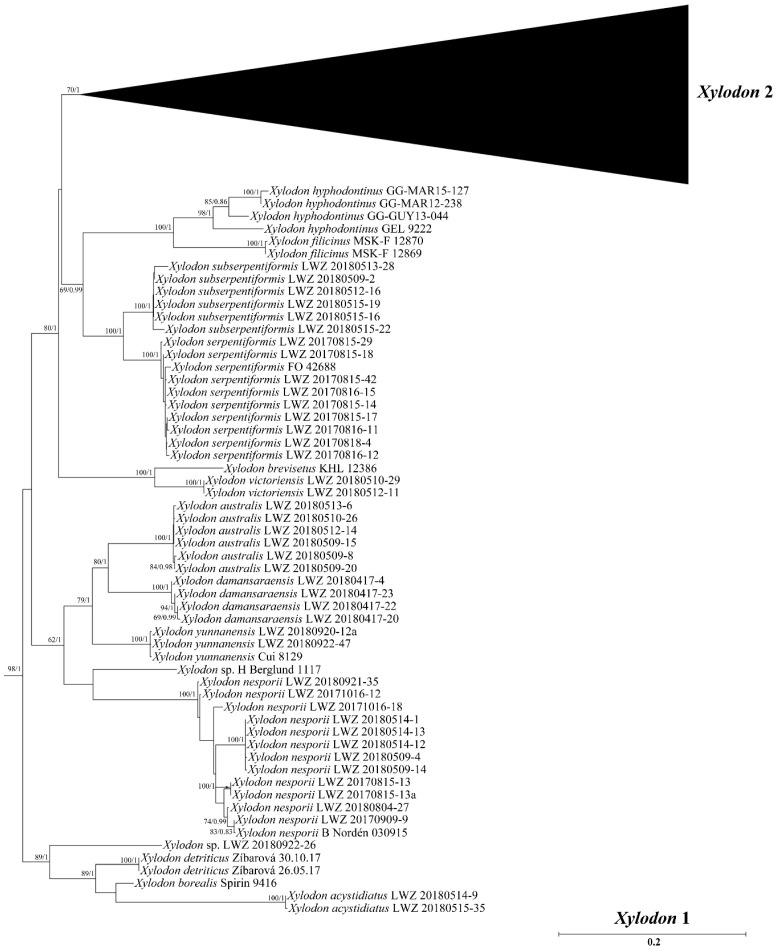

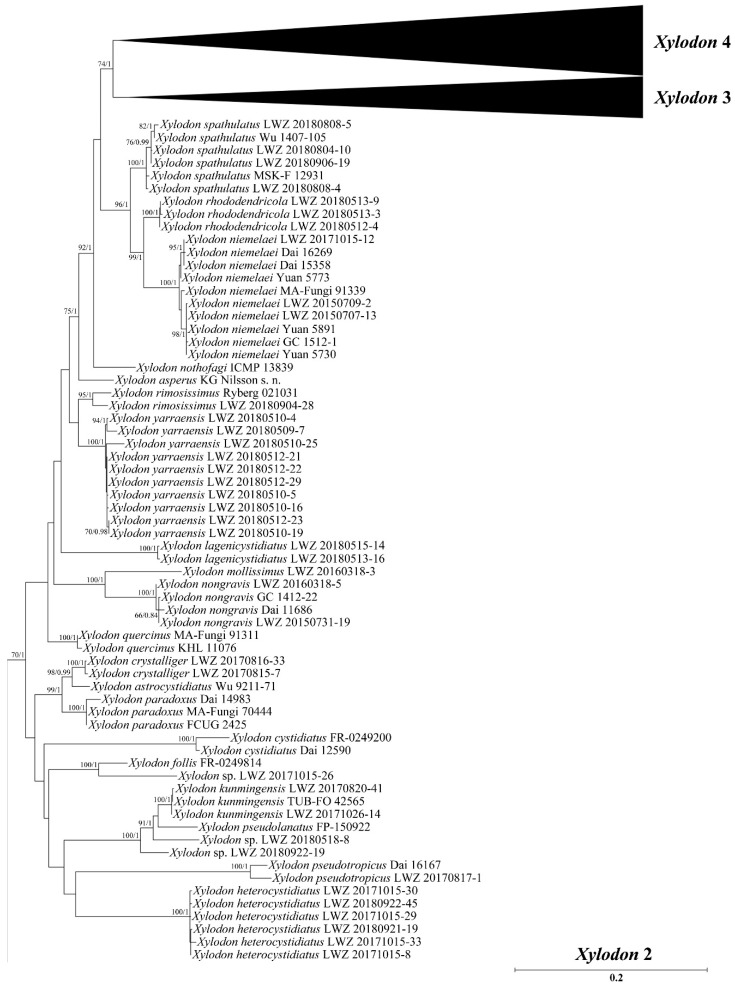

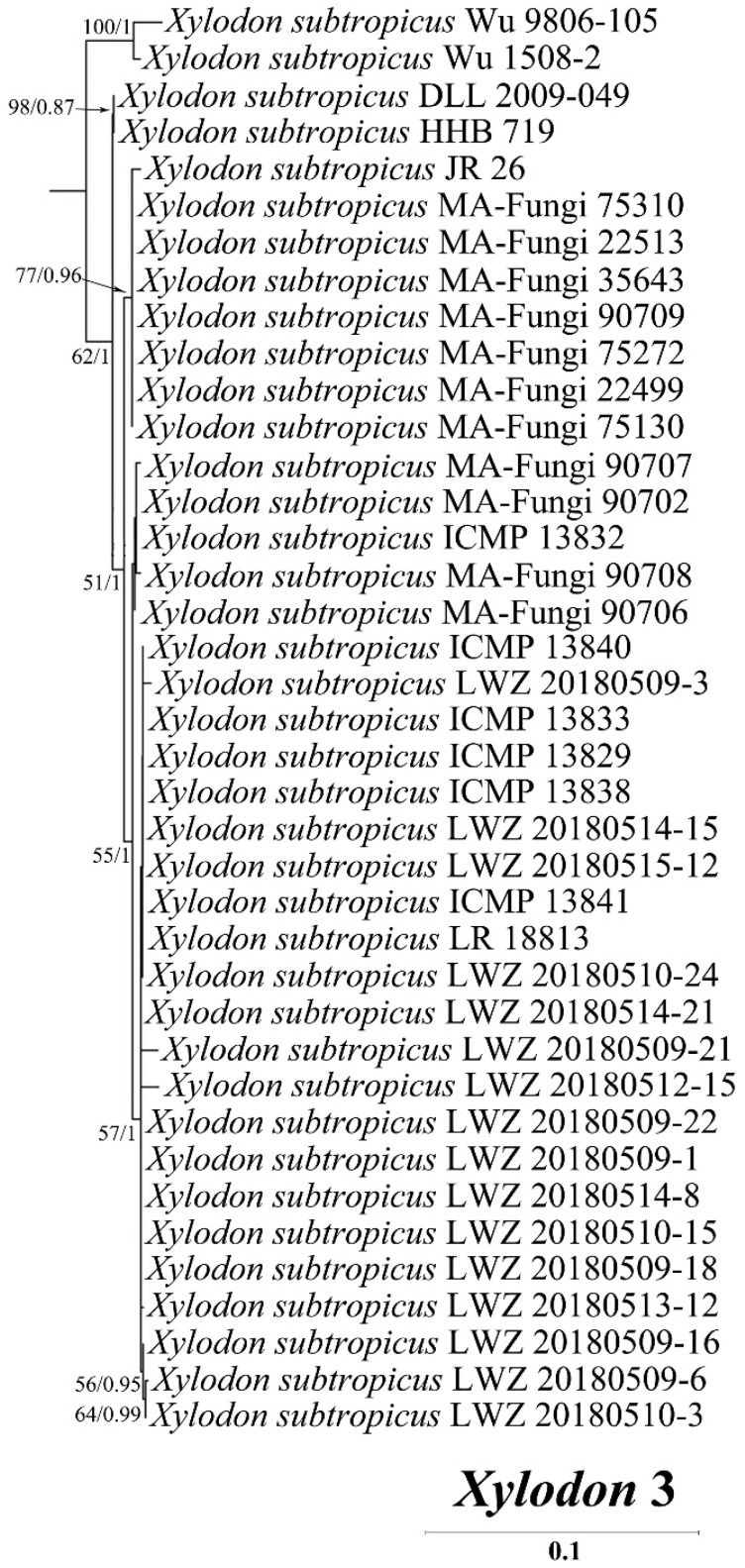

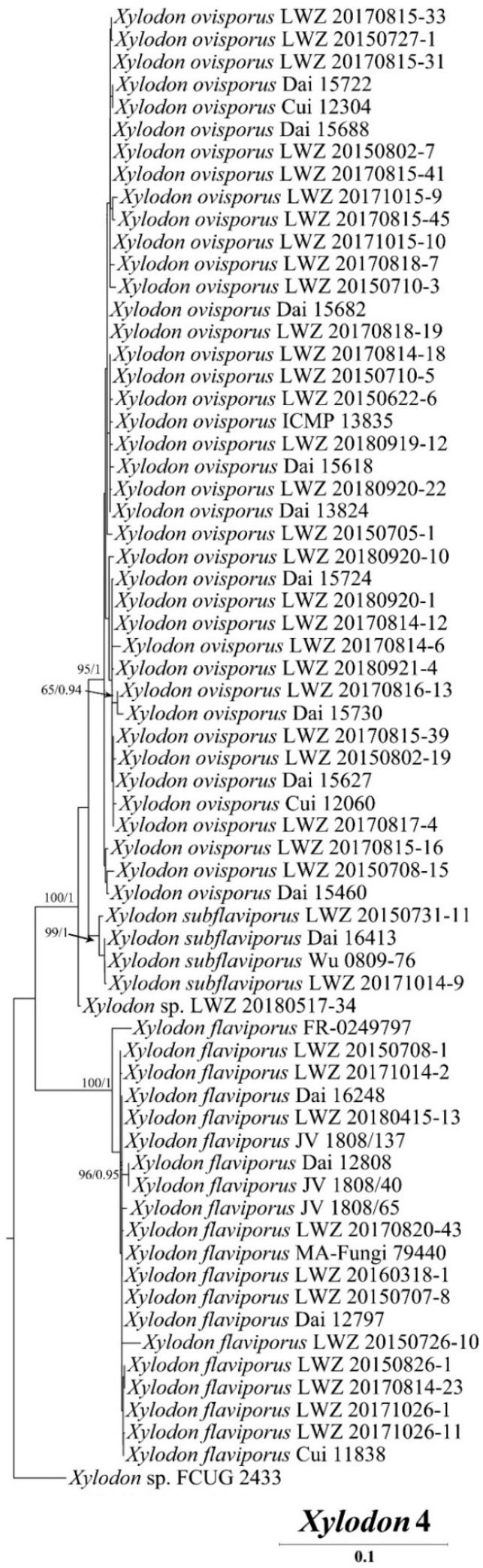
Phylogenetic relationships of species belonging to *Hyphodontia* sensu lato within *Hymenochaetales* inferred from the combined dataset of ITS, nLSU and mt-SSU regions. The topology generated by the maximum likelihood algorithm is presented along with the bootstrap values and the Bayesian posterior probabilities above 50% and 0.8, respectively, at the nodes.

The combined dataset of ITS, nLSU and mt-SSU regions (2) including 380 collections resulted in a concatenated alignment of 3425 characters with GTR+I+G as the best-fit evolutionary model. The ML search stopped after 250 BS replicates. In BI, all chains converged after 50 million generations with an average standard deviation of split frequencies of 0.003928, which was indicated by the ESSs above 2690 and the PSRFs close to 1000. ML and BI algorithms generated similar topologies in main lineages, and thus only the topology generated by the ML algorithm is presented along with BS value and BPPs above 50% and 0.8, respectively, at the nodes (Figure 2). The phylogeny generated by this dataset strongly supports *Hymenochaetales* as an independent order (BS = 98%, BPP = 1). Within *Hymenochaetales*, five other families, viz. *Coltriciaceae*, *Hymenochaetaceae*, *Neoantrodiellaceae*, *Nigrofomitaceae* and *Oxyporaceae* are strongly (BS = 100%, BPP = 1) or moderately (BS > 58%, BPP > 0.99) supported as five monophyletic lineages, while the six genera belonging to *Hyphodontia* sensu lato are all strongly supported as independent genera. Of the six genera, *Fasciodontia*, *Lyomyces* and *Xylodon* form a strongly supported clade (BS = 96%, BPP = 1), while *Hyphodontia*, *Kneiffiella* and *Hastodontia* represent independent lineages within *Hymenochaetales*. As *Schizopora*, the type genus of *Schizoporaceae*, is a later synonym of *Xylodon*, the clade including *Fasciodontia*, *Lyomyces* and *Xylodon*, recognized at the family level, should be called *Schizoporaceae*. Similarly, *Chaetoporellus* is a later synonym of *Kneiffiella*, so the family name *Chaetoporellaceae* typified by *Chaetoporellus* was reintroduced for the well supported clade of *Kneiffiella* (BS = 81%, BPP = 0.97). The clade of *Hyphodontia* (BS = 100%, BPP = 1) outside of previously arranged families *Chaetoporellaceae* and *Schizoporaceae* are described as one new family on the basis of this genus. *Hastodontia* is considered to be a genus with an uncertain taxonomic position at the family rank within *Hymenochaetales*, because at the moment it includes two species that were separated in the current phylogenies (Figure 1 and Figure 2). The 14 novel species-level lineages each with at least two specimens identified by the ITS-based phylogeny (Figure 1) also formed in the same genera in this combined dataset. They are described as 14 new species also taking morphological characters into consideration.

**Figure 3:**
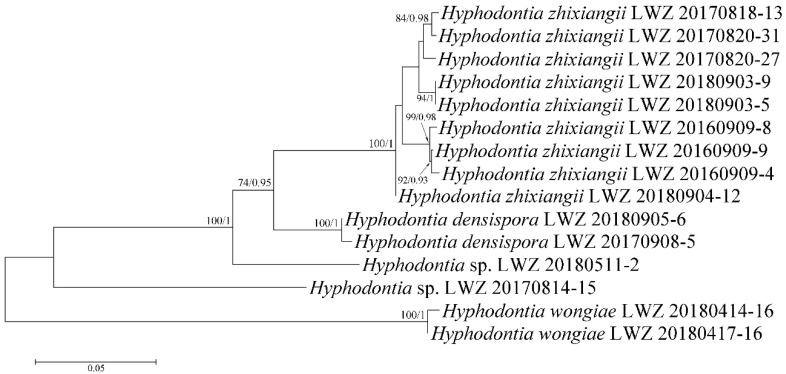
Phylogenetic relationship among species of *Hyphodontia* inferred from the combined dataset of ITS, nLSU, mt-SSU, *tef1α*, *rpb1* and *rpb2* regions. The topology generated by the maximum likelihood algorithm is presented along with the bootstrap values and the Bayesian posterior probabilities above 50% and 0.8, respectively, at the nodes.

**Figure 4:**
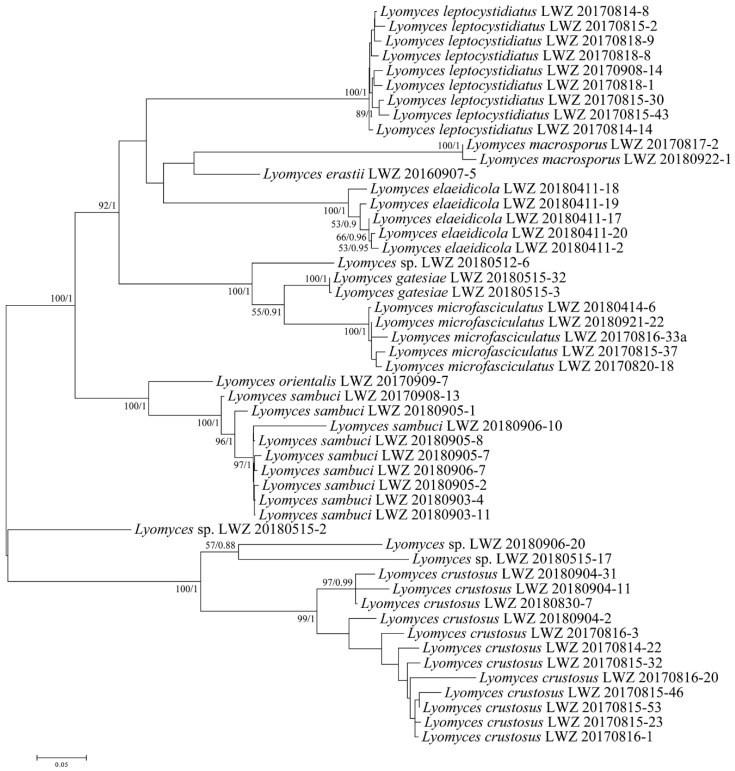
Phylogenetic relationship among species of *Lyomyces* inferred from the combined dataset of ITS, nLSU, mt-SSU, *tef1α*, *rpb1*, *rpb2* and *atp6* regions. The topology generated by the maximum likelihood algorithm is presented along with the bootstrap values and the Bayesian posterior probabilities above 50% and 0.8, respectively, at the nodes.

**Figure 5:**
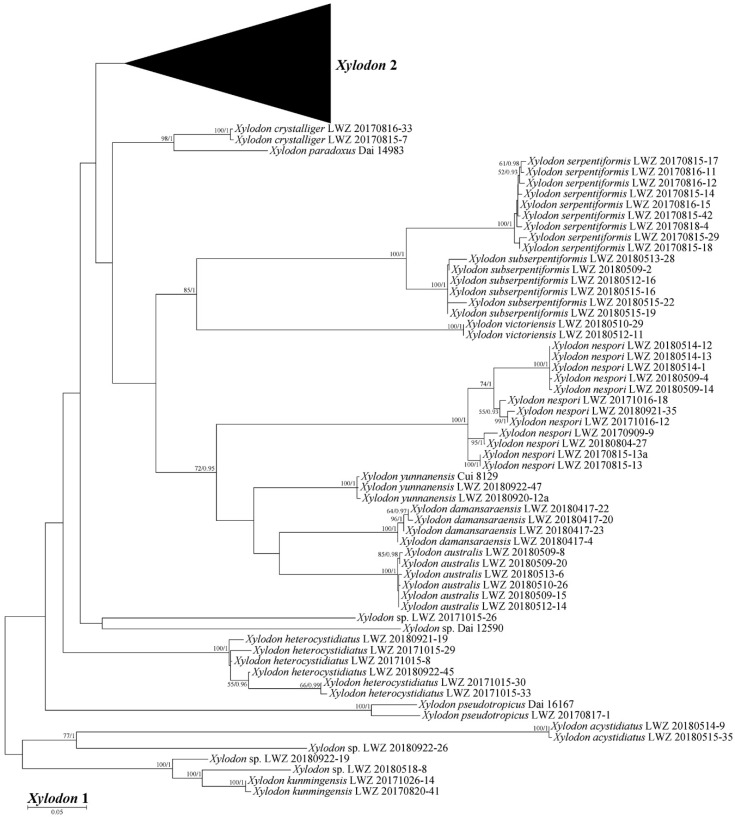

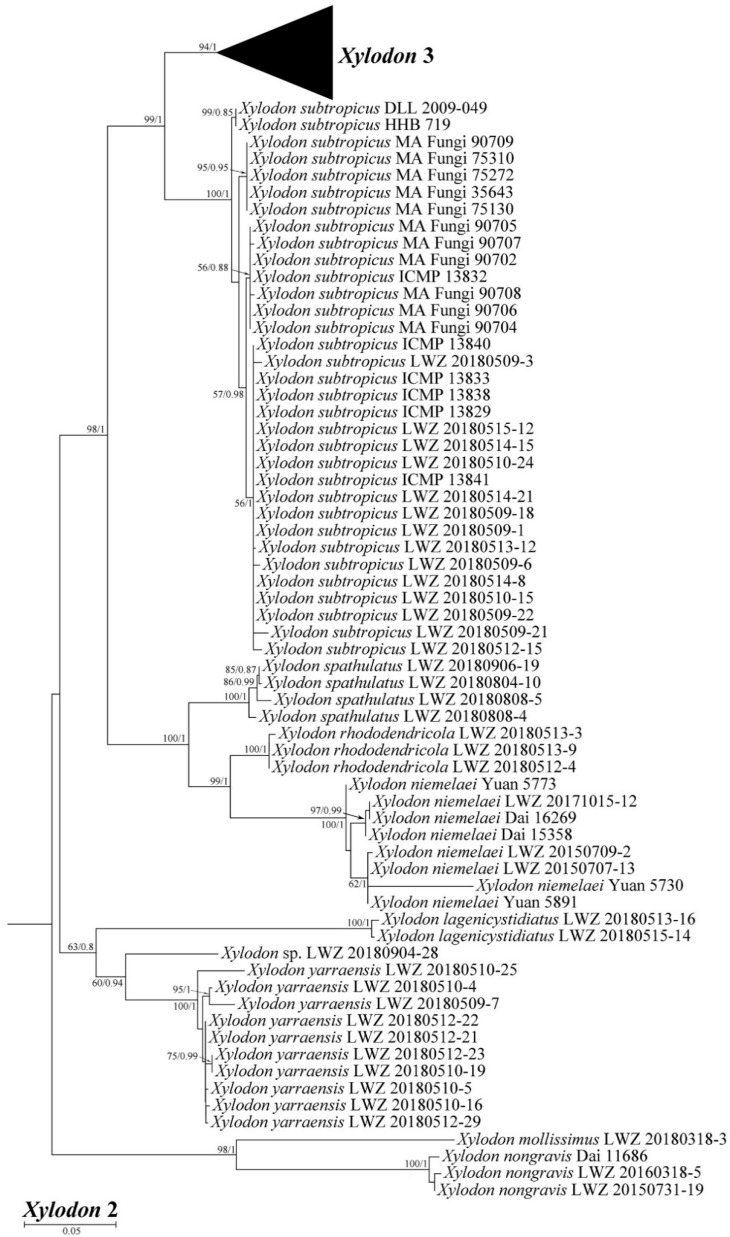

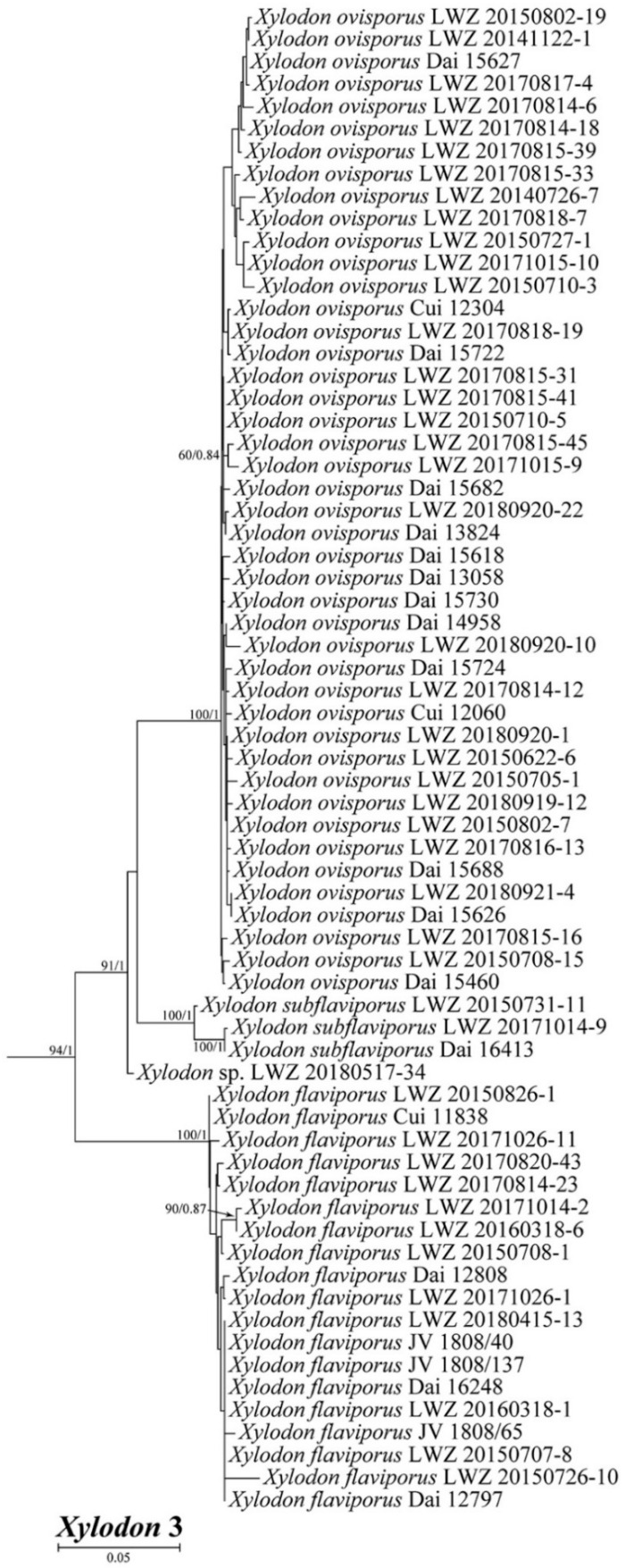
Phylogenetic relationship among species of *Xylodon* inferred from the combined dataset of ITS, nLSU, mt-SSU, *tef1α*, *rpb1*, *rpb2* and *atp6* regions. The topology generated by the maximum likelihood algorithm is presented along with the bootstrap values and the Bayesian posterior probabilities above 50% and 0.8, respectively, at the nodes.

With regard to the multilocus combined datasets, that of ITS, nLSU, mt-SSU, *tef1α*, *rpb1* and *rpb2* regions for *Hyphodontia* (3) including 15 collections resulted in a concatenated alignment of 5060 characters with GTR+G as the best-fit evolutionary model. The ML search stopped after 250 BS replicates. In BI, all chains converged after ten million generations with an average standard deviation of split frequencies of 0.002287, which was indicated by the ESSs above 5300 and the PSRFs equal to 1.000. The multilocus combined dataset of ITS, nLSU, mt-SSU, *tef1α*, *rpb1*, *rpb2* and *atp6* regions for *Lyomyces* (4) including 50 collections resulted in a concatenated alignment of 5342 characters with GTR+I+G as the best-fit evolutionary model. The ML search stopped after 300 BS replicates. In BI, all chains converged after ten million generations with an average standard deviation of split frequencies of 0.002946, which was indicated by the ESSs above 4400 and the PSRFs close to 1.000. The multilocus combined dataset of ITS, nLSU, mt-SSU, *tef1α*, *rpb1*, *rpb2* and *atp6* regions for *Xylodon* (5) including 194 collections resulted in a concatenated alignment of 5543 characters with GTR+I+G as the best-fit evolutionary model. The ML search stopped after 400 BS replicates. In BI, all chains converged after 15 million generations with an average standard deviation of split frequencies of 0.006113, which was indicated by the ESSs above 1000 and the PSRFs equal to 1.000. Regarding each of the multilocus combined datasets for *Hyphodontia*, *Lyomyces* and *Xylodon*, ML and BI algorithms generated similar topologies in main lineages, and thus only the topologies generated by ML algorithm are presented along with BS value and BPPs above 50% and 0.8, respectively, at the nodes. Based on the multilocus phylogenetic analyses, the midpoint-rooted phylogeny of *Hyphodontia* recovered three species (including two new) and two undescribed single-specimen lineages (Figure 3), that of *Lyomyces* recovered nine species (including three new) and four undescribed single-specimen lineages (Figure 4), while that of *Xylodon* recovered 24 species (including eight new) and seven undescribed single-specimen lineages (Figure 5).

Taking morphological characters and the phylogenies from the datasets 1–5 into consideration, one new family and 14 species are proposed, and the taxonomic status of an additional 30 species names is discussed with the inclusion of validating two ineffectively published, invalid names as two new species of *Xylodon*. Moreover, identification keys to each of the genera of *Hyphodontia*, *Kneiffiella*, *Lyomyces* and *Xylodon* are provided.

**Figure 6:**
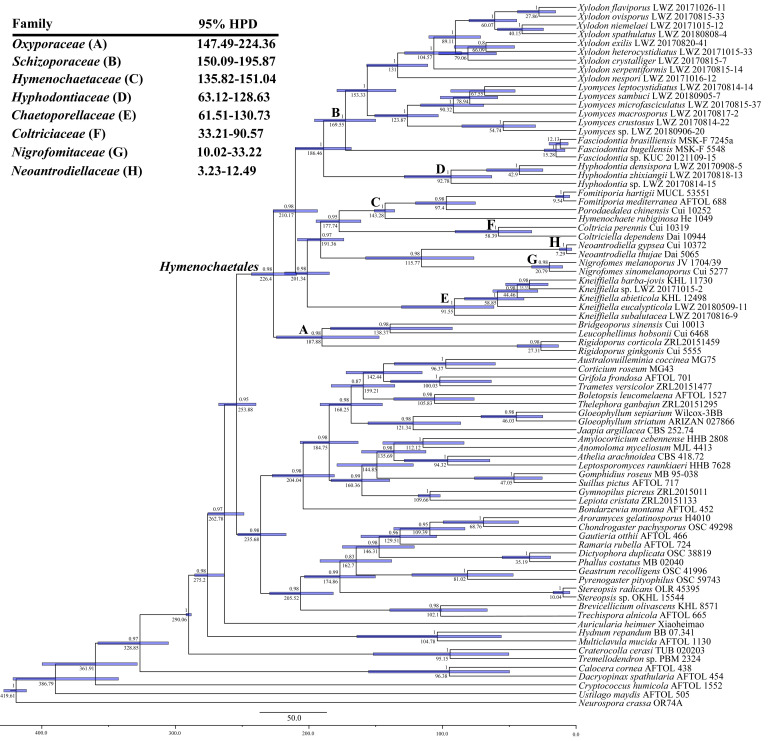
Maximum-clade-credibility chronogram and estimated divergence times of families within *Hymenochaetales* inferred from the combined dataset of ITS, nLSU, *tef1α*, *rpb1* and *rpb2* regions. The estimated divergence times of 95% highest posterior density for all clades were indicated as node bars and for families in *Hymenochaetales* were also provided in the upper-left of the tree as exact numbers, while the Bayesian posterior probabilities above 0.8 and the mean divergence times of clades were labeled above and below the branches, respectively, at the nodes.

The combined dataset for molecular clock analysis (6) included 80 collections, of which 40 belonged to *Hymenochaetales*. This dataset resulted in a concatenated alignment of 8330 characters with GTR+I+G as the best-fit evolutionary model. Chain convergence was indicated by the ESSs above 2010. In *Hymenochaetales*, the youngest family is *Neoantrodiellaceae* occurring in a mean crown age of 7.29 Mya with a 95% highest posterior density (HPD) of 3.23–12.49 Mya, followed by *Nigrofomitaceae* with a mean crown age of 20.79 Mya and a 95% HPD of 10.02–33.22 Mya and *Coltriciaceae* with a mean crown age of 58.39 Mya and a 95% HPD of 33.21–90.57 Mya (Figure 6)*. Chaetoporellaceae* (mean crown age 91.55 Mya) and *Hyphodontiaceae* (mean crown age 92.78 Mya) emerged between 61.51 and 130.73 Mya (95% HPD). *Hymenochaetaceae* emerged earlier with a mean crown age of 143.28 Mya and a 95% HPD of 135.82–151.04 Mya, while *Schizoporaceae* (mean crown age 169.55 Mya) and *Oxyporaceae* (mean crown age 187.88 Mya) older than all these other families share the overlapping time period with a 95% HPD of 147.49–224.36 Mya.

**Figure 7:**
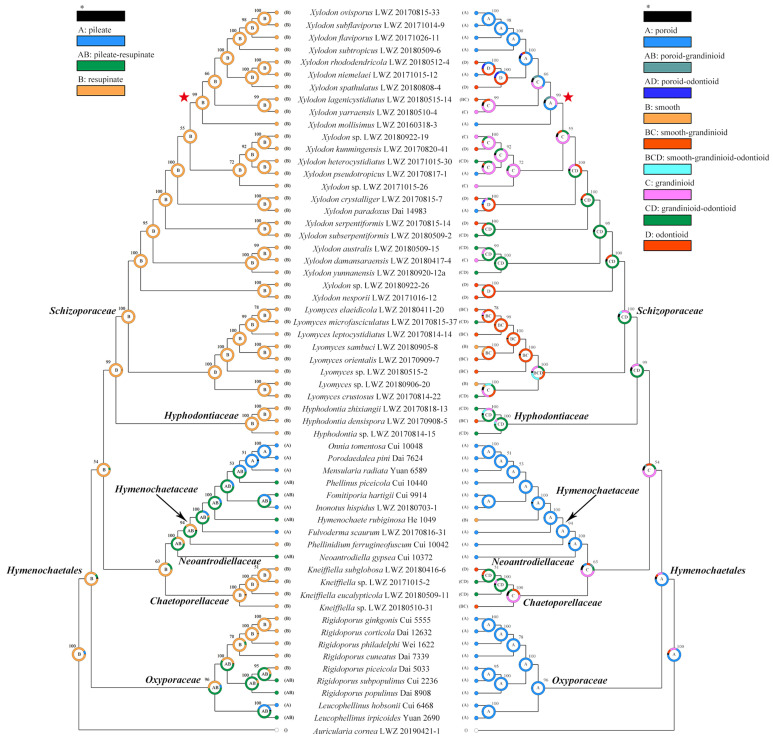
Trait evolution of basidiocarps within *Hymenochaetales*. The mirrored consensus tree was generated by the Bayesian inference algorithms using BEAST, while the trait of a pie chart at each node was evaluated using RASP under the Bayesian Binary MCMC model. The trait represented by each color and letter in the pie chart is indicated in the upper-left for the left part and upper-right of the right part. The lineage for each family is indicated at the nodes along with the pentagram mark for assisting discussion.

The combined dataset for ancestral state reconstruction (7) including 59 collections resulted in a concatenated alignment of 2436 characters with GTR+I+G as the best-fit evolutionary model. Chain convergence was indicated by the ESSs above 290. Across the two basidiocarp traits, the resupinate habit and poroid hymenophoral configuration were evaluated as the ancestral state within *Hymenochaetales* (Figure 7). Below the order level, the resupinate habit remains in all families but also evolves to the pileate habit in *Hymenochaetaceae*, *Neoantrodiellaceae* and *Oxyporaceae* as pileate-resupinate basidiocarps. The ancestral poroid state in *Hymenochaetales* remains only in *Oxyporaceae*, and evolves to grandinioid state in *Chaetoporellaceae* and grandinioid-odontioid state in *Hyphodontiaceae* and *Schizoporaceae*. Noteworthily, the poroid state in *Hymenochaetaceae* and *Neoantrodiellaceae* seems to independently evolve back from the grandinioid state and be related to the emergence of a pileate habit in this lineage.

## 4. Taxonomy

***Chaetoporellaceae*** Jülich, Biblthca Mycol. 85: 359. 1982 (1981).

**Type genus:***Chaetoporellus* Bondartsev & Singer, in Singer, Mycologia 36(1): 66. 1944.

= *Kneiffiella* P. Karst., Bidr. Känn. Finl. Nat. Folk 48: 371. 1889.

**Description:** Basidiocarps resupinate. Hymenophore smooth, floccose, tuberculate, grandinioid, coralloid, odontioid or irpicoid; whitish, creamish, yellowish, grey, buff, brown or ochraceous. Hyphal system monomitic to pseudodimitic. Hyphae with clamp connections. Tubular tramacystidia present. Basidia clavate, cylindrical, utriform or barrel-like, with four sterigmata. Basidiospores cylindrical, ellipsoid or allantoid, smooth, hyaline, thin-walled or slightly thick-walled, inamyloid, acyanophilous.

***Kneiffiella*** P. Karst., Bidr. Känn. Finl. Nat. Folk 48: 371. 1889.

= *Chaetoporellus* Bondartsev & Singer, in Singer, Mycologia 36(1): 66. 1944.

= *Alutaceodontia* (Parmasto) Hjortstam & Ryvarden, Syn. Fung. (Oslo) 15: 7. 2002.

= *Deviodontia* (Parmasto) Hjortstam & Ryvarden, Syn. Fung. (Oslo) 26: 49. 2009.

**Type species:***Kneiffiella barba-jovis* (Bull.) P. Karst. (as ‘*barba-jobi*’), Bidr. Känn. Finl. Nat. Folk 48: 371. 1889.

**Description:** Basidiocarps resupinate. Hymenophore smooth, floccose, tuberculate, grandinioid, coralloid, odontioid or irpicoid; whitish, creamish, yellowish, grey, buff, brown or ochraceous. The hyphal system is monomitic to pseudodimitic. Hyphae with clamp connections. Tubular tram cystidia present in most species. Basidia clavate, cylindrical, utriform or barrel-like, with four sterigmata. Basidiospores cylindrical, ellipsoid or allantoid, smooth, hyaline, thin-walled or slightly thick-walled, inamyloid, acyanophilous.

**Figure 8:**
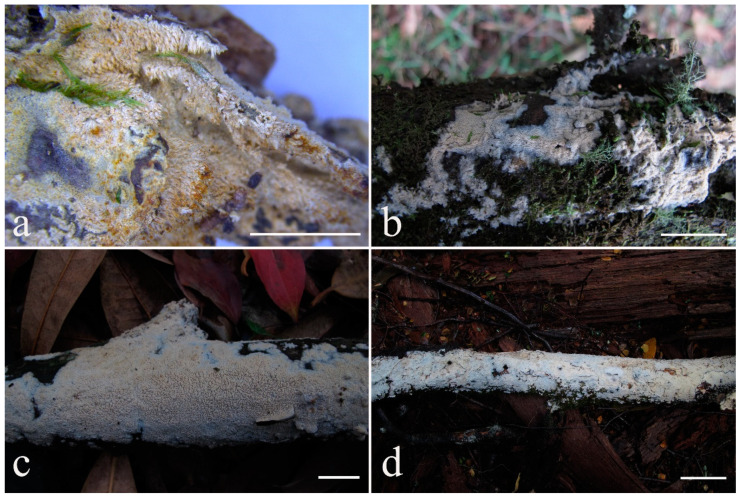
(**a**) Basidiocarps of *Kneiffiella eucalypticola*, (**b**) LWZ 20180509-11 (holotype); (**c**) LWZ 20180512-7 (paratype); (**d**) LWZ 20180515-9 (paratype).—Scale bars: **a** = 5 mm; **b**,**c** = 1 cm; **d** = 2 cm.

**Figure 9:**
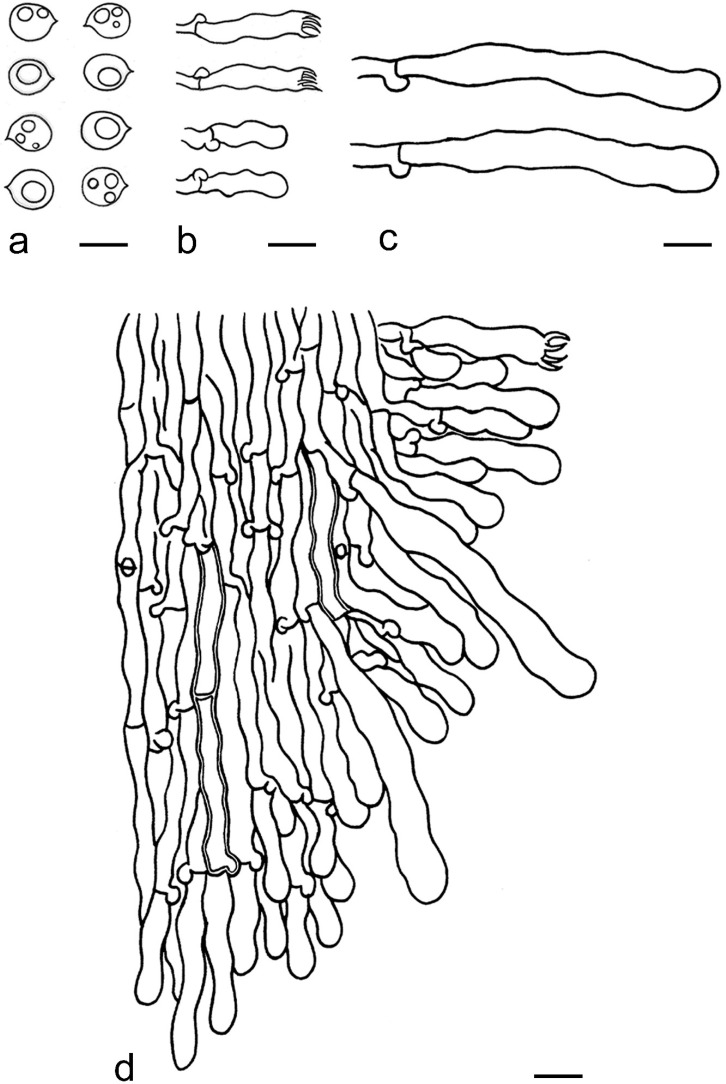
Microscopic structures of *Kneiffiella eucalypticola* (drawn from the holotype). (**a**) Basidiospores; (**b**) basidia and basidioles; (**c**) tramacystidia; (**d**) a section of the basidiocarp.—Scale bars: **a** = 5 µm; **b**–**d** = 10 µm.

***Kneiffiella eucalypticola*** Xue W. Wang & L.W. Zhou, **sp. nov.** (Figure 8 and Figure 9)

**Index Fungorum identifier:** IF 558470

**Etymology:***Eucalypticola* (Latin), refers to the host tree *Eucalyptus*.

**Type:** Australia, Victoria, Yarra Ranges National Park, Dom Dom, on the fallen branch of *Eucalyptus*, 9 May 2018, *L.W. Zhou*, LWZ 20180509-11 (holotype MEL, isotype HMAS).

**Description:** Basidiocarps annual, resupinate, cracked and brittle when dry. Hymenophore grandinioid to odontioid, aculei up to 1 mm long, cream in young parts and buff in old parts. Margin paler than or concolorous with subiculum, abrupt. Hyphal system monomitic; generative hyphae with clamp connections, moderately ramified, hyaline, straight, thin- or slightly thick-walled, 3.5–5.5 μm in diam. Tubular tramacystidia abundant, penetrating approximately half of their lengths through hymenium, hyaline, not encrusted, thin-walled, 25–45 × 4.5–5.5 μm. Basidia clavate to subclavate, 20–30 × 4–5 μm, with four sterigmata and a clamp connection at the base. Basidiospores broadly ellipsoid to ovoid, adaxially flattened, smooth, thin-walled, usually with oily drops, acyanophilous, inamyloid, indextrinoid, (3.5–)3.6–5.1(–5.5) × (2.8–)2.9–3.8(–3.9) μm, L = 3.96 μm, W = 3.31 μm, Q = 1.17–1.22 (90/3).

Other specimens (paratypes) examined: Australia, Victoria, Yarra Ranges National Park, Dandenong Ranges Botanic Garden, on fallen angiosperm branch, 12 May 2018, *L.W. Zhou*, LWZ 20180512-7 (HMAS, paratype); Tasmania, Tahune Adventures, The Look-in Look-out, on fallen angiosperm branch, 15 May 2018, *L.W. Zhou*, LWZ 20180515-9 (HMAS, paratype).

**Notes:** *Kneiffiella eucalypticola* is distinct by grandinioid to odontioid basidiocarps, thin-walled tubular tramacystidia and presence of clamp connections. These characters make *K. eucalypticola* close to *K. alutacea* and *K. curvispora*; however, the latter two species differ by narrowly allantoid and semicircle-like basidiospores, respectively, which are no more than 2 µm wide [19]. *Kneiffiella tubuliformis* also resembles *K*. eucalypticola, but differs by thick-walled tramacystidia and cylindrical to suballantoid basidiospores [76].

***Kneiffiella microspora*** (J. Erikss. & Hjortstam) Jülich & Stalpers, Verh. K. ned. Akad. Wet., tweede sect. 74: 130. 1980.

**Basionym:***Hyphodontia microspora* J. Erikss. & Hjortstam, Cortic. N. Eur. (Oslo) 4: 651. 1976.

= *Odontia palmae* Rick, in Rambo (Ed.), Iheringia, Sér. Bot. 5: 163. 1959, nom. inval.

≡ *Hyphodontia palmae* Langer, Biblthca Mycol. 154: 177. 1994, nom. inval., as ‘Rick ex Langer’.

≡ *Kneiffiella palmae* Hjortstam & Ryvarden, Syn. Fung. (Oslo) 26: 45. 2009, nom. inval., as ‘(Rick ex E. Langer) Hjortstam & Ryvarden’.

**Notes:** *Kneiffiella microspora* and *K. palmae* are conspecific, and the latter was accepted as the correct name of this species [16]. However, *Odontia palmae* was not validly published due to the type not being indicated [77], even if it has priority over *Hyphodontia microspora* the basionym of *K. microspora*. Subsequently, Langer [19] published the name *Hyphodontia palmae*, attempting to validate the name *Odontia palmae* by selecting a lectotype, but the lack of a Latin description makes the name invalid too. Similarly, *Kneiffiella palmae*, an attempted combination based on the invalid *H. palmae* [10], is also invalid. Given the above, the correct name for this species is confirmed as *Kneiffiella microspora*.

***Kneiffiella subefibulata*** (Jia J. Chen & L.W. Zhou) Riebesehl & Langer, Mycol. Progr. 16(6): 650. 2017.

**Basionym:***Hyphodontia subefibulata* Jia J. Chen & L.W. Zhou, in Chen, Zhou, Ji & Zhao, Phytotaxa 269(1): 7. 2016.

**Notes:** Like previous phylogenetic analysis [16], the current ITS-based phylogeny grouped sequences from two collections, each of *Kneiffiella subefibulata* and *K. subglobosa* together in a strongly supported clade (Figure 1). However, the two collections of *K. subefibulata* do not form a separate subclade, but are in a grade at the base of the clade. Morphologically, both species have odontioid to hydnoid hymenophores with 3–5 aculei per mm, aculei up to 2 mm in length and microscopic elements without clamp connection [30,78]. *K. subefibulata*, typified by specimens from Mainland China, bears thin-walled, bladder-like to clavate cystidia (35–55 µm in length) and basidiospores measuring 3.2–4 µm in length [78], whereas *K. subglobosa* based on a specimen from Island of Taiwan, has slightly thick-walled, tubular or cylindrical cystidia (40–180 µm in length) and longer basidiospores (4.2–5 µm in length) [30]. The phenomenon that two species have almost identical ITS regions but distinct morphological characters may represent an ongoing allopatric speciation event [79] and was also reported in *Basidioradulum mayi* and *B. tasmanicum* belonging to *Hymenochaetales* [44]. Therefore, we tentatively accept *K. subefibulata* and *K. subglobosa* as independent species.
**A key to 23 accepted species of *Kneiffiella***1a. Hymenophore smooth.................................................................................................................................................................2b. Hymenophore tuberculate, grandinioid, odontioid, coralloid, irpicoid or poroid..............................................................102a. Basidia with two, rarely three sterigmata..................................................................................................................*K. efibulata*b. Basidia with four sterigmata.........................................................................................................................................................33a. Without clamp connections.........................................................................................................................................................4b. With clamp connections.................................................................................................................................................................54a. Hymenophore cream; basidiospores less than 5 µm long, less than 2.5 µm wide..............................................*K. tetraspora*b. Hymenophore whitish to ochraceous or slightly ochre-brown; basidiospores more than 5 µm long, more than 2.5 µm wide..........................................................................................................................................................................*K. crassispora*5a. Basidiospores less than 6 μm long..............................................................................................................................................6b. Basidiospores more than 6 μm long.............................................................................................................................................86a. Basidiospores cylindrical to allantoid; tramacystidia without exudate....................................................................*K. altaica*b. Basidiospores ellipsoid or cylindrical to suballantoid; tramacystidia sometimes apically with exudate........................77a. Basidiospores less than 3 μm wide.......................................................................................................................*K. microspora*b. Basidiospores more than 3 μm wide............................................................................................................................*K. alienata*8a. Basidiospores less than 2.5 μm wide...................................................................................................................*K. subalutacea*b. Basidiospores more than 2.5 μm wide........................................................................................................................................99a. Hymenophore white to slightly cream; tramacystidia without crystals............................................................*K. cineracea*b. Hymenophore creamish white to slightly yellow, light ochre in spots; tramacystidia smooth or encrusted, sometimes with simple secondary septa............................................................................................................*K. decorticans*10a. Without clamp connections....................................................................................................................................................11b. With clamp connections..............................................................................................................................................................1611a. Basidiospores less than 5 μm long.........................................................................................................................................12b. Basidiospores more than 5 μm long..........................................................................................................................................1512a. Hymenophore odontioid or irpicoid.....................................................................................................................................13b. Hymenophore slightly grandinioid or tuberculate to grandinioid......................................................................................1413a. Hymenophore odontioid, fresh cream-colored, dry buff or dark brown; basidiospores less than 4 μm long.........................................................................................................................................................................*K. subefibulata*b. Hymenophore odontioid to irpicoid, white; basidiospores more than 4 μm long..........................................*K. subglobosa*14a. Hymenophore tuberculate to grandinioid, yellowish brown to ochraceous; cystidia without exudate; basidiospores more than 2.5 μm wide..................................................................................................................*K. byssoidea*b. Hymenophore slightly grandinioid, cream; cystidia with exudate; basidiospores less than 2.5 μm wide...*K. tetraspora*15a. Basidia with two or rarely three sterigmata; basidiospores cylindrical to slightly allantoid.........................*K. efibulata*b. Basidia with four sterigmata; basidiospores ellipsoid.........................................................................................*K. crassispora*16a. Capitate cystidia in subiculum present.................................................................................................................................17b. Capitate cystidia in subiculum absent......................................................................................................................................1817a. Basidiospores broadly ellipsoid (Q < 1.5), thin- to slightly thick-walled….......................................................*K. sinensis*b. Basidiospores ellipsoid (Q > 1.5), thin-walled..................................................................................................*K. pilaecystidiata*18a. Tramacystidia thin-walled......................................................................................................................................................19b. Tramacystidia thick-walled........................................................................................................................................................2219a. Hymenophore irpicoid to poroid, white to yellowish; spores narrowly allantoid, spores 3–4 × 0.5–1 μm.....*K. abdita*b. Hymenophore grandinioid to odontioid..................................................................................................................................2020a. Basidiospores narrowly allantoid or semicircle-like, no more than 2 µm wide.............................................................21b. Basidiospores broadly ellipsoid to ovoid, more than 2 µm wide...................................................................*K. eucalypticola*21a. Basidiospores narrowly allantoid, more than 5 µm long; aculei sometimes grouped together then coralloid.......................................................................................................................................................................*K. alutacea*b. Basidiospores semicircle-like, no more than 5 µm long; aculei not grouped....................................................*K. curvispora*22a. Septa of basal hyphae and part of subiculum without clamp connections; short aculei (up to 50 μm) among long aculei (up to 800 μm)...............................................................................................................................................*K. stereicola*b. Clamp connections everywhere; aculei uniform.....................................................................................................................2323a. Basidiospores less than 4 μm wide........................................................................................................................................24b. Basidiospores more than 4 μm wide.........................................................................................................................................2724a. Basidiospores less than 5.5 μm long.................................................................................................................*K. tubuliformis*b. Basidiospores more than 6 μm long..........................................................................................................................................2525a. Basidiospores cylindrical to suballantoid, more than 2.5 μm wide...............................................................*K. decorticans*b. Basidiospores long allantoid, less than 2.5 μm wide..............................................................................................................2626a. Hymenophore odontioid...........................................................................................................................................*K. floccosa*b. Hymenophore slightly grandinioid.......................................................................................................................*K. subalutacea*27a. Basidiospores cylindrical; hymenophore cream to ochraceous, aculei up to 1 mm long..............................*K. abieticola*b. Basidiospores ellipsoid; hymenophore cream, aculei up to 3 mm long............................................................*K. barba-jovis*

***Hyphodontiaceae*** Xue W. Wang & L.W. Zhou, **fam. nov.**

**Index Fungorum identifier:** IF 557193

**Etymology:***Hyphodontiaceae* (Latin), refers to the type genus *Hyphodontia*.

**Type genus:***Hyphodontia* J. Erikss., Symb. bot. upsal. 16(no. 1): 101. 1958.

**Type species:***Hyphodontia pallidula* (Bres.) J. Erikss., Symb. bot. upsal. 16(no. 1): 104. 1958.

**Type specimen:** Poland, on twigs of *Betula alba* and *Pinus silvestris*, Eichler 46 (FH, lectotype).

**Description:** Basidiocarps resupinate. Hymenophore smooth, grandinioid, odontioid or poroid; whitish to yellowish, buff, cream or orange, brownish orange, cinnamon-buff, tawny olive or buckthorn brown. Hyphal system monomitic to pseudodimitic. Hyphae with clamp connections. Cystidia of one or two types: lagenocystidia, apically strongly encrusted; capitate cystidia, often apically encrusted, sometimes septate, with clamp connections at each or some septa. Basidia clavate, capitate, subcylindrical or utriform, with four sterigmata, often with a dextrinoid reaction. Basidiospores ellipsoid, cylindrical, ovoid or subglobose, smooth, thin-walled or slightly thick-walled, hyaline, inamyloid, acyanophilous.

***Hyphodontia*** J. Erikss., Symb. bot. upsal. 16(no. 1): 101. 1958.

**Type species:** *Hyphodontia pallidula* (Bres.) J. Erikss., Symb. bot. upsal. 16(no. 1): 104. 1958.

**Description:** Basidiocarps resupinate. Hymenophore smooth, grandinioid, odontioid or poroid; whitish to yellowish, buff, cream or orange, brownish orange, cinnamon-buff, tawny olive or buckthorn brown. The hyphal system is monomitic to pseudodimitic. Hyphae with clamp connections. Cystidia of one or two types: lagenocystidia, apically strongly encrusted; capitate cystidia, often apically encrusted, sometimes septate, with clamp connections at each or some septa. Basidia clavate, capitate, subcylindrical or utriform, with four sterigmata, often with a dextrinoid reaction. Basidiospores ellipsoid, cylindrical, ovoid or subglobose, smooth, thin-walled or slightly thick-walled, hyaline, inamyloid, acyanophilous.

***Hyphodontia alutaria*** (Burt) J. Erikss., Symb. bot. upsal. 16(no. 1): 104. 1958.

**Basionym:** *Peniophora alutaria* Burt, Ann. Mo. bot. Gdn 12: 332. 1926 (1925).

**Notes:** In a previous study [16], one sequence from each of *Hyphodontia alutaria* and *H. pallidula* clustered together. These two sequences, and two further sequences of *H. alutaria* form a strongly supported clade in the current ITS-based phylogenetic tree, with the sequence of *H. pallidula* falling within the clade rather than at the base (Figure 1), which indicates that these two species may be conspecific. However, we did not have a chance to examine the voucher specimens for the ITS sequences. Moreover, the morphological characters separating these two species are quite distinct [19]. Therefore, for the moment we accept *H. alutaria* and *H. pallidula* as two independent species.

**Figure 10:**
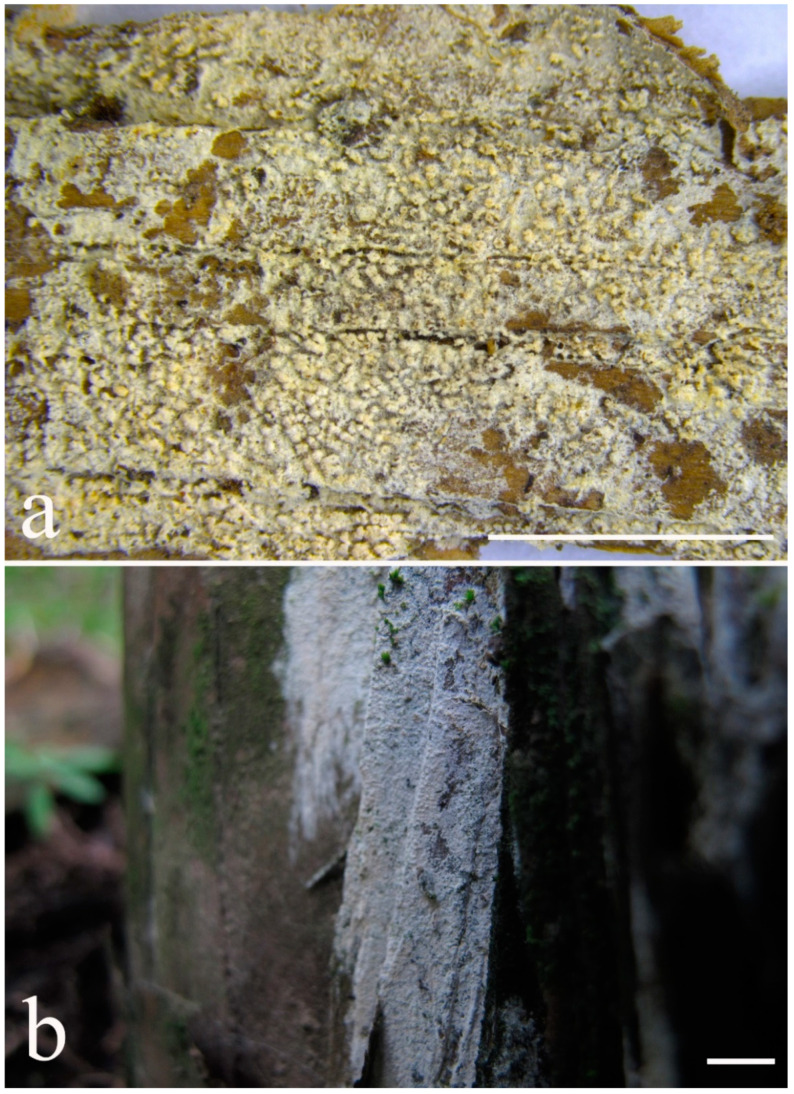
Basidiocarps of *Hyphodontia pachyspora*. (**a**) LWZ 20170908-5 (holotype); (**b**) LWZ 20180905-6 (paratype).—Scale bars: **a** = 5 mm; **b** = 1 cm.

**Figure 11:**
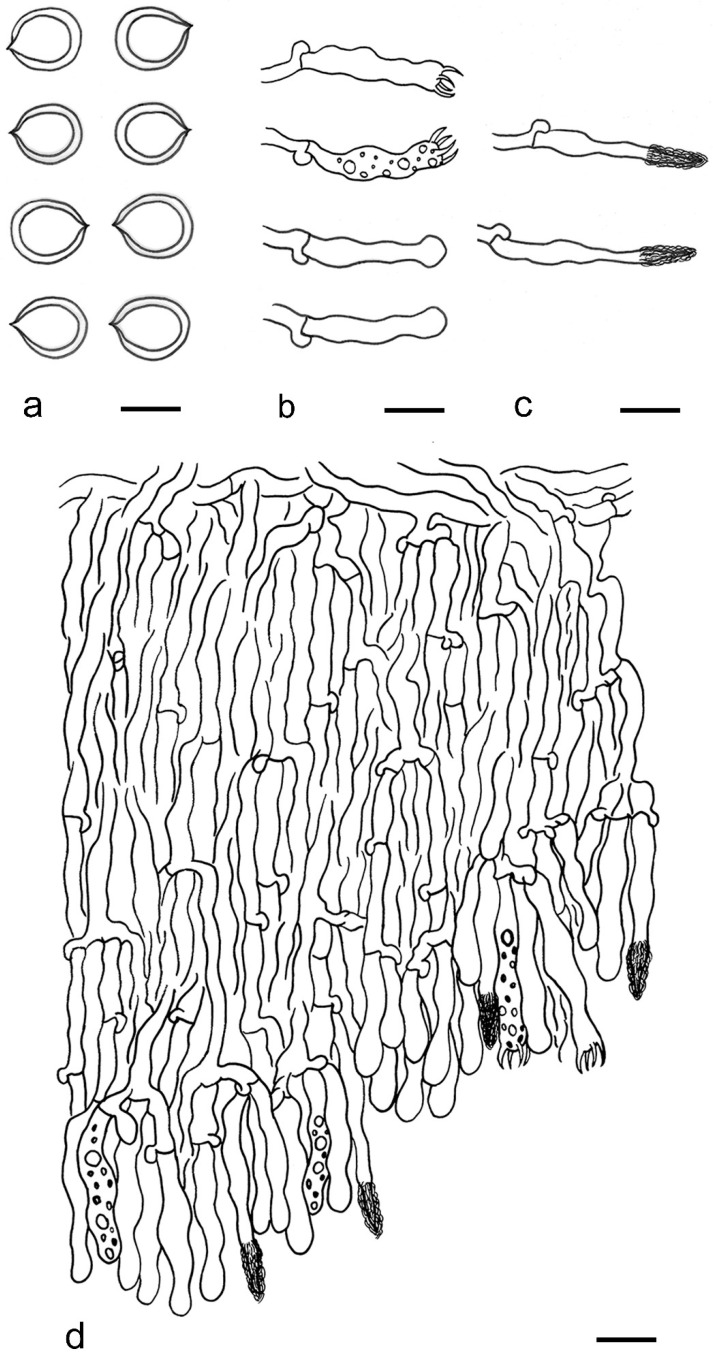
Microscopic structures of *Hyphodontia pachyspora* (drawn from the holotype). (**a**) Basidiospores; (**b**) basidioles; (**c**) lagenocystidia; (**d**) a section of the basidiocarp.—Scale bars: **a** = 5 µm; **b**–**d** = 10 µm.

***Hyphodontia pachyspora*** Xue W. Wang & L.W. Zhou, **sp. nov.** (Figure 10 and Figure 11)

**Index Fungorum identifier:** IF 557223

**Etymology:***Pachyspora* (Latin), refers to the thick-walled basidiospores.

**Type:** China, Beijing, Mentougou, Dongling Mountain, on the base of living angiosperm, 8 Sept. 2017, *L.W. Zhou*, LWZ 20170908-5 (holotype IFP, isotype HMAS).

**Description:** Basidiocarps annual, resupinate, adnate, cracked and brittle when dry. Hymenophore smooth to grandinioid, white to light-buff. Margin paler than or concolorous with subiculum, thinning. Hyphal system monomitic; generative hyphae with clamp connections, hyaline, thin-walled, dichotomous branching, tortuous, 2–3.5 μm in diam. Lagenocystidia abundant, thin-walled, with broad bases tapering abruptly towards the apices, apically encrusted. Basidia cylindrical, somewhat sinuous or utriform, occasionally encrusted with granular crystals, 25–30 × 3.5–5.5 μm, with four sterigmata and a clamp connection at the base; basidioles similar in shape to basidia, but smaller. Basidiospores broadly ellipsoid to ovoid, hyaline, smooth, thick-walled, acyanophilous, inamyloid, indextrinoid, (4.6–)4.7–5.5(–5.6) × (4.1–)4.2–5.1(–5.2) μm, L = 5.17 μm, W = 4.66 μm, Q = 1.09–1.13 (60/2).

Other specimens (paratypes) examined: China, Qinghai, Menyuan County, Xianmi Forest Farm, on the stump of *Juniperus*, 5 September 2018, *L.W. Zhou*, LWZ 20180905-6 (HMAS, paratype).

**Notes:** The thick-walled basidiospores make *Hyphodontia pachyspora* distinct in *Hyphodontia*. Microscopically, *H. pachyspora* resembles *H. arguta* by the absence of septocystidia. It differs from *H. arguta* by the thick-walled and wider basidiospores [19,20]. Three sequenced specimens of *H. arguta* clustered into two lineages in the ITS-based phylogeny (Figure 1): the first one including two specimens sequenced by Riebesehl et al. [34]; the second one including the specimen sequenced by Larsson et al. [7] and two specimens of *H. pachyspora*. The combined dataset of ITS, nLSU and mt-SSU regions also fully supported the second lineage (Figure 2). We did not examine the specimen sequenced by Larsson et al. [7], but the specimens of *H. pachyspora* clearly show morphological distinctions from *H. arguta* as mentioned above. Whether the two sequenced specimens by Riebesehl et al. [34] really represent *H. arguta* is still ambiguous, as discussed by Kan et al. [36].

**Figure 12:**
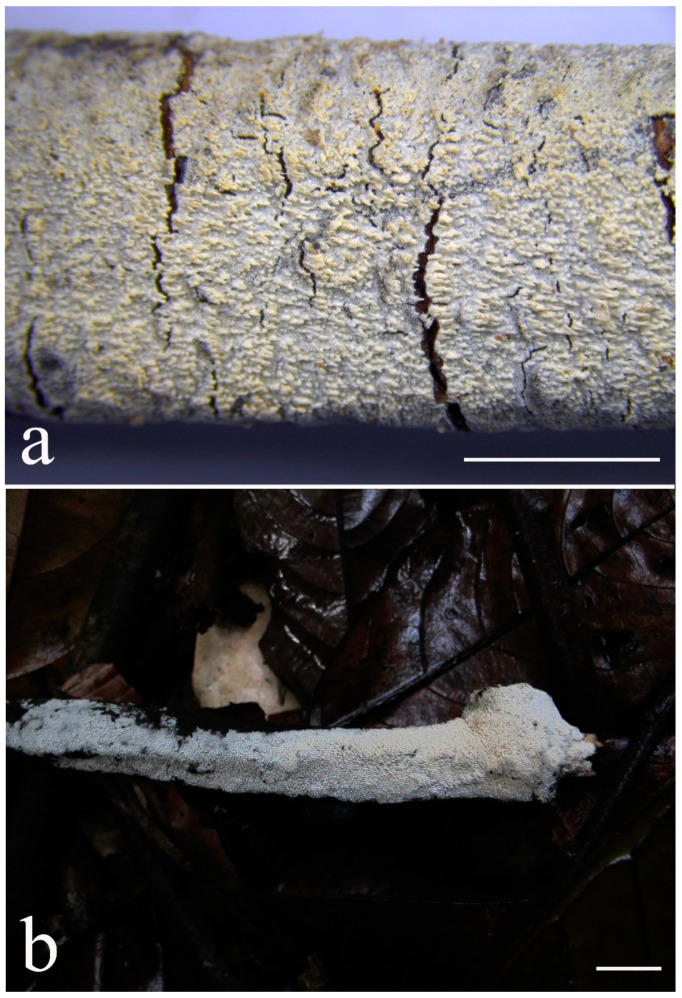
Basidiocarps of *Hyphodontia wongiae*. (**a**) LWZ 20180414-16 (holotype); (**b**) LWZ 20180417-8 (paratype).—Scale bars: **a** = 5 mm; **b** = 1 cm.

**Figure 13:**
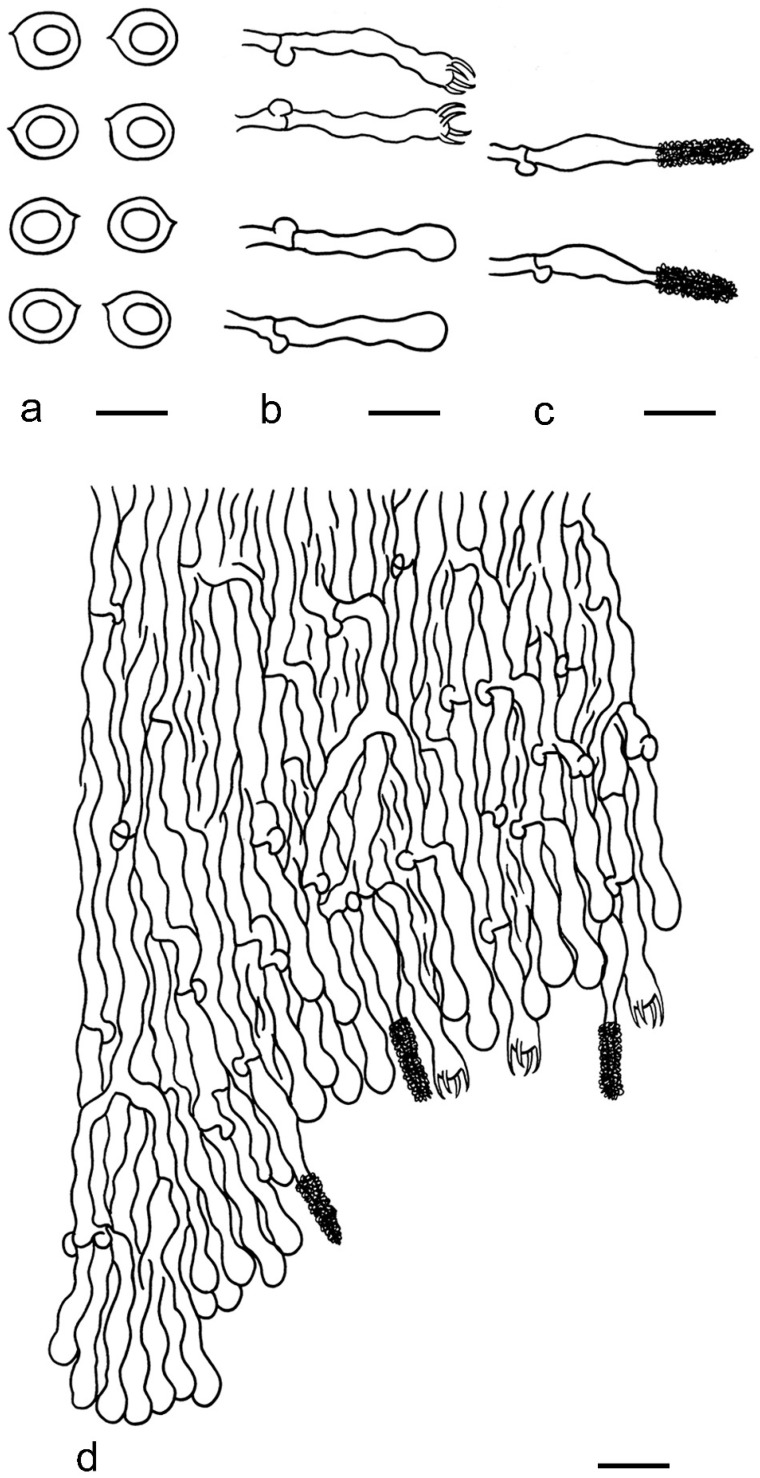
Microscopic structures of *Hyphodontia wongiae* (drawn from the holotype). (**a**) Basidiospores; (**b**) basidioles; (**c**) lagenocystidia; (**d**) a section of the basidiocarp.—Scale bars: **a** = 5 µm; **b**–**d** = 10 µm.

***Hyphodontia wongiae*** Xue W. Wang & L.W. Zhou, **sp. nov.** (Figure 12 and Figure 13)

Index Fungorum identifier: IF 557236

**Etymology:***Wongiae* (Latin), refers to the Malaysian biologist, Dr. Wei Chee Wong, who kindly arranged the author Li-Wei Zhou’s field trip in Malaysia.

**Type:** Malaysia, Kuala Lumpur, KL Forest Eco park, on fallen angiosperm twig, 14 Apr. 2018, *L.W. Zhou*, LWZ 20180414-16 (holotype HMAS).

**Description:** Basidiocarps annual, resupinate, adnate, cracked and brittle when dry. Hymenophore odontioid, white to cream. Margin paler than or concolorous with subiculum, abrupt. Hyphal system monomitic; generative hyphae with clamp connections, hyaline, thin-walled, dichotomous branching, tortuous, 1.5–3.5 μm in diam. Lagenocystidia abundant, thin-walled, with broad bases tapering abruptly towards the apices, apically encrusted. Basidia subclavate somewhat sinuous to utriform, 20–25 × 3.5–4.5 μm, with four sterigmata and a clamp connection at the base; basidioles similar in shape to basidia, but smaller. Basidiospores broadly ellipsoid or ovoid, with a large oily drop, hyaline, smooth, thin-walled, acyanophilous, inamyloid, indextrinoid, (3.5–)3.7–4.4(–4.5) × (3.1–)3.3–3.6(–3.8) μm, L = 4.03 μm, W = 3.44 μm, Q = 1.16–1.19 (60/2).

Other specimens (paratypes) examined: Malaysia, Selangor, Kota Damansara Community Forest Reserve, on fallen angiosperm twig, 17 Apr. 2018, *L.W. Zhou*, LWZ 20180417-8 (HMAS, paratype); on fallen angiosperm branch, 17 Apr. 2018, *L.W. Zhou*, LWZ 20180417-16 (HMAS, paratype).

**Notes:***Hyphodontia wongiae* resembles *H. wrightii* by sharing odontioid hymenophore and absence of capitate cystidia; however, *H. wongiae* differs in its broadly ellipsoid to ovoid basidiospores, which are shorter and wider than those of *H. wrightii* (4.5–5.5 × 2.5–3 µm) [19].

***Hyphodontia zhixiangii*** L.W. Zhou & Gafforov, in Kan, Gafforov, Li & Zhou, Phytotaxa 299(2): 275. 2017).

**Notes:***Hyphodontia zhixiangii* was originally described on the basis of Uzbek specimens [36]. Besides the holotype and two paratypes, the current phylogenetic analyses also recovered six Chinese specimens in the lineage of *H. zhixiangii* (Figure 1, Figure 2 and Figure 3). Within this lineage, three Uzbek specimens clustered together with strong support in the phylogenies based on three and six genes (Figure 2 and Figure 3). However, the monophyletic group of the six Chinese specimens was not supported. Therefore, these six specimens are confirmed to be *H. zhixiangii* from a phylogenetic perspective. This is the first record of *H. zhixiangii* from China.

The three original collections of *H. zhixiangii* inhabited *Juniperus* trees [36], while among the Chinese specimens, LWZ 20170818-13 grew on *Metasequoia glyptostroboides*, and LWZ 20180903-5, LWZ 20180903-9 and LWZ 20180904-12 grew on *Picea*. Contrary to these gymnosperm hosts, it is noteworthy that another two Chinese specimens LWZ 20170820-27 and LWZ 20170820-31 colonized unidentified angiosperm trees.
**A key to 13 accepted species of *Hyphodontia***1a. Hymenophore smooth to grandinioid.......................................................................................................................................2b. Hymenophore odontioid or poroid..............................................................................................................................................72a. Lagenocystidia abundant............................................................................................................................................................3b. Lagenocystidia rare or absent.......................................................................................................................................................53a. Capitate septocystidia absent; basidiospores thick-walled, more than 4 μm wide.........................................*H. pachyspora*b. Capitate septocystidia present; basidiospores thin-walled, less than 4 μm wide...................................................................44a. Hymenophore colliculose............................................................................................................................................*H. alutaria*b. Hymenophore grandinioid........................................................................................................................................*H. zhixiangii*5a. Basidiospores less than 5 μm long, less than 3 µm wide.........................................................................................*H. pallidula*b. Basidiospores more than 5 μm long, more than 4 µm wide......................................................................................................66a. Lagenocystidia generally rare...............................................................................................................................*H. subdetritica*b. Lagenocystidia absent.............................................................................................................................................*H. subpallidula*7a. Hymenophore poroid................................................................................................................................................*H. borbonica*b. Hymenophore odontioid...............................................................................................................................................................88a. Hymenophore brownish orange, cinnamon-buff, tawny olive or buckthorn brown.......................................*H. ochroflava*b. Hymenophore whitish to yellowish, cream or orange...............................................................................................................99a. Basidiospores globose, slightly thick-walled.....................................................................................................*H. sphaerospora*b. Basidiospores ellipsoid, cylindrical or ovoid, thin-walled......................................................................................................1010a. Capitate cystidia absent...........................................................................................................................................................11b. Capitate cystidia present..............................................................................................................................................................1211a. Basidiospores ellipsoid to cylindrical, more than 4.5 µm long, less than 3 µm wide..........................................*H. wrightii*b. Basidiospores broadly ellipsoid to ovoid, less than 4.5 µm long, more than 3 µm wide.......................................*H. wongiae*12a. Basidiospores more than 3 μm wide; aculei up to 2 mm long..................................................................................*H. arguta*b. Basidiospores less than 3 μm wide; aculei up to 6 mm long.....................................................................................*H. dhingrae*

***Schizoporaceae*** Jülich, Biblthca Mycol. 85: 389. 1982 (1981).

**Type genus:** *Schizopora* Velen., České Houby 4-5: 638. 1922.

= *Xylodon* (Pers.) Gray, Nat. Arr. Brit. Pl. (London) 1: 649. 1821.

**Description:** Basidiocarps resupinate or pileate. Hymenophore smooth, tuberculate, grandinioid, odontioid, coralloid, irpicoid or poroid; whitish to yellowish or creamish to greyish, buff to cinnamon or slightly ochraceous to ochraceous, beige or slightly orange, clay color or buckthorn brown. Hyphal system monomitic, pseudodimitic, dimitic or trimitic. Hyphae with clamp connections. Cystidia present or absent, different types: capitate to subcapitate, cylindrical to subcylindrical, fusiform, subulate, bladder-like, bottle-shaped, clavate, moniliform to submoniliform, pyriform, astro-, gloeo- or leptocystidia, lecythiform, rarely lagenocystidia and snake-like sinuous tramacystidia. Basidia barrel-shaped, clavate to subclavate, cylindrical to subcylindrical, pyriform, utriform, with two or four sterigmata. Basidiospores ellipsoid or subellipsoid, cylindrical to subcylindrical, ovoid, allantoid, globose or subglobose, smooth, thin- or thick-walled, hyaline, inamyloid, acyanophilous or slightly cyanophilous.

***Fasciodontia*** Yurchenko & Riebesehl, in Yurchenko, Riebesehl & Langer, Mycol. Progr. 19(2): 178. 2020.

**Type species:***Fasciodontia bugellensis* (Ces.) Yurchenko, Riebesehl & Langer, in Yurchenko, Riebesehl & Langer, Mycol. Progr. 19(2): 178. 2020.

See Yurchenko et al. [13] for description.

***Lyomyces*** P. Karst. [as *‘Lyomices’*], Revue mycol., Toulouse 3(no. 9): 23. 1881.

**Type species:** *Lyomyces serus* (Pers.) P. Karst., Revue mycol., Toulouse 3(no. 9): 23. 1881.

**Description:** Basidiocarps resupinate. Hymenophore smooth, grandinioid or odontioid; whitish to yellowish, greyish to creamish or pale beige. Hyphal system monomitic to pseudodimitic, usually with crystals. Hyphae with clamp connections. Leptocystidia of one or more types: capitate to subcapitate, cylindrical, fusiform or subulate. Basidia subcylindrical to cylindrical, utriform or clavate, mostly with four sterigmata. Basidiospores ellipsoid, cylindrical to subcylindrical, globose to subglobose, suballantoid or ovoid, smooth, thin- or slightly thick-walled, hyaline, inamyloid, acyanophilous or slightly cyanophilous.

***Lyomyces crustosus*** (Pers.) P. Karst., Revue mycol., Toulouse 3(no. 9): 23. 1881.

**Basionym:***Odontia crustosa* Pers., Observ. mycol. (Lipsiae) 2: 16. 1800 (1799).

**Notes:** In the ITS-based phylogeny, the strongly supported clade of *Lyomyces crustosus* included two well supported subclades (Figure 1). Compared with the ITS-based phylogeny (Figure 1), the phylogeny based on three and seven genes showed similar topologies, but the collections in each subclade were not totally consistent (LWZ 20170816-3; Figure 2 and Figure 4). *Lyomyces crustosus* has been recorded mostly on angiosperm hosts and occasionally on gymnosperm hosts, and vice versa for *L. juniperi* [80]. The newly studied Chinese specimens of *L. crustosus* were collected on both angiosperm and gymnosperm hosts, and they were separated according to their host types (Figure 2 and Figure 4). However, when collections from previous studies are taken into consideration, all collections of *L. crustosus* do not group strictly according to these host types (Figure 1 and Figure 2). Besides, the morphological characters within these collections are not distinct. Therefore, although the clade of *L. crustosus* includes collections with internal subclades, it has to be treated as a single species until the type or specimens of *L. crustosus* from the type locality are carefully studied. Noteworthily, collection Wu 0308-26 was previously labeled as *Lyomyces juniperi* and was collected on an angiosperm host [80], but it fell within the clade of *L. crustosus* and was more closely related to the collections on gymnosperm hosts in the ITS-based phylogeny (Figure 1). Therefore, it is considered to be *L. crustosus*.

**Figure 14:**
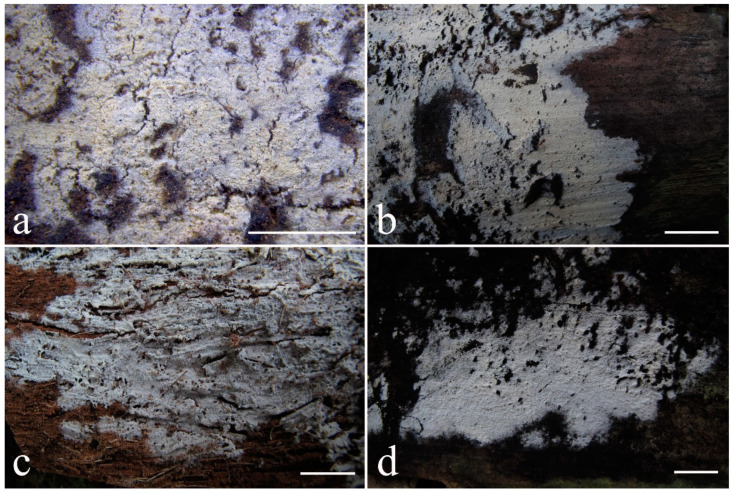
Basidiocarps of *Lyomyces elaeidicola*. (**a**,**b**) LWZ 20180411-20 (holotype); (**c**) LWZ 20180411-17 (paratype); (**d**) LWZ 20180411-19 (paratype).—Scale bars: **a** = 5 mm; **b**–**d** = 1 cm.

**Figure 15:**
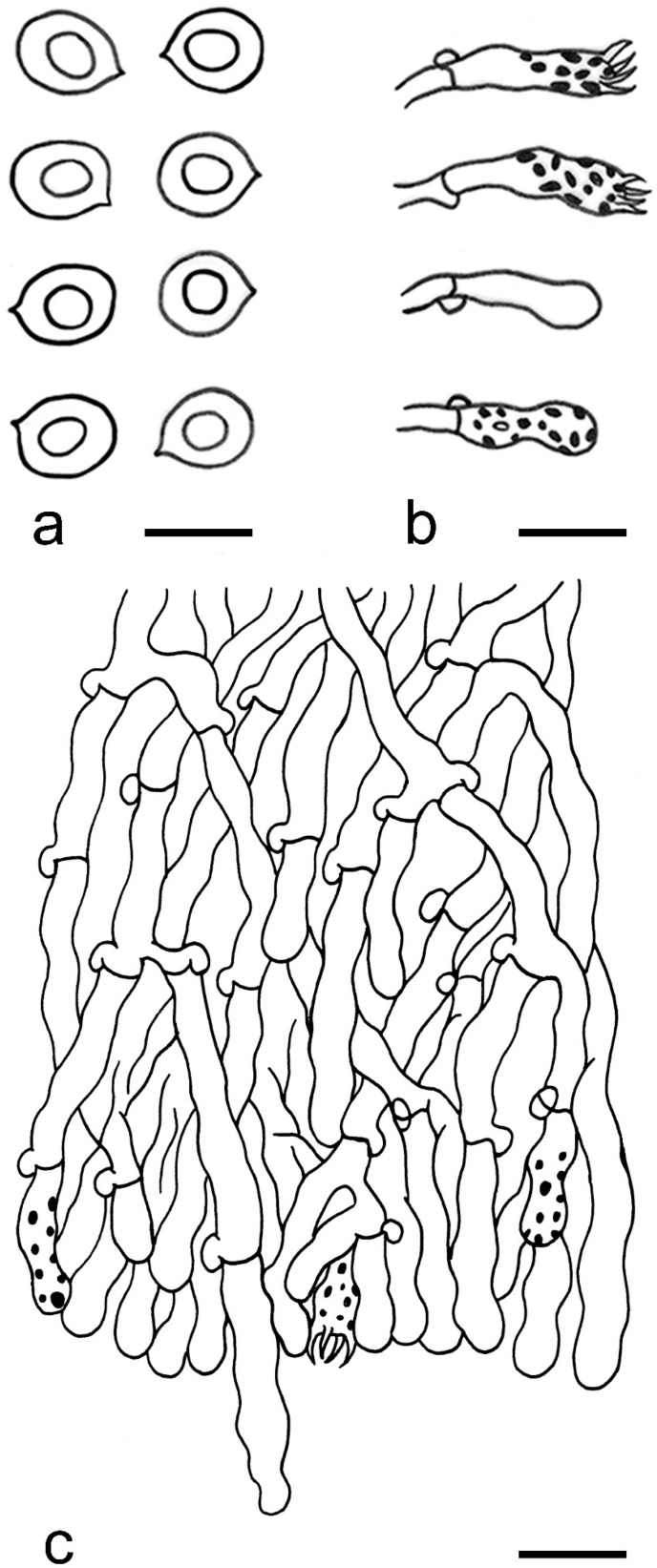
Microscopic structures of *Lyomyces elaeidicola* (drawn from the holotype). (**a**) Basidiospores; (**b**) basidia and basidioles; (**c**) a section of the basidiocarp.—Scale bars: **a** = 5 µm; **b**,**c** = 10 µm.

***Lyomyces elaeidicola*** Xue W. Wang & L.W. Zhou, **sp. nov.** (Figure 14 and Figure 15)

**Index Fungorum identifier:** IF 557238

**Etymology:***Elaeidicola* (Latin), refers to the host tree *Elaeis guineensis*.

**Type:** Malaysia, Selangor, Sungai Gapi, on the living tree of *Elaeis guineensis*, 11 April 2018, *L.W. Zhou*, LWZ 20180411-20 (holotype HMAS).

**Description:** Basidiocarps annual, resupinate, adnate, cracked and brittle when dry. Hymenophore smooth to slightly tuberculate, limestone-white to cream. Margin paler than or concolorous with subiculum, thinning. Hyphal system monomitic; generative hyphae with clamp connections, hyaline, thin-walled, dichotomous branching, tortuous, 3–5.5 μm in diam. Cystidia absent; capitate hyphal ends rarely present, originating from hymenia or subhymenia. Basidia subclavate or utriform, 15–25 × 4–5 μm, usually covered with crystals, with four sterigmata and a clamp connection at the base; basidioles similar in shape to basidia, but smaller. Basidiospores broadly ellipsoid, with a large oily drop, hyaline, smooth, thin-walled, cyanophilous, inamyloid, indextrinoid, (4.2–)4.3–4.7(–4.8) × (3.1–)3.2–3.8(–3.9) μm, L = 4.49 μm, W = 3.46 μm, Q = 1.18–1.30 (120/4).

Other specimens (paratypes) examined: Malaysia, Selangor, Changkat Asa, on the fallen branch of *Elaeis guineensis*, 11 April 2018, *L.W. Zhou*, LWZ 20180411-2 (HMAS, paratype); Selangor, Sungai Gapi, on the fallen branch of *E. guineensis*, 11 April 2018, LWZ 20180411-17 (HMAS, paratype); on the living tree of *E. guineensis*, 11 April 2018, LWZ 20180411-19 (HMAS, paratype).

**Notes:** *Lyomyces elaeidicola* is similar to *L. mascarensis* by the presence of capitate cystidia and cyanophilous basidiospores both similar in size; however, *L. mascarensis* differs by the presence of submoniliform and tapering cystidia [17]. Moreover, the growth on *Elaeis guineensis* makes *L. elaeidicola* easily distinguished in the field.

**Figure 16:**
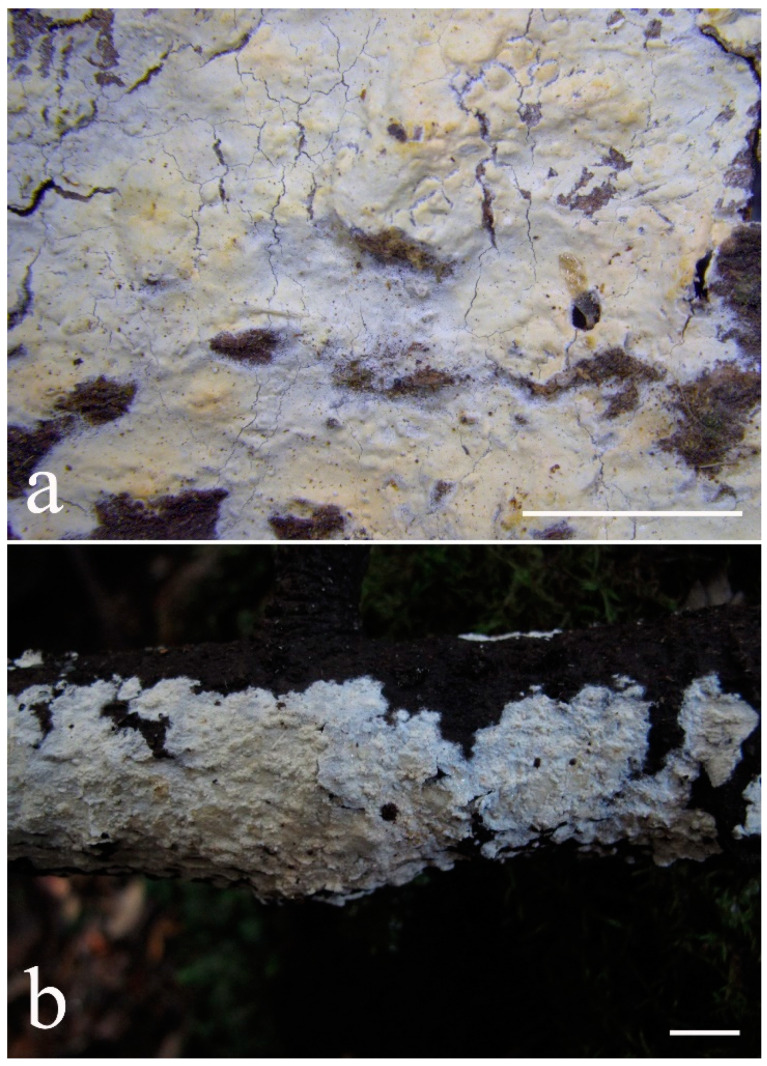
Basidiocarps of *Lyomyces gatesiae* (LWZ 20180515-3, holotype).—Scale bars: **a** = 5 mm; **b** = 1 cm.

**Figure 17:**
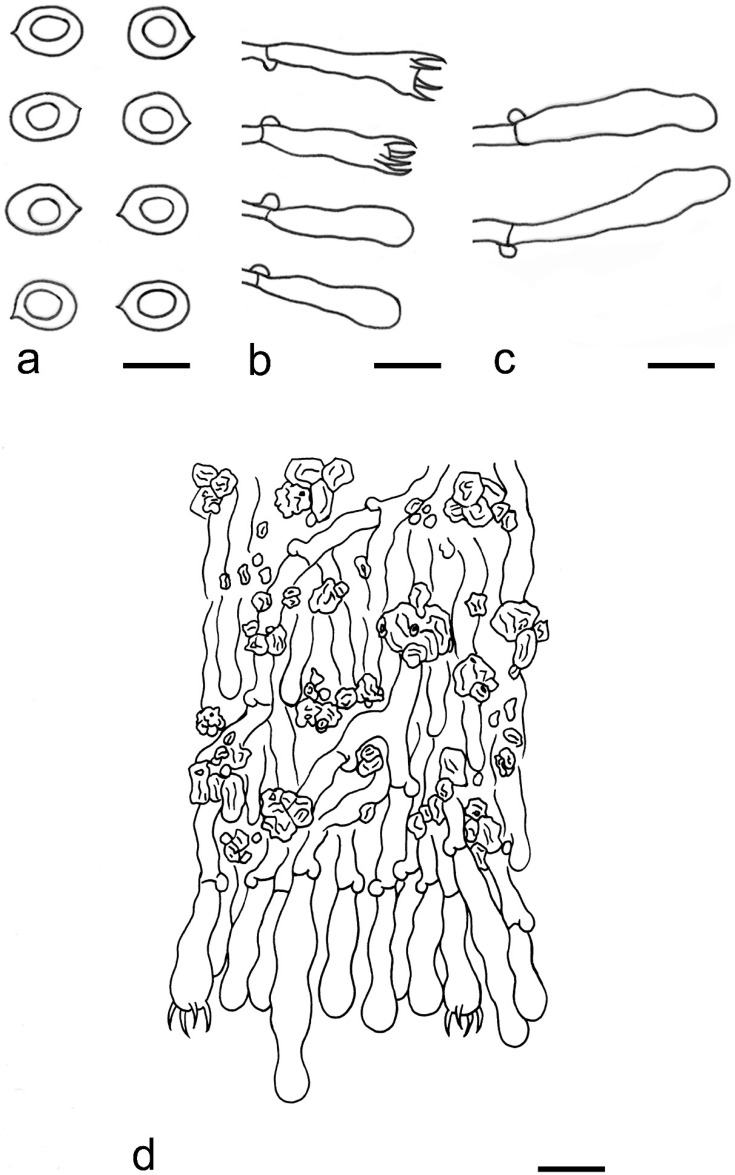
Microscopic structures of *Lyomyces gatesiae* (drawn from the holotype). (**a**) Basidiospores; (**b**) basidia and basidioles; (**c**) cylindrical, subcapitate cystidia; (**d**) a section of the basidiocarp.—Scale bars: **a** = 5 µm; **b**–**d** = 10 µm.

***Lyomyces gatesiae*** Xue W. Wang & L.W. Zhou, **sp. nov.** (Figure 16 and Figure 17)

**Index Fungorum identifier:** IF 557241

**Etymology:***Gatesiae* (Latin), refers to the Australian mycologist, Dr. Genevieve Gates, who kindly arranged the author Li-Wei Zhou’s field trip in Tasmania, Australia.

**Type:** Australia, Tasmania, Tahune Adventures, The Look-in Look-out, on fallen angiosperm branch, 15 May 2018, *L.W. Zhou*, LWZ 20180515-3 (holotype MEL, isotype HMAS).

**Description:** Basidiocarps annual, resupinate, adnate, cracked and brittle when dry. Hymenophore smooth or slightly warted, white to cream. Margin paler than or concolorous with subiculum, abrupt. Hyphal system monomitic; generative hyphae with clamp connections, hyaline, thin-walled, dichotomous branching, tortuous, frequently encrusted with granular crystals, 2.5–4.5 μm in diam. Cystidia cylindrical or slightly subcapitate, thin-walled, 30–35 × 5–7 μm. Basidia clavate or subclavate, 20–25 × 4.5–5.5 μm, with four sterigmata and a clamp connection at the base; basidioles similar in shape to basidia, but smaller. Basidiospores broadly ellipsoid or ovoid, with a large oily drop, hyaline, smooth, thin-walled, acyanophilous, inamyloid, indextrinoid, (4.1–)4.2–5.1(–5.2) × (3.1–)3.2–4.7(–4.8) μm, L = 4.63 μm, W = 3.73 μm, Q = 1.17–1.30 (60/2). 

Other specimens (paratypes) examined: Australia, Tasmania, Tahune Adventures, Arve River Picnic Area, on fallen angiosperm twig, 15 May 2018, *L.W. Zhou*, LWZ 20180515-32 (HMAS, paratype).

**Notes:***Lyomyces gatesiae* resembles *L. orientalis* by smooth to slightly warted hymenophore, abrupt margin, and the presence of subcapitate cystidia, but the latter species has smaller cystidia (13–20 × 3–5 μm) and longer basidiospores (5–6 µm in length) [17]. 

***Lyomyces juniperi*** (Bourdot & Galzin) Riebesehl & Langer, Mycol. Progr. 16(6): 647. 2017.

**Basionym:***Corticium serum* var. *juniperi* Bourdot & Galzin, Bull. Soc. mycol. Fr. 27(2): 246. 1911.

**Notes:** Three collections previously labeled as *Lyomyces juniperi* were scattered in three separated lineages in the ITS-based phylogeny: GEL 4940 and Wu 0910-95 were independent, while Wu 0308-26 merged in the clade of *Lyomyces crustosus* (Figure 1). Morphological and ecological differences between *L. crustosus* and *L. juniperi* were discussed in detail by Yurchenko et al. [80]. However, the overlap of morphological and ecological characters makes the differences not straightforward. Therefore, the collection Wu 0308-26 is tentatively considered to be *L. crustosus* following the phylogenetic evidence (Figure 1). As collections GEL 4940 and Wu 0910-95 were both collected far away from the type locality in France [80], it is unclear whether either of them can represent *L. juniperi*. The type of specimens from the type locality of *L. juniperi* need to be sequenced to solve this issue. Until then, we tentatively label both GEL 4940 and Wu 0910-95 as *L. juniperi*.

**Figure 18:**
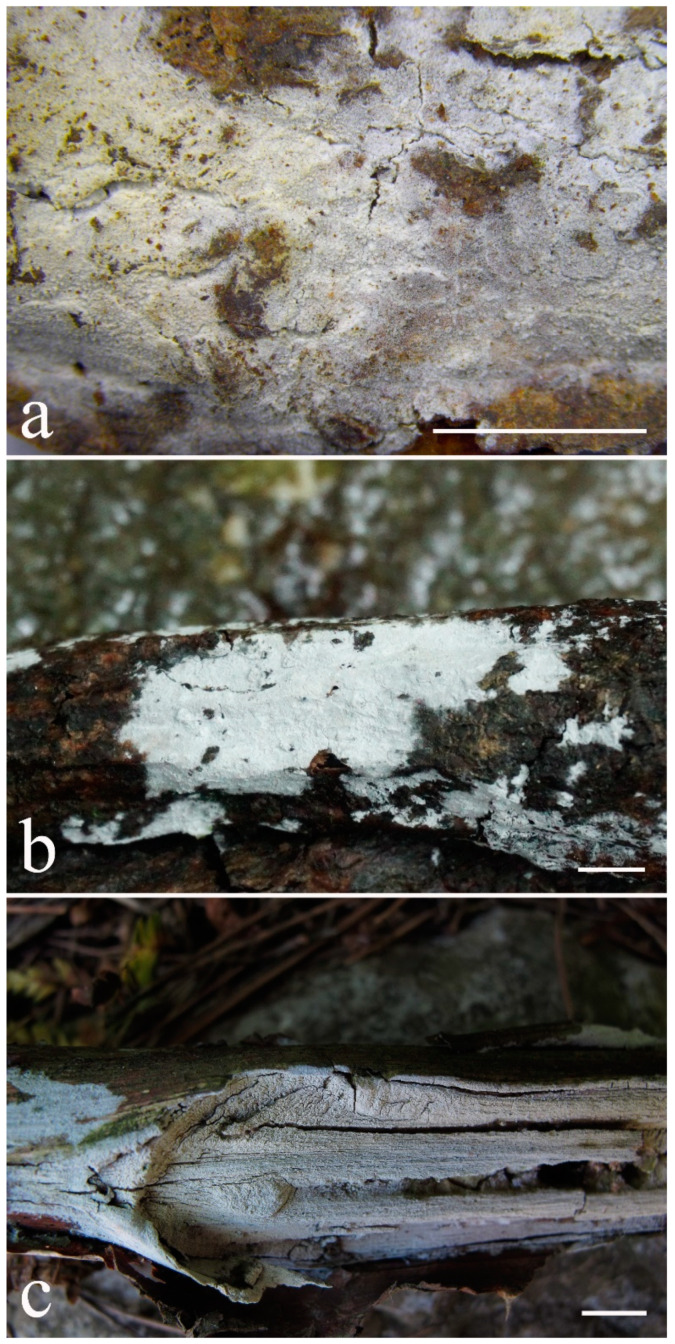
Basidiocarps of *Lyomyces leptocystidiatus*. (**a**,**b**) LWZ 20170814-14 (holotype); (**c**) LWZ 20170818-9 (paratype).—Scale bars: **a** = 5 mm; **b**,**c** = 1 cm.

**Figure 19:**
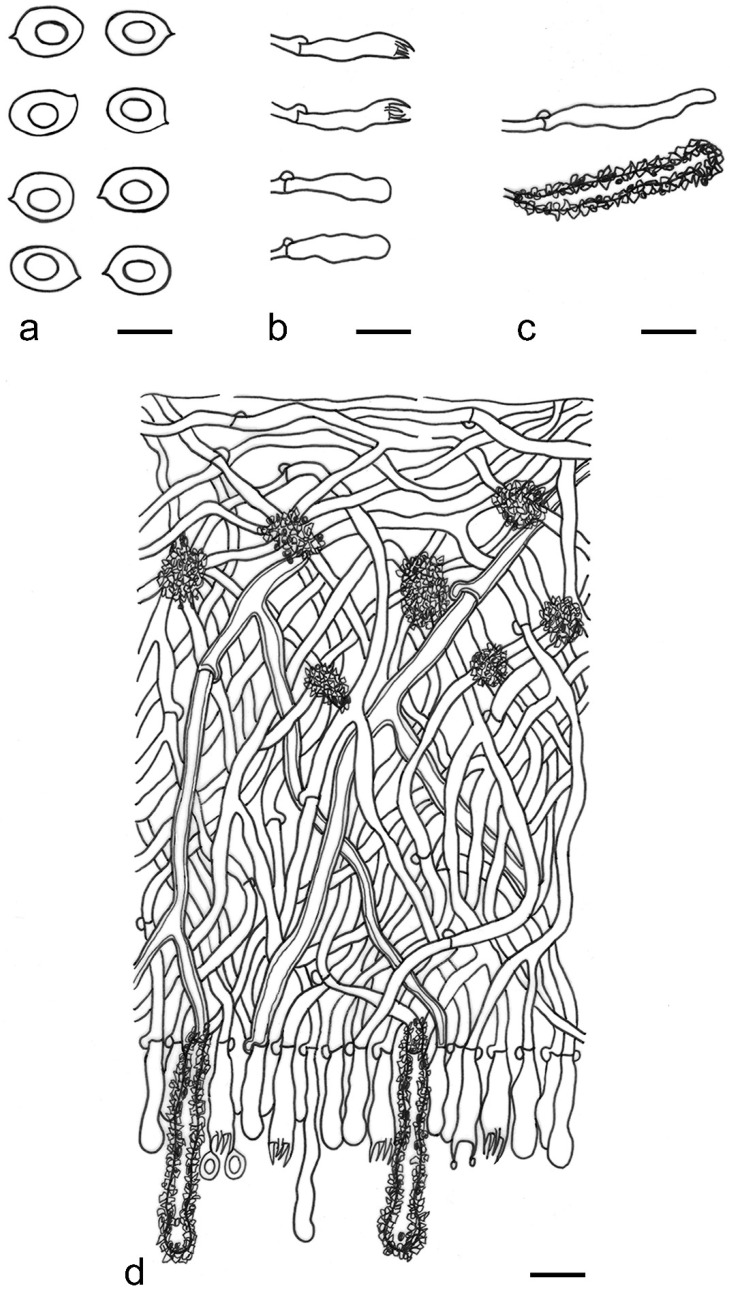
Microscopic structures of *Lyomyces leptocystidiatus* (drawn from the holotype). (**a**) Basidiospores; (**b**) basidia and basidioles; (**c**) leptocystidia; (**d**) a section of the basidiocarp.—Scale bars: **a** = 5 µm; **b**–**d** = 10 µm.

***Lyomyces leptocystidiatus*** Xue W. Wang & L.W. Zhou, **sp. nov.** (Figure 18 and Figure 19)

**Index Fungorum identifier:** IF 557621

**Etymology:***Leptocystidiatus* (Latin), refers to leptocystidia.

**Type:** China, Hubei, Wufeng County, Chaibuxi Grand Canyon Scenic Spot, on fallen angiosperm branch, 14 Aug. 2017, *L.W. Zhou*, LWZ 20170814-14 (holotype IFP, isotype HMAS).

**Description:** Basidiocarps annual, resupinate, adnate, cracked and brittle when dry. Hymenophore white to cream, smooth or slightly tuberculate; margin paler, thinning, pruinose. Hyphal system monomitic; generative hyphae with clamp connections, hyaline, thin- to slightly thick-walled, dichotomous branching, tortuous, frequently encrusted with crystals, 2.5–5.5 μm in diam. Leptocystidia numerous, usually encrusted with crystals, thin- or slightly thick-walled, 25–45 × 3.5–5.5 μm. Basidia utriform or clavate, 15–25 × 3.5–5.5 μm, with four sterigmata and a clamp connection at the base; basidioles similar in shape to basidia, but smaller. Basidiospores broadly ellipsoid, usually with a large oily drop, hyaline, smooth, thin-walled, acyanophilous, inamyloid, indextrinoid, (4.9–)5.0–6.1(–6.2) × (2.8–)2.9–4.3(–4.4) μm, L = 5.34 μm, W = 3.64 μm, Q = 1.39–1.52 (90/3).

Other specimens (paratypes) examined: China, Hubei, Wufeng County, Chaibuxi Grand Canyon Scenic Spot, on fallen angiosperm twig, 15 August 2017, *L.W. Zhou*, LWZ 20170815-43 (IFP, paratype); Hubei, Enshi, Enshi Great Canyon, on dead standing angiosperm, 18 Aug. 2017, *L.W. Zhou*, LWZ 20170818-9 (IFP, paratype).

**Notes:** The regular presence of leptocystidia encrusted with crystals make *Lyomyces leptocystidiatus* distinct and reminiscent of *L. boninensis. Lyomyces boninensis* differs in its smooth, thin-walled, slightly fusiform and wider leptocystidia (5–7 µm in width), and slightly narrower hyphae (up to 4 µm) [14,16,31].

***Lyomyces sambuci*** (Pers.) P. Karst., Bidr. Känn. Finl. Nat. Folk 37: 153. 1882.

**Basionym:***Corticium sambuci* Pers., Neues Mag. Bot. 1: 111. 1794.

= *Lyomyces serus* (Pers.) P. Karst., Revue mycol., Toulouse 3(no. 9): 23. 1881.

**Notes:***Lyomyces sambuci* is a worldwide species and has priority over the synonym *L. serus*, the generic type of *Lyomyces* [81]. However, its wide distribution is questioned, because *L. sambuci* has been considered to be a species complex in recent decades [20,30]. Recently, four new species were segregated from this complex [17]. Nevertheless, the reduced concept of *L. sambuci* is still a species complex, because two distinct lineages of *L. sambuci* with similar morphological characters were present in an ITS-based phylogeny [17]. For this reason, the lineage composed of the collections of 170SAMHYP and GEL 2414 was treated as an undescribed cryptic species, while the other lineage was accepted to be the true *L. sambuci* [17]. The current ITS-based phylogeny (Figure 1) confirmed these two lineages and also revealed an additional lineage with two collections labeled as *L*. cf. *sambuci*. This *L*. cf. *sambuci* lineage quite possibly represents a new species; however, we did not have the opportunity to examine specimens. 

Nine additional Chinese specimens of *L. sambuci* were newly studied and they all merged together with the clade of *L. sambuci* (in the strict sense) in the ITS-based phylogeny (Figure 1). Among the nine specimens, LWZ 20170908-13 was collected from Beijing, China on an angiosperm host that is the common host type for this species, whereas another eight specimens were all collected from Gansu Province, China on *Juniperus*. The ITS-based phylogeny (Figure 1) did not discriminate these nine specimens well, but the phylogenies based on three genes (Figure 2) and seven genes (Figure 4) both strongly supported the separation between LWZ 20170908-13 and the other eight specimens. A multilocus-based phylogeny that includes additional collections of *L. sambuci* on angiosperm hosts will be helpful to provide more reliable evidence to judge whether collections on different host tree types represent two independent species. For now, we still treat the specimens on *Juniperus* as *L. sambuci*.
**A key to 28 accepted species of *Lyomyces***1a. Hymenophore verrucose, grandinioid or odontioid................................................................................................................2b. Hymenophore smooth or slightly grandinioid.........................................................................................................................132a. Subulate cystidia present, sometimes apically with a small capitate structure.....................................................................3b. Subulate cystidia absent.................................................................................................................................................................73a. Capitate cystidia present..........................................................................................................................................*L. macrescens*b. Capitate cystidia absent, very few subcapitate cystidioles possible.........................................................................................44a. Subulate cystidioles only laterally in aculei..............................................................................................................*L. stratosus*b. Subulate cystidia in hymenium....................................................................................................................................................55a. Subulate cystidia apically not capitate........................................................................................................................*L. juniperi*b. Subulate cystidia sometimes apically capitate............................................................................................................................66a. Hymenophore young grandinioid, odontioid when older, white to yellowish or cream; basidiospores narrowly ellipsoid to cylindrical, sometimes slightly allantoid...........................................................................................*L. crustosus*b. Hymenophore partly fine aculeate, white to pale greyish; basidiospores cylindrical....................................*L. vietnamensis*7a. With septocystidia, capitate cystidia and transitional forms........................................................................................*L. albus*b. Without septocystidia....................................................................................................................................................................88a. Basidiospores globose, 4–5 μm in diam, verrucose, slightly thick-walled..........................................................*L. griseliniae*b. Basidiospores ellipsoid, smooth, thin-walled.............................................................................................................................99a. Basidiospores less than 3.5 μm wide........................................................................................................................................10b. Basidiospores more than 3.5 μm wide.......................................................................................................................................1110a. Basidiospores less than 5.5 µm long; capitate cystidia present...............................................................*L. microfasciculatus*b. Basidiospores mostly more than 5.5 µm long; capitate cystidia absent...................................................................*L. stratosus*11a. Hymenophore odontioid, margin abrupt..............................................................................................................................12b. Hymenophore grandinioid, margin thinning out......................................................................................*L. capitatocystidiatus*12a. Hyphae with a lot of crystals...................................................................................................................................*L. fimbriatus*b. Hyphae without crystals.....................................................................................................................................................*L. pruni*13a. Basidiospores mostly more than 5.5 μm wide....................................................................................................*L. incrustatus*b. Basidiospores less than 5.5 μm wide..........................................................................................................................................1414a. Basidiospores suballantoid to reniform..................................................................................................................*L. eburneus*b. Basidiospores not reniform.........................................................................................................................................................1515a. Generally with two or rarely three sterigmata................................................................................................*L. bisterigmatus*b. Generally with four sterigmata...................................................................................................................................................1616a. Hymenophore margin abrupt.................................................................................................................................................17b. Hymenophore margin thinning out...........................................................................................................................................2417a. Capitate or subcapitate cystidia present................................................................................................................................18b. Capitate or subcapitate cystidia absent......................................................................................................................................2318a. Basidiospores cyanophilous....................................................................................................................................................19b. Basidiospores acyanophilous......................................................................................................................................................2219a. Submoniliform cystidia present..........................................................................................................................*L. mascarensis*b. Submoniliform cystidia absent, tapering cystidia present..................................................................................................2020a. Tapering cystidia more than 40 μm long............................................................................................................*L. bambusinus*b. Tapering cystidia less than 40 μm long..................................................................................................................................2121a. Fusoid cystidioles present...........................................................................................................................*L. wuliangshanensis*b. Fusoid cystidioles absent............................................................................................................................................*L. cremeus*22a. Basidiospores mostly less than 5 μm long.................................................................................................................*L. gatesiae*b. Basidiospores more than 5 μm long............................................................................................................................*L. orientalis*23a. Fusiform or cylindrical hyphidia-like cystidia present.......................................................................................*L. organensis*b. Subulate cystidia present................................................................................................................................................*L. juniperi*24a. Basidiospores mostly more than 6.5 µm wide..................................................................................................*L. macrosporus*b. Basidiospores less than 6.5 µm wide..........................................................................................................................................2525a. Capitate cystidia absent...........................................................................................................................................................26b. Capitate cystidia present..............................................................................................................................................................2826a. Basidiospores more than 7 μm long..................................................................................................................*L. allantosporus*b. Basidiospores less than 7 μm long..............................................................................................................................................2727a. Basidiospores cyanophilous...................................................................................................................................*L. elaeidicola*b. Basidiospores acyanophilous......................................................................................................................................................2928a. Hymenophore smooth; capitate cystidia; hyphae usually with a lot of crystals..................................................*L. sambuci*b. Hymenophore first for a long time porose-reticulate, later smooth; hyphae usually without crystals...................*L. erastii*29a. Leptocystidia present...............................................................................................................................................................30b. Leptocystidia absent.....................................................................................................................................................................3130a. Leptocystidia slightly fusiform, not encrusted, thin-walled, more than 5 µm wide; hyphae up to 4 µm in diam...........................................................................................................................................................................*L. boninensis*b. Leptocystidia not fusiform, encrusted with crystals, thin- to slightly thick-walled, mostly less than 5 µm wide; hyphae up to 5.5 µm in diam........................................................................................................................................*L. leptocystidiatus*31a. Hymenophore very finely aculeate...................................................................................................................*L. vietnamensis*b. Hymenophore fairly smooth...................................................................................................................................*L. tenuissimus*

***Xylodon*** (Pers.) Gray, Nat. Arr. Brit. Pl. (London) 1: 649. 1821.

= *Schizopora* Velen., České Houby 4-5: 638. 1922.

= *Lagarobasidium* Jülich, Persoonia 8(1): 84. 1974.

= *Palifer* Stalpers & P.K. Buchanan, N.Z. Jl Bot. 29(3): 339. 1991.

**Type species:** *Xylodon quercinus* (Pers.) Gray, Nat. Arr. Brit. Pl. (London) 1: 649. 1821.

**Description:** Basidiocarps resupinate or pileate. Hymenophore smooth, tuberculate, grandinioid, odontioid, coralloid, irpicoid or poroid; whitish to yellowish or creamish, buff to cinnamon or slightly ochraceous to ochraceous, beige or slightly orange, clay color or buckthorn brown. Hyphal system monomitic, pseudodimitic, dimitic or trimitic. Hyphae with clamp connections. Cystidia absent or present, different types: bladder-like, bottle-shaped, capitate, clavate, cylindrical, fusiform, lecythiform, moniliform, pyriform, subulate, astro-, gloeo- or leptocystidia, rarely lagenocystidia and snake-like sinuous tramacystidia. Basidia barrel-shaped, clavate, cylindrical to subcylindrical, pyriform, sinuous or utriform, with two or four sterigmata. Basidiospores ellipsoid, cylindrical, ovoid, allantoid or subglobose, smooth, thin- or thick-walled, hyaline, inamyloid, acyanophilous or slightly cyanophilous.

**Figure 20:**
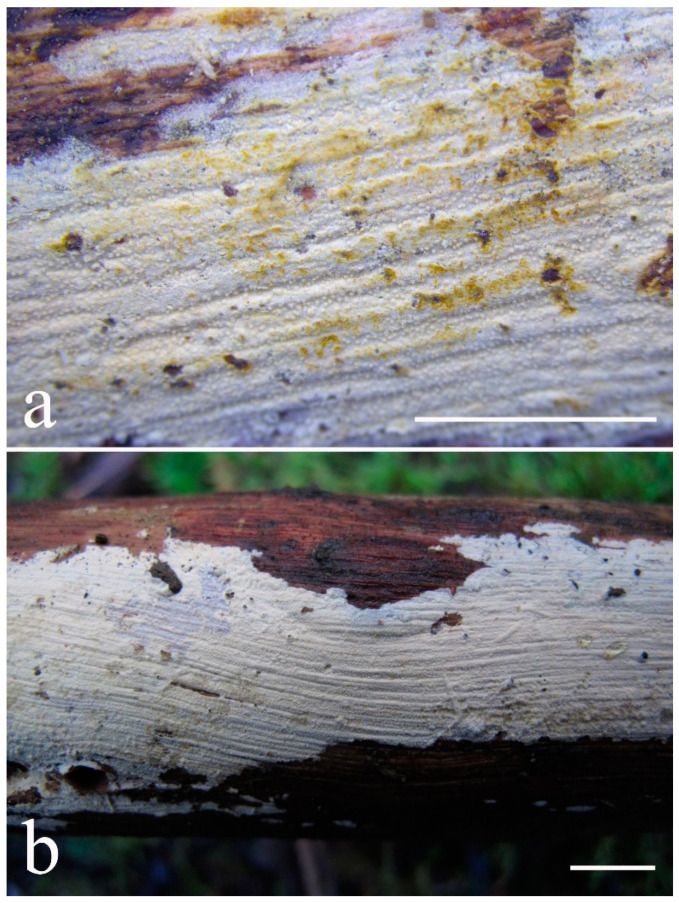
Basidiocarps of *Xylodon acystidiatus* (LWZ 20180514-9, holotype).—Scale bars: **a** = 5 mm; **b** = 1 cm.

**Figure 21:**
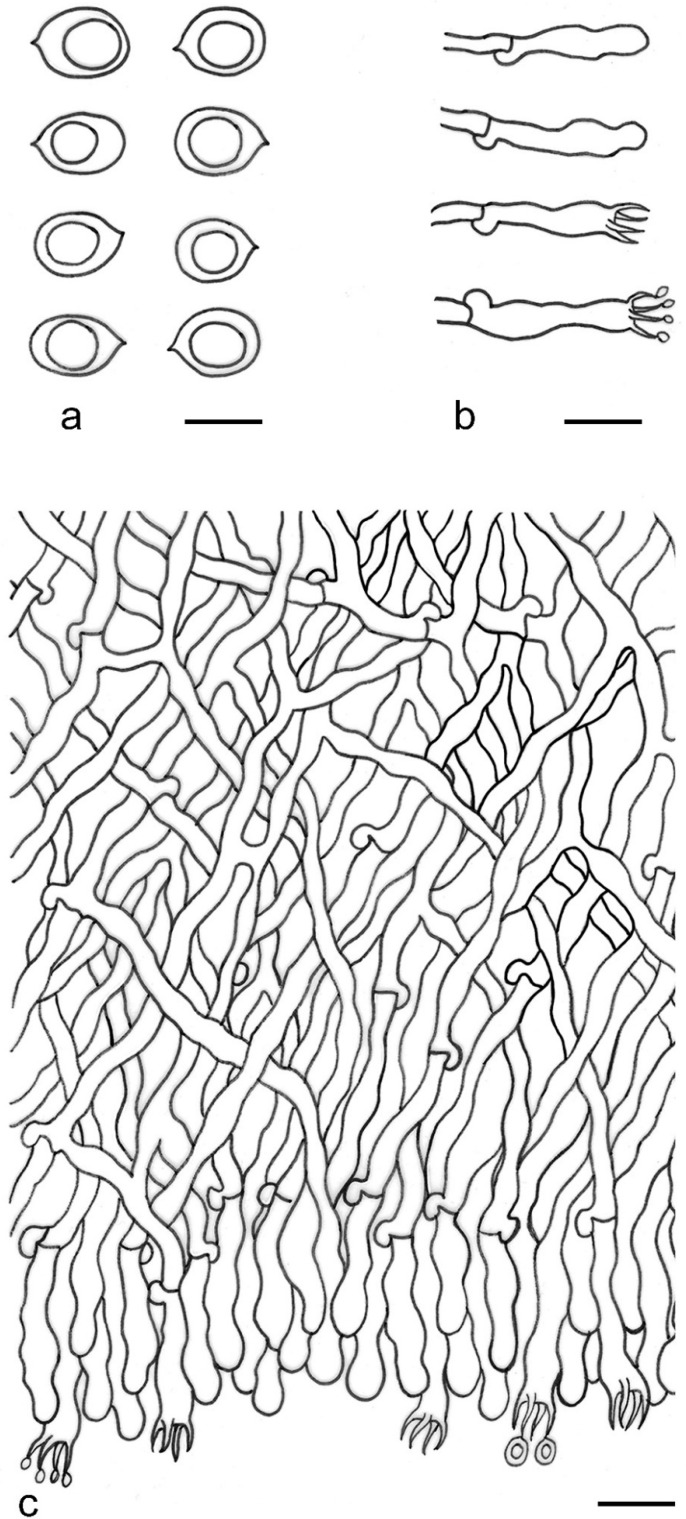
Microscopic structures of *Xylodon acystidiatus* (drawn from the holotype). (**a**) Basidiospores; (**b**) basidia and basidioles; (**c**) a section of the basidiocarp.—Scale bars: **a** = 5 µm; **b**,**c** = 10 µm.

***Xylodon acystidiatus*** Xue W. Wang & L.W. Zhou, **sp. nov.** (Figure 20 and Figure 21)

**Index Fungorum identifier:** IF 557736

**Etymology:***Acystidiatus* (latin), refers to the absence of cystidia.

**Type:** Australia, Tasmania, Timbs Track, on fallen angiosperm branch, 14 May 2018, *L.W. Zhou*, LWZ 20180514-9 (holotype MEL, isotype HMAS).

**Description:** Basidiocarps annual, resupinate, adnate, cracked and brittle when dry. Hymenophore smooth or slightly warted, white to buff. Margin paler than or concolorous with subiculum, abrupt, pruinose. Hyphal system monomitic; generative hyphae with clamp connections, hyaline, thin-walled, dichotomous branching, tortuous, 2.5–4.5 μm in diam. Cystidia absent. Basidia subclavate or subcapitate, 20–25 × 4–4.5 μm, with four sterigmata and a clamp connection at the base; basidioles similar in shape to basidia, but smaller. Basidiospores ellipsoid or ovoid, with a large oily drop, hyaline, smooth, thin-walled, acyanophilous, inamyloid, indextrinoid, (4.6–)4.7–5.3(–5.4) × (2.6–)2.7–3.7(–3.8) μm, L = 5.01 μm, W = 3.30 μm, Q = 1.49–1.54 (60/2).

Other specimens (paratypes) examined: Australia, Tasmania, Tahune Adventures, Keogh’s Creek Walk, on fallen angiosperm twig, 15 May 2018, *L.W. Zhou*, LWZ 20180515-35 (HMAS, paratype).

**Notes:** The absence of cystidia makes *Xylodon acystidiatus* distinct from most of the species belonging to *Hyphodontia* sensu lato. *Xylodon papillosus* and *X. rudis* also lack cystidia and have basidiospores that are similar in size (4.5–5 × 2.8–3.5 µm and 5 × 4 µm, respectively), but these two species differ in the presence of cylindrical cystidioles and capitate hyphal endings, respectively [16]. Moreover, *X. rudis* also differs from *X. acystidiatus* in its odontioid hymenophore [16]. *Xylodon subglobosus* is another species of *Xylodon* without cystidia; however, it differs from *X. acystidiatus* in its odontioid hymenophore, and thick-walled and encrusted hyphae [82].

***Xylodon apacheriensis*** (Gilb. & Canf.) Hjortstam & Ryvarden, Syn. Fung. (Oslo) 26: 34. 2009.

**Basionym:** *Poria apacheriensis* Gilb. & Canf., Mycologia 65(5): 1117. 1973.

**Notes:** Two collections previously labeled as *Xylodon apacheriensis* [15,16] were separated in the current phylogeny (Figure 1). The collection Canfield 180 is the holotype and of course represents this species [16], whereas the collection Wu 0910-58 merged into the clade of *X. niemelaei* and is accepted to be that species.

**Figure 22:**
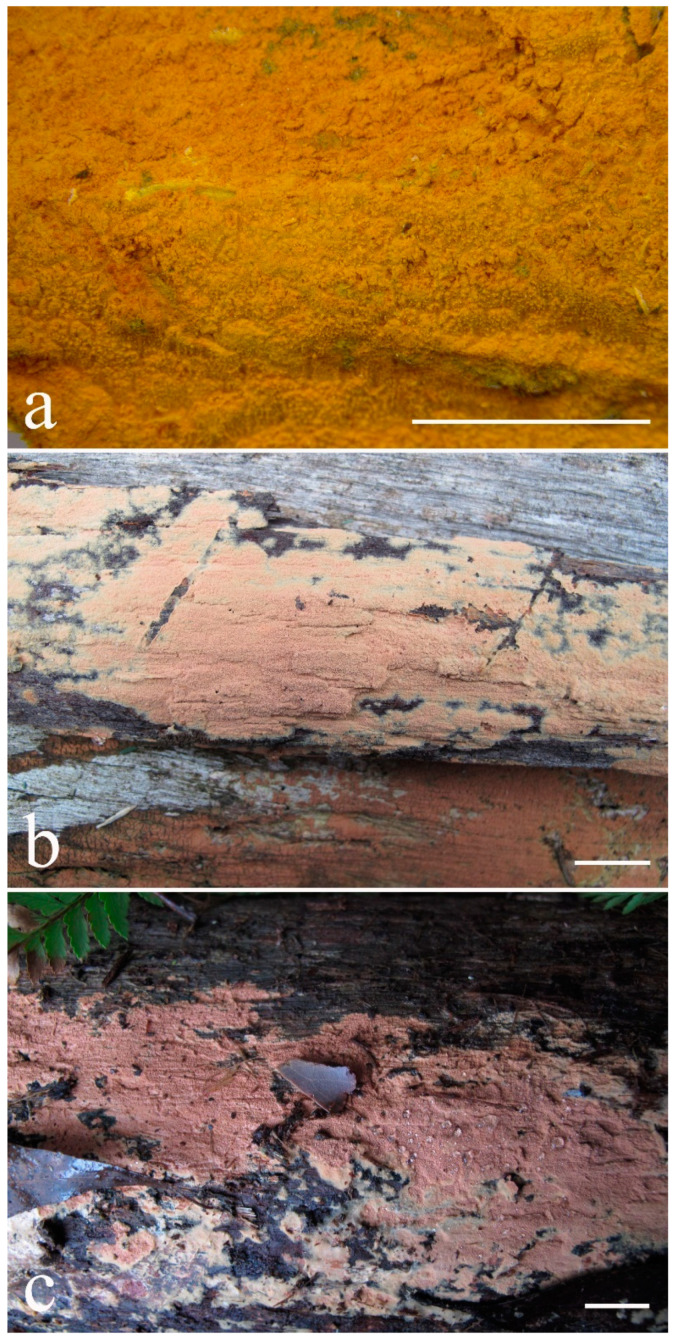
Basidiocarps of *Xylodon australis*. (**a**) LWZ 20180513-6 (epitype); (**b**) LWZ 20180509-15; (**c**) LWZ 20180512-14.—Scale bars: **a** = 5 mm; **b**,**c** = 1 cm.

**Figure 23:**
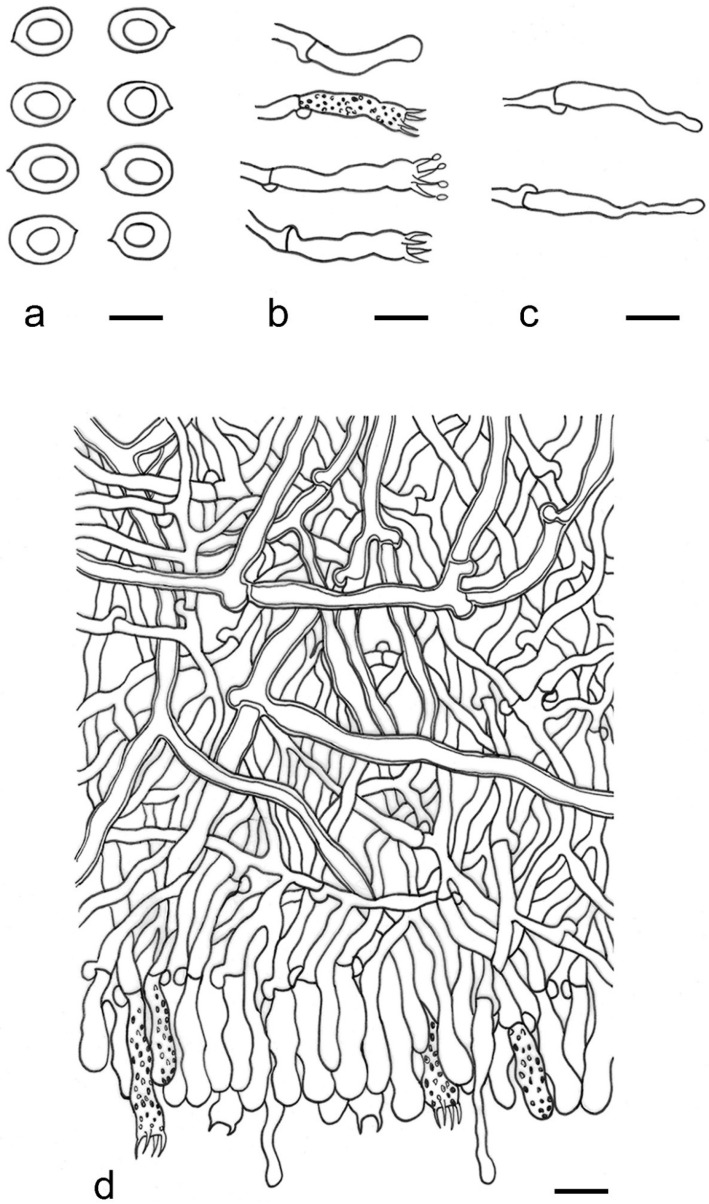
Microscopic structures of *Xylodon australis* (drawn from the epitype). (**a**) Basidiospores; (**b**) basidia and basidioles; (**c**) subulate cystidia; (**d**) a section of the basidiocarp.—Scale bars: **a** = 5 µm; **b**–**d** = 10 µm.

***Xylodon australis*** (Berk.) Hjortstam & Ryvarden, Syn. Fung. (Oslo) 23: 98. 2007. (Figure 22 and Figure 23)

**MycoBank:** MB505165

**Basionym:***Grandinia australis* Berk., in Hooker, Bot. Antarct. Voy., III, Fl. Tasman. 2: 257. 1859 (1860).

= *Hyphodontia australis* (Berk.) Hjortstam, Mycotaxon 54: 187. 1995.

**Holotype:** Australia, Tasmania, on dead wood, Archer (K(M) 56442). 

**Epitype (designated here):** Australia, Tasmania, Hobart, Mount Wellington, on fallen angiosperm trunk, 13 May 2018, *L.W. Zhou*, LWZ 20180513-6 (HMAS).—**Index Fungorum typification identifier:** IF 557015

**Description of the epitype:** Basidiocarps annual, resupinate, adnate, cracked and brittle when dry. Hymenophore grandinioid to odontioid, buff to cinnamon or ochraceous. Margin paler than or concolorous with subiculum, thinning. Hyphal system monomitic; generative hyphae with clamp connections, hyaline, thick-walled, dichotomous branching, tortuous, 2.5–4.5 μm in diam. Subulate cystidia thin-walled, with wider base, gradually thinning, penetrating approximately half of their lengths through hymenium, 30–40 × 4.5–5 μm. Basidia clavate to subclavate, 20–25 × 4–5 μm, with four or rarely two sterigmata and a clamp connection at the base, occasionally covered with crystals; basidioles similar in shape to basidia, but smaller. Basidiospores ellipsoid, with a large oily drop, hyaline, smooth, thin-walled, acyanophilous, inamyloid, indextrinoid, (5.1–)5.2–7.3(–7.5) × (3.3–)3.5–5.2(–5.3) μm, L = 6.22 μm, W = 4.23 μm, Q = 1.37–1.58 (150/5).

Other specimens (paratypes) examined: Australia, Victoria, Yarra Ranges National Park, Dom Dom, on the fallen branch of *Eucalyptus*, 9 May 2018, *L.W. Zhou*, LWZ 20180509-8 (HMAS); Victoria, Marysville, Michaeldene Trail, on the fallen trunk of *Eucalyptus*, 9 May 2018, *L.W. Zhou*, LWZ 20180509-15 (HMAS, paratype); on the fallen branch of *Eucalyptus*, 9 May 2018, *L.W. Zhou*, LWZ 20180509-20 (HMAS, paratype); Victoria, Yarra Ranges National Park, Dandenong Ranges Botanic Garden, on the fallen trunk of *Eucalyptus*, 12 May 2018, *L.W. Zhou*, LWZ 20180512-14 (HMAS, paratype).

**Notes:***Xylodon australis* was originally described in 1859 based on a specimen collected from Tasmania, Australia [83,84]. This species is distinguished from all other species belonging to *Hyphodontia* sensu lato by its unusually dark hymenophoral color. The Australian specimens examined in this study are morphologically identical to the detailed descriptions of the holotype by Hjortstam [83] and Greslebin et al. [84]. As the holotype is very old and it would be almost certainly impossible to obtain any molecular sequence, a sequenced specimen collected from Tasmania is designated as an epitype. Moreover, five additional specimens from Victoria, Australia fell together with the epitype in a strongly supported clade in the phylogenies based on ITS (Figure 1), three genes (Figure 2) and seven genes (Figure 5). Furthermore, four of these collections were confirmed to match the morphological characters of the holotype of *X. australis*.

Greslebin et al. [84] and Hjortstam & Ryvarden [27] considered *X. australis* to be distributed also in South America but did mention essential morphological differences among specimens from different geographic regions. Given that two Chinese specimens (LWZ 20180920-12a and LWZ 20180922-47) with similar hymenophoral color as well as other morphological characters to *X. australis* represent an independent species (described below as *X. yunnanensis*), the South American specimens designated as *X. australis* are quite possibly an undescribed species. Further molecular evidence is needed to clarify this issue.

***Xylodon brevisetus*** (P. Karst.) Hjortstam & Ryvarden, Syn. Fung. (Oslo) 26: 35. 2009.

**Basionym:***Kneiffia breviseta* P. Karst., Hedwigia 25: 232. 1886.

**Notes:** Three collections previously treated as *Xylodon brevisetus* were separated into two quite separated lineages in the current ITS-based phylogeny. Firstly, two collections formed a strongly supported clade sister to *X. crystalliger*. Secondly, a single collection KHL 12386 was sister to *X. victoriensis*. In addition, two collections annotated as *X*. cf. *brevisetus* formed a strongly supported clade sister to the undescribed collection LWZ 20180922-26 from Yunnan, China (Figure 1). We apply the name *X. brevisetus* to the collection KHL 12386 from Sweden, which was also utilized in the phylogeny based on the combined dataset of ITS, nLSU and mt-SSU regions (Figure 2) where it was also placed sister to *X. victoriensis*. The status of the other ‘*X. brevisetus*’ and *X*. cf. *brevisetus* lineages identified in the ITS tree awaits clarification.

**Figure 24:**
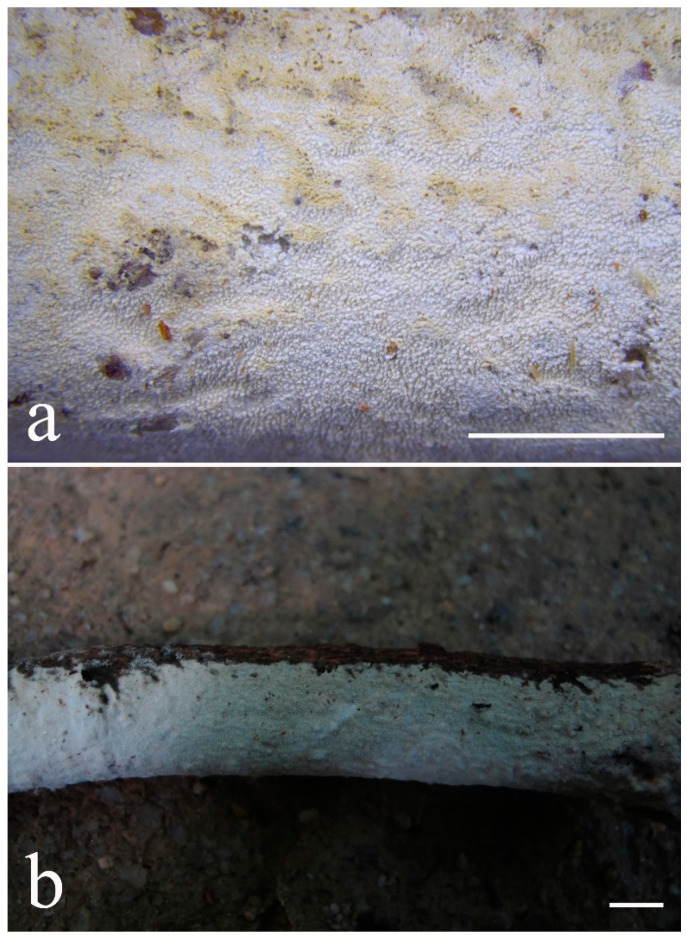
A basidiocarp of *Xylodon damansaraensis* (LWZ 20180417-20, holotype).—Scale bars: **a** = 5 mm; **b** = 1 cm.

**Figure 25:**
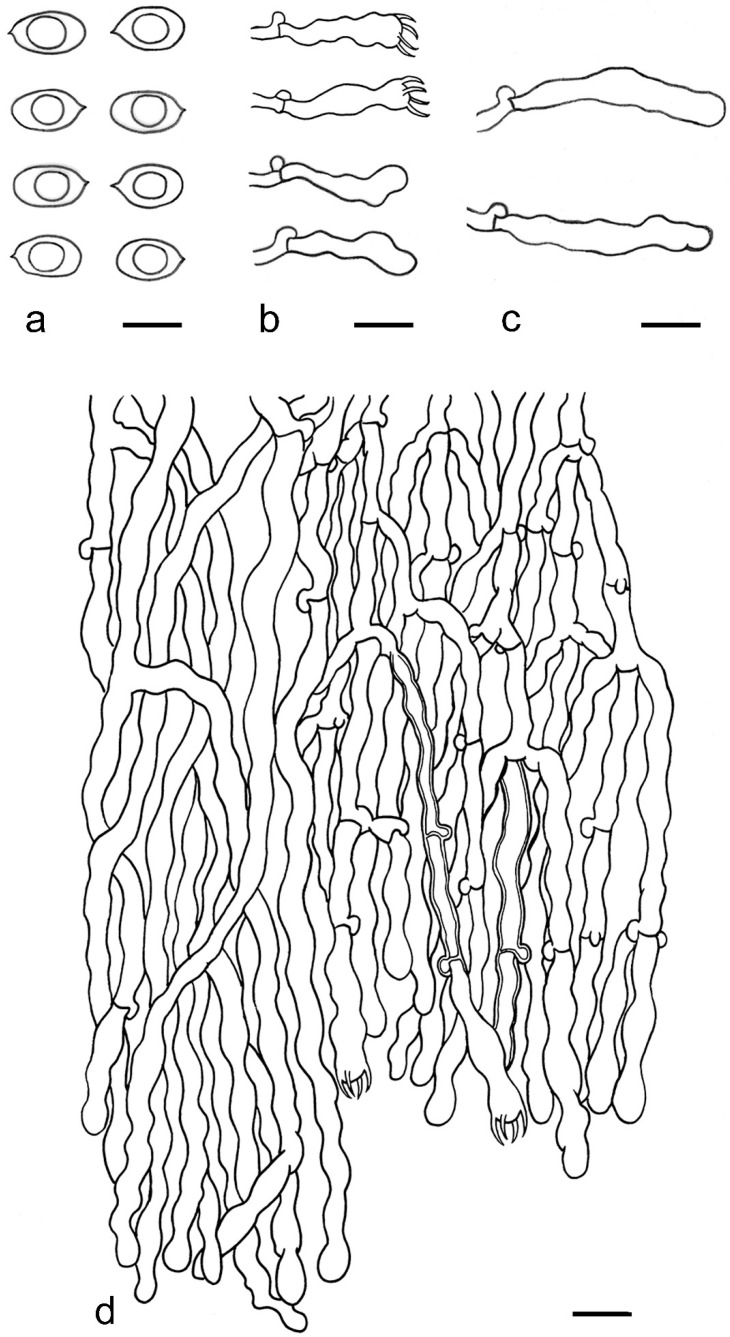
Microscopic structures of *Xylodon damansaraensis* (drawn from the holotype). (**a**) Basidiospores; (**b**) basidioles; (**c**) clavate-sinuous to submoniliform cystidia; (**d**) a section of the basidiocarp.—Scale bars: **a** = 5 µm; **b**–**d** = 10 µm.

***Xylodon damansaraensis*** Xue W. Wang & L.W. Zhou, **sp. nov.** (Figure 24 and Figure 25)

**Index Fungorum identifier:** IF 557816

**Etymology:***Damansaraensis* (Latin), refers to Kota Damansara Community Forest Reserve.

**Type:** Malaysia, Selangor, Kota Damansara Community Forest Reserve, on fallen angiosperm twig, 17 April 2018, *L.W. Zhou*, LWZ 20180417-20 (holotype HMAS).

**Description:** Basidiocarps annual, resupinate, adnate, cracked and brittle when dry. Hymenophore grandinioid to slightly odontioid, white to cream. Margin paler than or concolorous with subiculum, thinning. Hyphal system monomitic; generative hyphae with clamp connections, hyaline, thin-walled to slightly thick-walled, dichotomous branching, tortuous, 2.5–5.5 μm in diam. Clavate-sinuous to submoniliform cystidia, 35–40 × 6–7 μm. Basidia subclavate or subcapitate, 30–35 × 5.5–6.5 μm, with four sterigmata and a clamp connection at the base; basidioles similar in shape to basidia, but smaller. Basidiospores narrowly ellipsoid, with a large oily drop, hyaline, smooth, thin-walled, acyanophilous, inamyloid, indextrinoid, (5.1–)5.2–5.7(–5.8) × (2.2–)2.3–3.1(–3.2) μm, L = 5.39 μm, W = 2.57 μm, Q = 1.99–2.18 (90/3).

Other specimens (paratypes) examined: Malaysia, Selangor, Kota Damansara Community Forest Reserve, on a dead twig of living angiosperm, 17 Apr. 2018, *L.W. Zhou*, LWZ 20180417-22 (HMAS, paratype); on dead standing small angiosperm, 17 Apr. 2018, *L.W. Zhou*, LWZ 20180417-23 (HMAS, paratype).

**Notes:** *Xylodon damansaraensis* is characterized by the clavate-sinuous to submoniliform cystidia, which make it similar to *X. brevisetus*, *X. crassisporus*, *X. spathulatus* and *X. subclavatus*. *Xylodon brevisetus* and *X. spathulatus* differ from *X. damansaraensis* in the presence of capitate cystidia and slightly wider basidiospores (3–4 µm in width for both species) [19]. *Xylodon crassisporus* differs in the presence of capitate cystidia, thick-walled hyphae and thick-walled, wider basidiospores (4–4.5 µm in width) [25]. *Xylodon subclavatus* differs in the presence of hyphoid and capitate to subcapitate cystidia, and wider basidiospores (3.5–4 µm in width) [85].

***Xylodon kunmingensis*** L.W. Zhou & C.L. Zhao, in Shi, Wang, Zhou & Zhao, Mycoscience 60: 186. 2019. published 6 Feb.

= *Xylodon exilis* Yurchenko, Riebesehl & E. Langer, in Riebesehl, Yurchenko, Nakasone & Langer, MycoKeys 47: 107. 2019. published 28 Feb.

**Notes:***Xylodon kunmingensis* was described from Yunnan, China [42], while *X. exilis* was described based on specimens from Island of Taiwan [18]. Both species share a cream, odontioid hymenophore, thick-walled subicular hyphae, capitate cystidia, encrusted hyphal endings, and narrowly ellipsoid basidiospores similar in size [18,42]. Phylogenetically, the ITS-based tree grouped all eight type specimens of these two species and two newly sequenced specimens from Hubei and Liaoning Provinces, China, into a strongly supported clade (Figure 1). Similarly, in the tree inferred from the combined dataset of ITS, nLSU and mt-SSU regions all three samples (the holotype of *X. exilis* and the two newly sequenced specimens) formed a fully supported clade (Figure 2). So, from both morphological and phylogenetic perspectives, *X. exilis* and *X. kunmingensis* are conspecific. *Xylodon kunmingensis* has priority as the correct name of this species.

**Figure 26:**
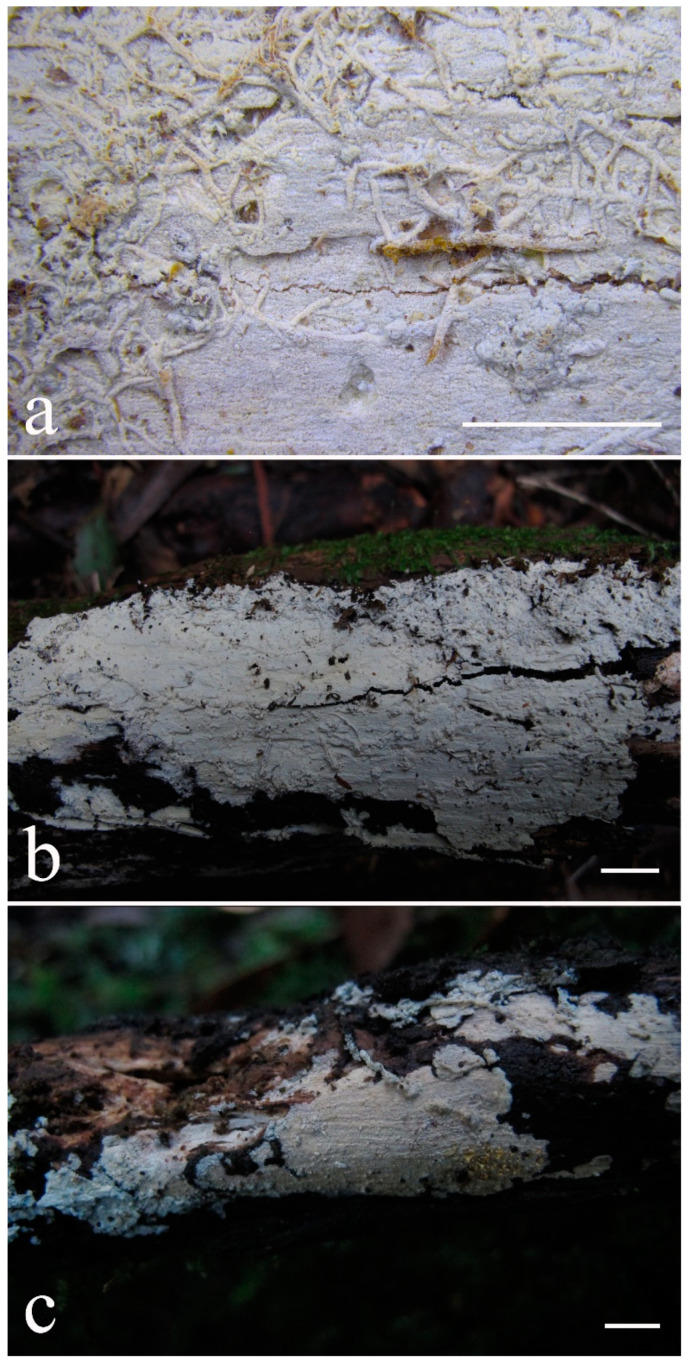
Basidiocarps of *Xylodon lagenicystidiatus*. (**a**,**b**) LWZ 20180513-16 (holotype); (**c**) LWZ 20180515-1 (paratype).—Scale bars: **a** = 5 mm; **b** = 1 cm; **c** = 2 cm.

**Figure 27:**
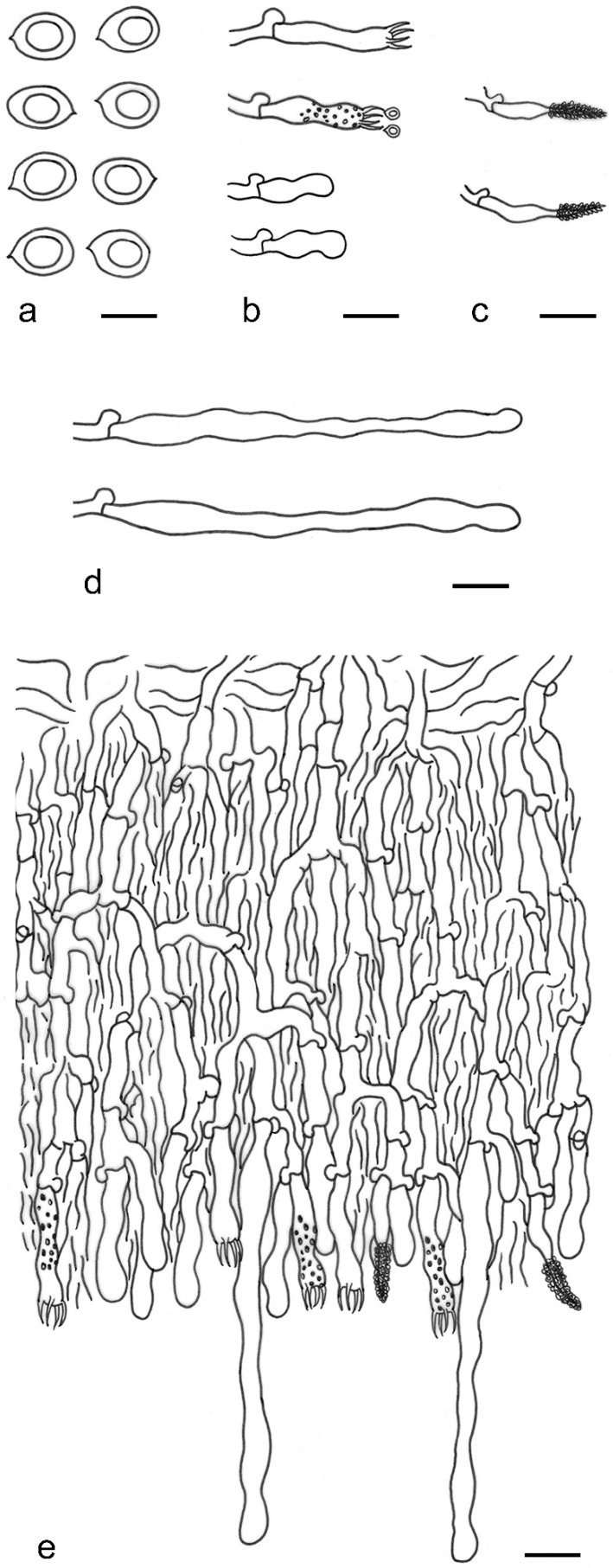
Microscopic structures of *Xylodon lagenicystidiatus* (drawn from the holotype). (**a**) Basidiospores; (**b**) basidia; (**c**) lagenocystidia; (**d**) leptocystidia; (**e**) a section of the basidiocarp.—Scale bars: **a** = 5 µm; **b**–**e** = 10 µm.

***Xylodon lagenicystidiatus*** Xue W. Wang & L.W. Zhou, **sp. nov.** (Figure 26 and Figure 27)

**Index Fungorum identifier:** IF 557855

**Etymology:***Lagenicystidiatus* (Latin), refers to lagenocystidia.

**Type:** Australia, Tasmania, Hobart, Mount Wellington, on fallen angiosperm trunk, 13 May 2018, *L.W. Zhou*, LWZ 20180513-16 (holotype MEL, isotype HMAS).

**Description:** Basidiocarps annual, resupinate, adnate, cracked and brittle when dry. Hymenophore smooth to grandinioid, white to cream. Margin paler than or concolorous with subiculum, abrupt. Hyphal system monomitic; generative hyphae with clamp connections, hyaline, thin-walled, dichotomous branching, tortuous, 2.5–4.5 μm in diam. Cystidia of two types: (a) leptocystidia thin-walled, with a wider base, gradually thinning, penetrating approximately half of their lengths through hymenium, 80–85 × 4–5 μm; (b) lagenocystidia thin-walled, with broad bases tapering abruptly towards the apices, apically encrusted, 20–25 × 3.5–4.5 μm. Basidia utriform or subclavate, 20–25 × 3.5–4.5 μm, with four sterigmata and a clamp connection at the base, encrusted with granular crystals; basidioles similar in shape to basidia, but smaller. Basidiospores ellipsoid, with a large oily drop, hyaline, smooth, thin-walled, acyanophilous, inamyloid, indextrinoid, (4.5–)4.6–5.3(–5.4) × (2.7–)2.8–3.3(–3.4) μm, L = 5.01 μm, W = 3.07 μm, Q = 1.62–1.66 (90/3).

Other specimens (paratypes) examined: Australia, Tasmania, Tahune Adventures, The Look-in Look-out, on fallen angiosperm twig, 15 May 2018, *L.W. Zhou*, LWZ 20180515-1 (HMAS, paratype); on fallen angiosperm branch, 15 May 2018, *L.W. Zhou*, LWZ 20180515-14 (HMAS, paratype).

**Notes:***Xylodon lagenicystidiatus* is distinct in this genus by the presence of lagenocystidia, which is the typical type of cystidia in *Hyphodontia*. Meanwhile, the presence of leptocystidia distinguishes *X. lagenicystidiatus* from species of *Hyphodontia*.

***Xylodon mussooriensis*** Samita, Sanyal & Dhingra ex L.W. Zhou & T.W. May, **sp. nov.**

**Index Fungorum identifier:** IF 557856

Based on *Xylodon mussooriensis* Samita, Sanyal & Dhingra (as ‘*mussoriensis*’), in Dhingra, Proceedings of the 8th International Conference on Mushroom Biology and Mushroom Products (ICMBMP8) 2014: 31. 2014, invalid name, not effectively published.

**Holotype:** India, Uttarakhand, Dehradun, Mussoorie, Mall Road, on angiosperm log, 20 Aug. 2010, Samita 6207 (PUN).

**Description:** Basidiocarp resupinate, effused, adnate, up to 320 μm thick in section; hymenial surface odontioid, aculei dense, conical, pale orange to grayish orange when fresh, not changing much on drying; margins thinning; paler concolorous, to indeterminate. Hyphal system monomitic. Generative hyphae, branched, septate, clamped; basal hyphae up to 4.5 μm wide, parallel to the substrate, encrusted, thick–walled, loosely arranged; subhymenial hyphae up to 3.5 μm wide, vertical, thin-walled, compactly arranged. Cystidia like hyphal ends none. Basidia 21.0–24.0 × 4.1–5.2 μm, narrowly clavate, somewhat sinuous, 4–sterigmate, with basal clamp; sterigmata up to 4.0 μm long. Basidiospores 5.2–5.8 × 3.1–3.5 μm, ellipsoid to broadly ellipsoid, smooth, thin-walled, with oily contents, inamyloid, acyanophilous.

**Notes:** This species was published with a detailed description in a conference paper that is available online [82], but not in a form that is effectively published, as individual papers are available separately and there does not appear to be a title page with an ISSN or ISBN. Index Fungorum has considered the name to be invalid on the basis of the lack of effective publication, and therefore we validate it here by reproducing the type citation and original description.

***Xylodon nesporii*** (Bres.) Hjortstam & Ryvarden [as ‘*nespori*’], Syn. Fung. (Oslo) 26: 38. 2009.

**Basionym:***Odontia nesporii* Bres. [as ‘*nespori*’], Annls mycol. 18(1/3): 43. 1920.

**Notes:***Xylodon nesporii* is a well-known cosmopolitan species. In the ITS-based phylogeny (Figure 1), the strongly supported clade of *X. nesporii* was composed of 26 collections including 13 newly studied specimens. Within this clade, two specimens from Hubei Province, China, viz. LWZ 20170815-13 and LWZ 20170815-13a, five Australian specimens, viz. LWZ 20180509-4, LWZ 20180509-14, LWZ 20180514-1, LWZ 20180514-12 and LWZ 20180514-13, and two specimens from Yunnan Province, China (LWZ 20180921-35) and Vietnam (LWZ 20171016-12), respectively, formed three strongly supported subclades (Figure 1). Moreover, in the phylogenies based on three genes (Figure 2) and seven genes (Figure 5), these three subclades were all strongly supported. However, other collections failed to accordingly form their own subclades (Figure 1, Figure 2 and Figure 5), which makes the subclades non-reciprocal monophyletic. Generally, this cosmopolitan species could not be further segregated according to geographic origins on the basis of the current molecular evidence. More comprehensive sampling worldwide with the help of multilocus phylogenetic analyses is needed to further clarify if there is taxonomically significant phylogenetic structure within *X. nesporii*.

***Xylodon niemelaei*** (Sheng H. Wu) Hjortstam & Ryvarden, Syn. Fung. (Oslo) 26: 38. 2009.

**Basionym:***Hyphodontia niemelaei* Sheng H. Wu, Acta bot. fenn. 142: 98. 1990.

= *Hyphodontia rhizomorpha* C.L. Zhao, B.K. Cui & Y.C. Dai, Cryptog. Mycol. 35(1): 92. 2014.

≡ *Xylodon rhizomorphus* (C.L. Zhao, B.K. Cui & Y.C. Dai) Riebesehl, Yurchenko & Langer, in Riebesehl & Langer, Mycol. Progr. 16(6): 649. 2017.

= *Hyphodontia reticulata* C.C. Chen & Sheng H. Wu, in Chen, Wu & Chen, Mycol. Progr. 16(5): 558. 2017.

≡ *Xylodon reticulatus* (C.C. Chen & Sheng H. Wu) C.C. Chen & Sheng H. Wu, in Chen, Wu & Chen, Mycoscience 59: 349. 2018.

= *Xylodon jacobaeus* J. Fernández-López, M. Dueñas, M.P. Martín & Telleria, in Crous et al., Persoonia 41: 413. 2018.

**Notes:** *Xylodon niemelaei* was first described as a poroid species of *Hyphodontia* [30], and later combined in *Xylodon* [10]. *Xylodon rhizomorphus* and *X. reticulatus* were both recently segregated from *X. niemelaei* as new species of *Hyphodontia* [15,86] and then combined in *Xylodon* [16,35]. Soon after that, *Xylodon jacobaeus* closely related to the above-mentioned three species was newly described [39]. However, morphological differences are not distinct and stable among these four species [15,30,39,86]. Moreover, the original ITS-based phylogenies did not clearly separate these four species. For example, in Zhao et al. [86], only a single collection of *X. niemelaei* was included to demonstrate *X. rhizomorphus* as an independent lineage from *X. niemelaei*; in Chen et al. [15], two or three collections for each species of *X. niemelaei* (including a collection misapplied as *X. apacheriensis*), *X. reticulatus* and *X. rhizomorphus* formed three subclades in the clade being composed of these three species, but the branch lengths were not long enough to further discriminate internal subclades as independent species; in Crous et al. [39] the branch lengths were also quite short.

In the current ITS-based phylogeny, a much greater sampling of 25 collections has been included. Not all collections were clustered in strongly supported subclades in the clade being composed of *X. jacobaeus*, *X. niemelaei*, *X. reticulatus* and *X. rhizomorphus* (Figure 1). Although three and four subclades were, respectively, revealed in the phylogenies based on three genes (Figure 2) and seven genes (Figure 5), the morphological characters for each subclade are not corresponding to the original concepts of these four species. Given the above, we consider the minor morphological differences among these collections to be variations of a single species *X. niemelaei*, and treat *X. jacobaeus*, *X. reticulatus* and *X. rhizomorphus* as later synonyms.

**Figure 28:**
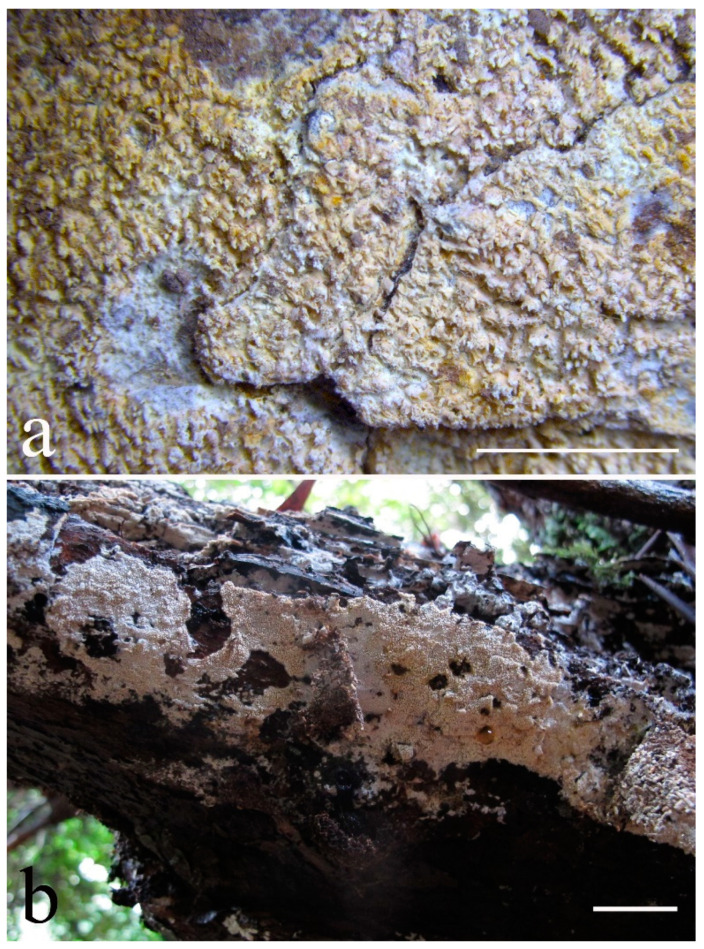
Basidiocarps of *Xylodon rhododendricola* (LWZ 20180512-4, holotype).—Scale bars: **a** = 5 mm; **b** = 2 cm.

**Figure 29:**
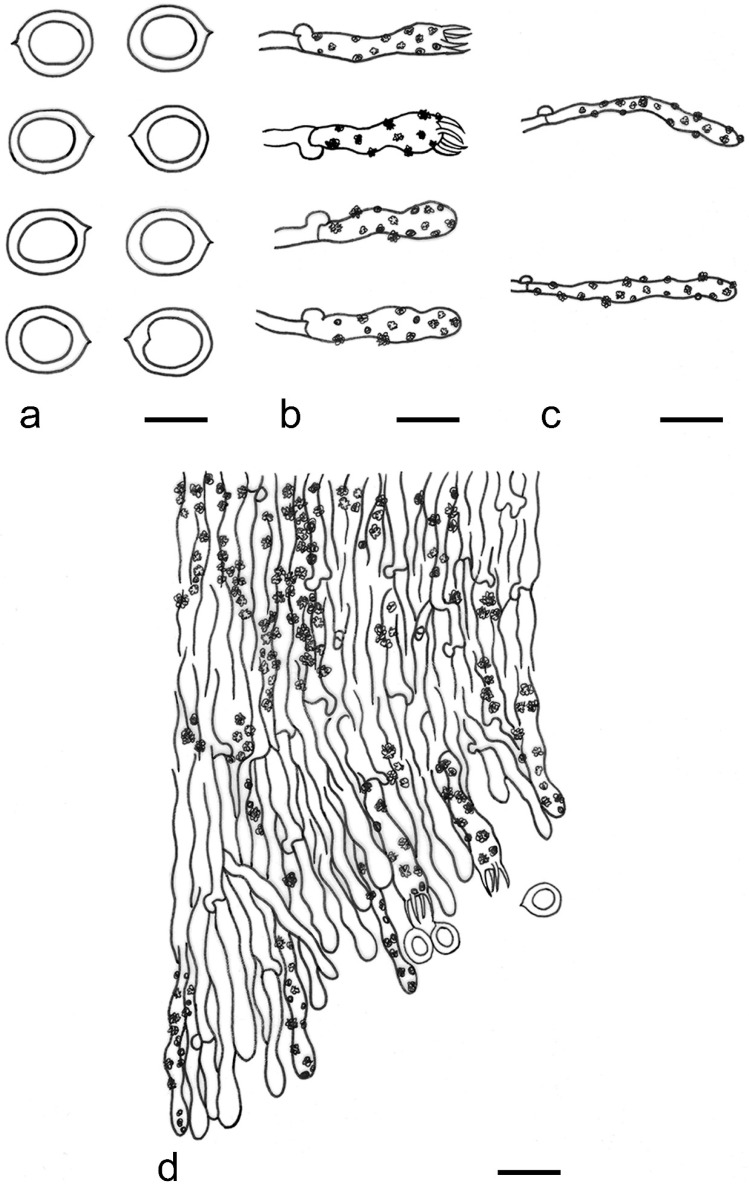
Microscopic structures of *Xylodon rhododendricola* (drawn from the holotype). (**a**) Basidiospores; (**b**) basidia and basidioles; (**c**) leptocystidia; (**d**) a section of the basidiocarp.—Scale bars: **a** = 5 µm; **b**–**d** = 10 µm.

***Xylodon rhododendricola*** Xue W. Wang & L.W. Zhou, **sp. nov.** (Figure 28 and Figure 29)

**Index Fungorum identifier:** IF 558122

**Etymology:***Rhododendricola* (Latin), refers to the host tree *Rhododendron*.

**Type:** Australia, Victoria, Yarra Ranges National Park, Dandenong Ranges Botanic Garden, on the dead base of living *Rhododendron*, 12 May 2018, *L.W. Zhou*, LWZ 20180512-4 (holotype MEL, isotype HMAS).

**Description:** Basidiocarps annual, resupinate, adnate, cracked and brittle when dry. Hymenophore odontioid, cream or light buff in young parts and buff-yellow in old parts. Margin white to cream, abrupt. Hyphal system monomitic; generative hyphae with clamp connections, hyaline, thin-walled, dichotomous branching, tortuous, usually encrusted with crystals, 2.5–4.5 μm in diam. Leptocystidia thin-walled, usually encrusted with crystals, 30–35 × 3–3.5 μm. Basidia subclavate, 25 × 3.5–5 μm, with four sterigmata and a clamp connection at the base, usually encrusted with crystals; basidioles similar in shape to basidia, but smaller. Basidiospores broadly ellipsoid, with a large oily drop, hyaline, smooth, thin-walled, cyanophilous, inamyloid, indextrinoid, (4.7–)4.8–6.5(–6.7) × (3.7–)3.8–5.1(–5.3) μm, L = 5.51 μm, W = 4.45 μm, Q = 1.18–1.27 (90/3).

Other specimens (paratypes) examined: Australia, Tasmania, Hobart, Mount Wellington, on fallen angiosperm trunk, 13 May 2018, *L.W. Zhou*, LWZ 20180513-3 (HMAS, paratype); on fallen angiosperm branch, 13 May 2018, *L.W. Zhou*, LWZ 20180513-9 (HMAS, paratype).

**Notes:** *Xylodon rhododendricola* is distinct from other species of *Xylodon* by the combination of odontioid hymenophore, encrusted leptocystidia and lack of other types of cystidia.

***Xylodon subflaviporus*** C.C. Chen & Sheng H. Wu, in Chen, Wu & Chen, Mycoscience 59: 344. 2018.

**Notes:***Xylodon subflaviporus* was named after its morphological similarity to *X. flaviporus*, and in an ITS-based phylogeny had a close relationship with *X. flaviporus* in a clade also including *X. ovisporus* [35]. However, *X. subflaviporus* was phylogenetically closer to *X. ovisporus* than to *X. flaviporus* [18]. In addition, the monophyly of *X. subflaviporus* was never well supported by previous phylogenetic analyses [18,35]. Similarly, the current ITS-based phylogeny (Figure 1) also supported that *X. subflaviporus* was closer to *X. ovisporus*, and failed to recover *X. subflaviporus* in a lineage with strong support. However, the phylogenies based on three genes (Figure 2) and seven genes (Figure 5) did strongly support *X. subflaviporus* as an independent lineage for the first time besides a closer relationship with *X. ovisporus*. Moreover, a lineage represented by a single Australian specimen LWZ 20180517-34 also appeared in the strongly supported clade with the two East Asian species *X. subflaviporus* and *X. ovisporus* (Figure 2 and Figure 5). More collections of this Australian lineage are needed before describing the lineage as new.

***Xylodon subglobosus*** Samita, Sanyal & Dhingra ex L.W. Zhou & T.W. May, **sp. nov.**

**Index Fungorum identifier:** IF 558464

Based on *Xylodon subglobosus* Samita, Sanyal & Dhingra, in Dhingra, Proceedings of the 8th International Conference on Mushroom Biology and Mushroom Products (ICMBMP8) 2014: 32. 2014, invalid name, not effectively published.

**Holotype:** India, Uttarakhand, Tehri Garhwal, Dhanaulti, on angiospermous stump, 21 Aug. 2010, Samita 6208 (PUN).

**Description:** Basidiocarp resupinate, effused, adnate, up to 300 μm thick in section; hymenial surface odontioid, aculei dense, conical, pale orange when fresh, orange gray to grayish orange on drying; margins thinning, fibrillose, paler concolorous, to indeterminate. Hyphal system monomitic. Generative hyphae, branched, septate, clamped; basal hyphae up to 4.7 μm wide, parallel to the substrate, thick-walled, loosely arranged, encrusted; subhymenial hyphae up to 3.5 μm wide, vertical, thin-walled, compactly arranged. Prominent patches of encrustation in the aculei. Cystidia like hyphal ends none. Basidia 20.0–26.0 × 4.7–5.3 μm, clavate, somewhat sinuous, 4-sterigmate, with basal clamp; sterigmata up to 4.0 μm. Basidiospores 4.2–5.2 × 3.0–5.0 μm, subglobose, smooth, thin-walled, inamyloid, acyanophilous.

**Notes:** Although the name was originally published by Dhingra [82] with a detailed description, the publication was not effectively published (see comments above under *Xylodon mussooriensis*). Here, we validate the name by reproducing the type citation and original description.

**Figure 30:**
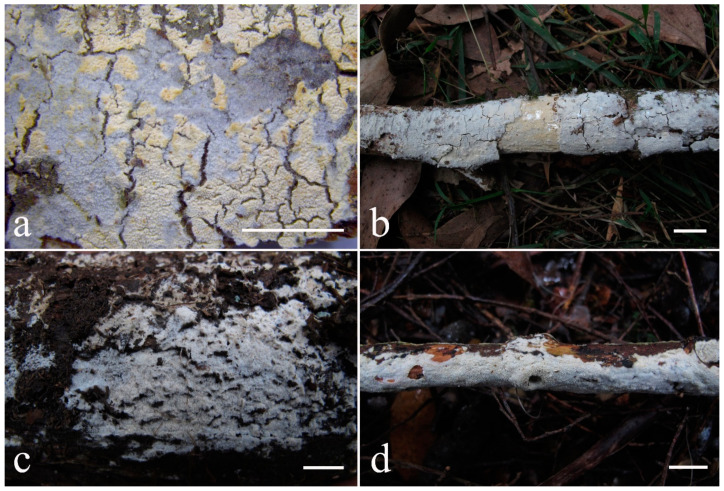
Basidiocarps of *Xylodon subserpentiformis*. (**a**) LWZ 20180513-28 (holotype); (**b**) LWZ 20180509-2 (paratype); (**c**) LWZ 20180512-16 (paratype); (**d**) LWZ 20180515-19 (paratype).—Scale bars: **a** = 5 mm; **b**,**d** = 2 cm; **c** = 1 cm.

**Figure 31:**
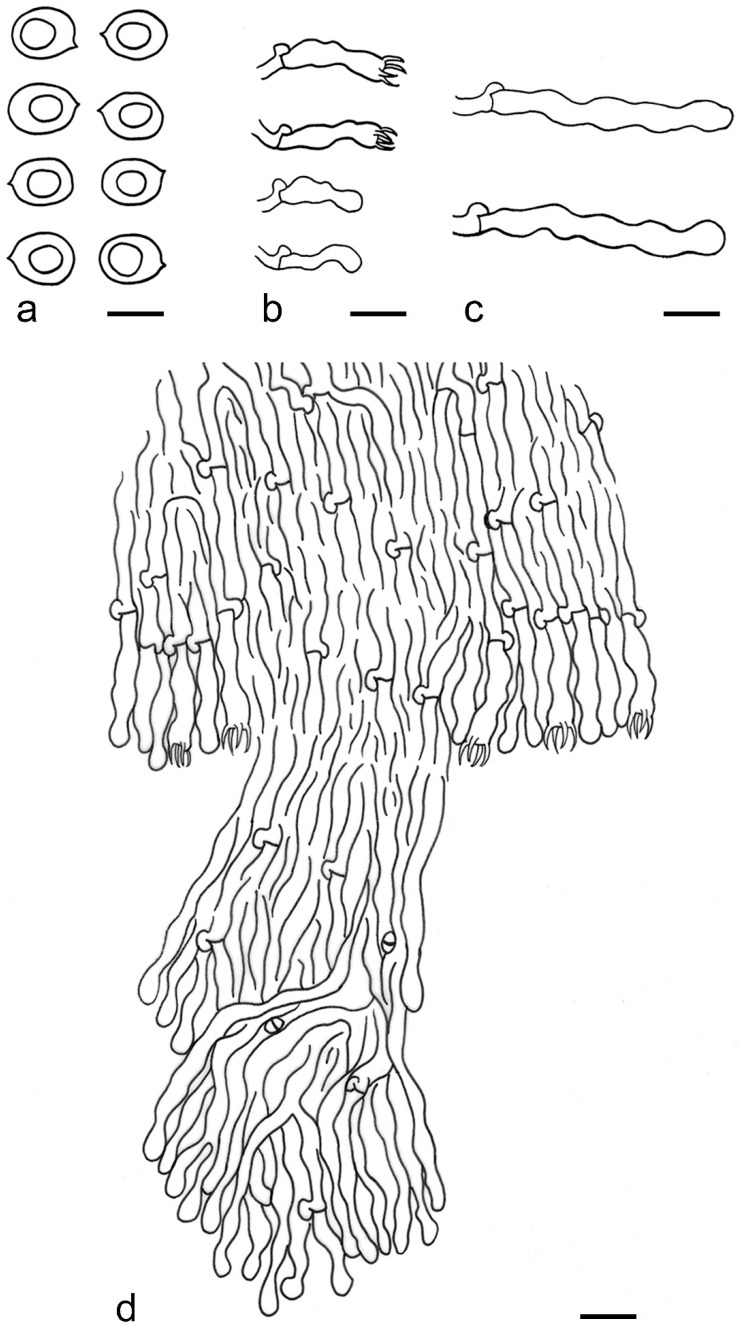
Microscopic structures of *Xylodon subserpentiformis* (drawn from the holotype). (**a**) Basidiospores; (**b**) basidia and basidioles; (**c**) tramacystidia; (**d**) a section of the basidiocarp.—Scale bars: **a** = 5 µm; **b**–**d** = 10 µm.

***Xylodon subserpentiformis*** Xue W. Wang & L.W. Zhou, **sp. nov.** (Figure 30 and Figure 31)

**Index Fungorum identifier:** IF 558465

**Etymology:***Subserpentiformis* (Latin), refers to similarity to *Xylodon serpentiformis*.

**Type:** Australia, Tasmania, Hobart, Mount Wellington, on dead standing angiosperm, 13 May 2018, *L.W. Zhou*, LWZ 20180513-28 (holotype MEL, isotype HMAS).

**Description:** Basidiocarps annual, resupinate, adnate, cracked and brittle when dry. Hymenophore grandinioid to odontioid, white to light-cream in young parts and buff to buff-yellow in old parts. Margin paler than or concolorous with subiculum, abrupt. Hyphal system monomitic; generative hyphae with clamp connections, hyaline, thin-walled, dichotomous branching, tortuous, 2.5–4.5 μm in diam. Tramacystidia in aculei, thin-walled, snake-like sinuous, 45–50 × 4.5–5.5 μm. Basidia utriform or subclavate, 20–25 × 4.5–5.5 μm, with four sterigmata about 3–4 μm long and a clamp connection at the base; basidioles similar in shape to basidia, but smaller. Basidiospores ellipsoid, with a large oily drop, hyaline, smooth, thin-walled, acyanophilous, inamyloid, indextrinoid, (4.6–)4.7–5.4(–5.5) × (2.9–)3.1–3.9(–4.1) μm, L = 5.05 μm, W = 3.31 μm, Q = 1.50–1.54 (120/4).

Other specimens (paratypes) examined: Australia, Victoria, Yarra Ranges National Park, Dom Dom, on the branch of dead standing angiosperm, 9 May 2018, *L.W. Zhou*, LWZ 20180509-2 (HMAS, paratype); Victoria, Yarra Ranges National Park, Dandenong Ranges Botanic Garden, on the fallen branch of *Eucalyptus*, 12 May 2018, *L.W. Zhou*, LWZ 20180512-16 (HMAS, paratype); Tasmania, Tahune Adventures, The Look-in Look-out, on fallen angiosperm twig, 15 May 2018, *L.W. Zhou*, LWZ 20180515-16 (HMAS, paratype); ibid., LWZ 20180515-19 (HMAS, paratype); ibid., LWZ 20180515-22 (HMAS, paratype).

**Notes:** Morphologically, *Xylodon subserpentiformis* is quite similar to *X. serpentiformis*, but differs by the absence of encrusted hyphae [19]. Moreover, the current phylogeny (Figure 1, Figure 2 and Figure 5) clearly supports the independence of these two species.

***Xylodon subtropicus*** (C.C. Chen & Sheng H. Wu) C.C. Chen & Sheng H. Wu, in Chen, Wu & Chen, Mycoscience 59: 349. 2018.

**Basionym:***Hyphodontia subtropica* C.C. Chen & Sheng H. Wu, in Chen, Wu & Chen, Mycol. Progr. 16(5): 561. 2017.

= *Poria radula* Pers., Observ. mycol. (Lipsiae) 2: 14. 1800 (1799). 

≡ *Schizopora radula* (Pers.) Hallenb., Mycotaxon 18(2): 308. 1983.

≡ *Hyphodontia radula* (Pers.) Langer & Vesterh., in Knudsen & Hansen, Nordic Jl Bot.16(2): 212. 1996.

≡ *Xylodon raduloides* Riebesehl & Langer, Mycol. Progr. 16(6): 649. 2017.

= *Xylodon laurentianus* J. Fernández-López, Telleria, M. Dueñas & M.P. Martín, in Fernández-López, Telleria, Dueñas, Wilson, Padamsee, Buchanan, Mueller & Martin, IMA Fungus 10(no. 2): 11. 2019.

= *Xylodon patagonicus* J. Fernández-López, Telleria, M. Dueñas & M.P. Martín, in Fernández-López, Telleria, Dueñas, Wilson, Padamsee, Buchanan, Mueller & Martin, IMA Fungus 10(no. 2): 13. 2019.

= *Xylodon novozelandicus* J. Fernández-López, Telleria, M. Dueñas & M.P. Martín, in Fernández-López, Telleria, Dueñas, Wilson, Padamsee, Buchanan, Mueller & Martin, IMA Fungus 10(no. 2): 14. 2019.

**Notes:***Xylodon subtropicus* is a recently described poroid species based on two Asian specimens from Vietnam and China [15]. It is very similar to *Hyphodontia radula*, and the so-called morphological distinctions between these two species actually overlap [15]. Soon after the publication of *X. subtropicus*, the name *X. raduloides* was introduced as a replacement name in *Xylodon* for *H. radula*, to avoid the competing name *Xylodon radula* (Fr.) Tura, Zmitr., Wasser & Spirin, which blocked *Poria radula*, the basionym of *H. radula*, from being combined in *Xylodon* [16]. In addition, Fernández-López et al. [41] recognized three additional species, viz. *X. laurentianus*, *X. novozelandicus* and *X. patagonicus*, segregated from *X. raduloides* based on a combination of morphological, molecular and environmental evidence. However, the morphological differences are quite minor among these four species, and some species pairs lack morphological differentiation [41]. A species delimitation analysis found that the hypothesis of recognizing the independence of each of the four species was more probable than that of accepting them as a single species; however, regarding phylogenetic evidence, only these four species and an outgroup taxon *Xylodon flaviporus* were referred to and other close species, especially *X. subtropicus*, were not included [41]. Moreover, while only the ITS-based phylogeny strongly supported the independence of each of the four species, there was considerable missing data, and the lineages were not strongly supported on nLSU region alone and the combined ITS and nLSU regions [41].

The current ITS-based phylogeny covering the most comprehensive sampling of *Xylodon* till now recovered the four lineages of *X. laurentianus*, *X. novozelandicus*, *X. patagonicus* and *X. raduloides*, each not receiving strong support; the lineage of *X. subtropicus* being composed of two original Asian specimens described by Chen et al. [15] occupied a basal position of the four lineages (Figure 1). Like the phylogeny based on ITS region (Figure 1), that based on three genes also recovered the four lineages, and no one was strongly supported, neither was the clade being composed of these four lineages (Figure 2). Alternatively, these four lineages together with the basal lineage of *X. subtropicus* formed a strongly supported clade (Figure 2). In the phylogeny based on seven genes (Figure 5), the two original specimens of *X. subtropicus* with only ITS and nLSU regions available were not included; the lineage of *X. raduloides* was strongly supported, whereas those of *X. laurentianus*, *X. novozelandicus* and *X. patagonicus* were weakly to moderately supported; the clade consisting of these four lineages was strongly supported. In these three phylogenies (Figure 2 and Figure 5), the newly sequenced Australian specimens merged in the lineage of *X. novozelandicus*. Besides the topologies, the branch lengths among these lineages are also too short to clearly distinguish species and fall within the infraspecific distances observed in several other well accepted species in the genus (Figure 2 and Figure 5). Taking into consideration the morphological similarity, the current phylogenies and the low level of divergence, we consider *X. laurentianus*, *X. novozelandicus*, *X. patagonicus*, *X. raduloides* and *X. subtropicus* to be conspecific with the last one as the correct name.

The more or less geographically structured phylogenetic signal recovered by Fernández-López et al. [41] indicates a species perhaps in the process of incipient speciation. It is also of interest that Fernández-López et al. [41] found some niche differentiation among the four lineages they recognized as species, and this differentiation merits further exploration on a wider sampling. Furthermore, Paulus et al. [87] found that New Zealand samples of *X. subtropicus* (as *S. radula*) had a high mating compatibility (73.7% crosses completely positive and 1.2% lacking clamps) but there was also compatibility between these and isolates from the Northern Hemisphere, albeit at a reduced level (47.8% crosses completely positive and 12.2% lacking clamps). *Xylodon subtropicus* presents an interesting test case for applying genome-wide markers such as restriction site-associated DNA markers (RADSeq) [88] to further investigate the boundary between population genetic and species level variation, especially if integrated with mating studies of this and closely related species.

It is noted that the Russian collection FCUG 2433 labeled as *Schizopora radula* [87] was merged into the not well supported clade of *Xylodon subtropicus* in the ITS-based phylogeny (Figure 1) but not into the strongly supported clade of *X. subtropicus* in the phylogeny based on three genes (Figure 2). Therefore, we treat it as an unnamed collection.

**Figure 32:**
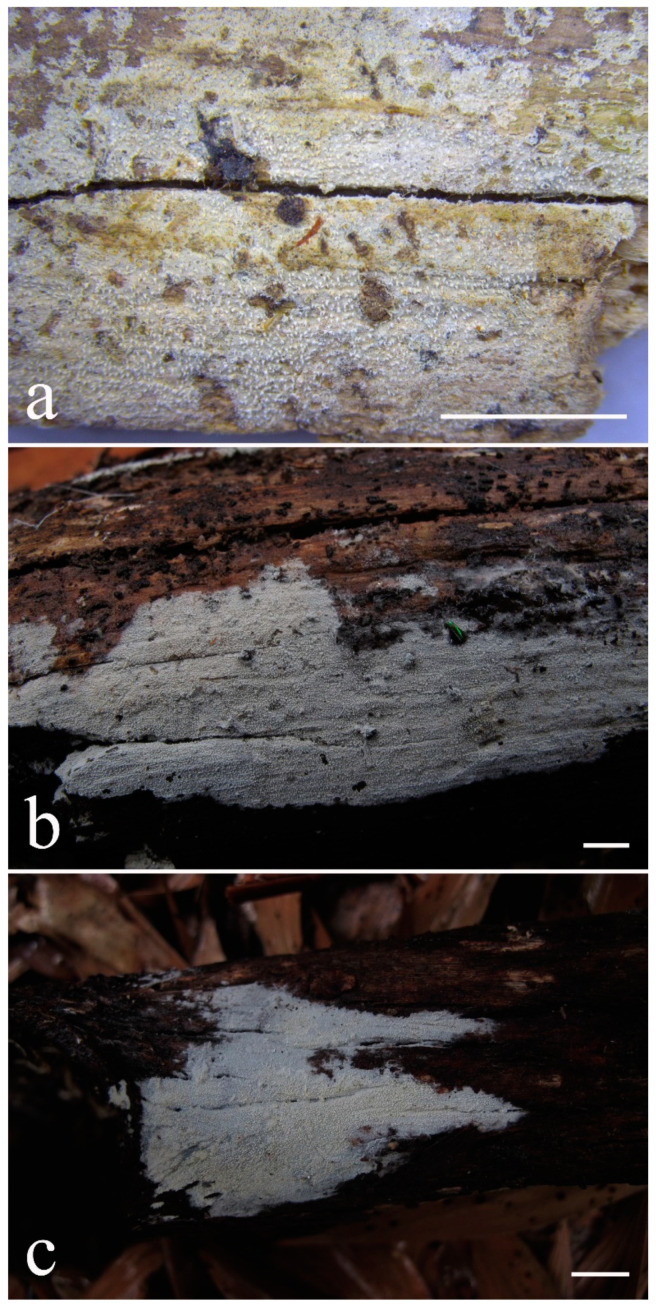
(**a**) Basidiocarps of *Xylodon victoriensis*; (**b**) LWZ 20180512-11 (holotype); (**c**) LWZ 20180510-29 (paratype).—Scale bars: **a** = 5 mm; **b**,**c** = 1 cm.

**Figure 33:**
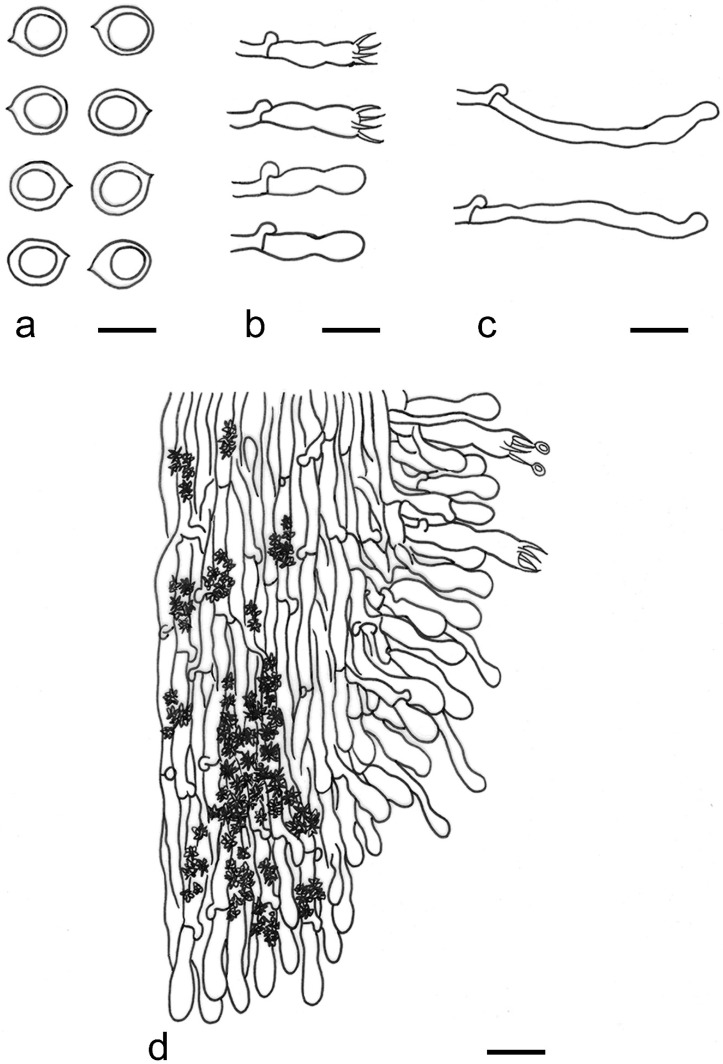
Microscopic structures of *Xylodon victoriensis* (drawn from the holotype). (**a**) Basidiospores; (**b**) basidia and basidioles; (**c**) leptocystidia; (**d**) a section of the basidiocarp.—Scale bars: **a** = 5 µm; **b**–**d** = 10 µm.

***Xylodon victoriensis*** Xue W. Wang & L.W. Zhou, **sp. nov.** (Figure 32 and Figure 33)

**Index Fungorum identifier:** IF 558466

**Etymology:***Victoriensis* (Latin), refers to Victoria, Australia.

**Type:** Australia, Victoria, Cathedral Range State Park, on the fallen angiosperm branch, 10 May 2018, *L.W. Zhou*, LWZ 20180512-11 (holotype MEL, isotype HMAS).

**Description:** Basidiocarps annual, resupinate, adnate, cracked and brittle when dry. Hymenophore tuberculate to grandinioid, white to whitish-cream in young parts and buff in old parts. Margin paler than or concolorous with subiculum, thining, pruinose. Hyphal system monomitic; generative hyphae with clamp connections, hyaline, thin-walled, dichotomous branching, tortuous, usually encrusted with crystals, 2.5–4 μm in diam. Leptocystidia thin-walled, with a wider base, gradually thinning, penetrating approximately half of their lengths through hymenium, 30–40 × 4.5–5 μm. Basidia utriform, 15 × 4–4.5 μm, with four sterigmata and a clamp connection at the base; basidioles similar in shape to basidia, but smaller. Basidiospores globose to subglobose, with a large oily drop, hyaline, smooth, thin-walled, cyanophilous, inamyloid, indextrinoid, (3.7–)3.8–4.6(–4.7) × (3.1–)3.2–3.7(–3.9) μm, L = 4.33 μm, W = 3.37 μm, Q = 1.28–1.29 (60/2).

Other specimens (paratypes) examined: Australia, Victoria, Yarra Ranges National Park, Dandenong Ranges Botanic Garden, on fallen branch of *Eucalyptus*, 12 May 2018, *L.W. Zhou*, LWZ 20180510-29 (HMAS, paratype).

**Notes:***Xylodon victoriensis* resembles *X. crustosoglobosus* by whitish to yellowish hymenophore and globose to subglobose basidiospores; however, the latter species differs in the presence of subulate cystidia or hyphal endings, the absence of leptocystidia, and larger basidiospores (5 × 3.8–4 µm) [89].

**Figure 34:**
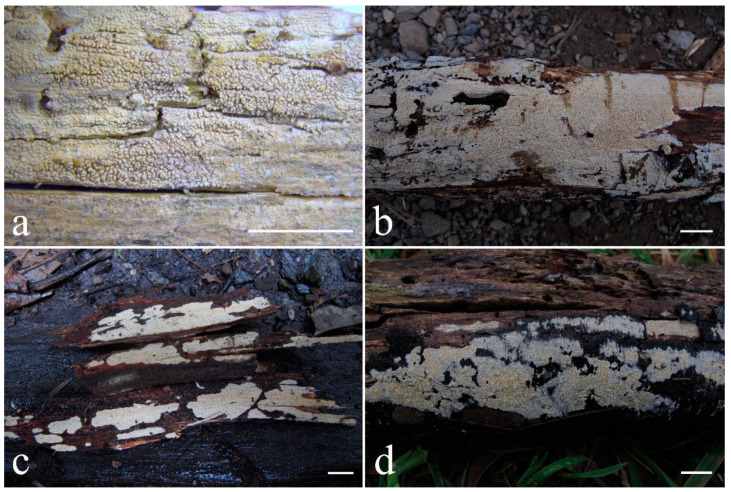
(**a**) Basidiocarps of *Xylodon yarraensis*; (**b**) LWZ 20180509-7 (holotype); (**c**) LWZ 20180510-19 (paratype); (**d**) LWZ 20180512-22 (paratype).—Scale bars: **a** = 5 mm; **b** = 1 cm; **c**,**d** = 2 cm.

**Figure 35:**
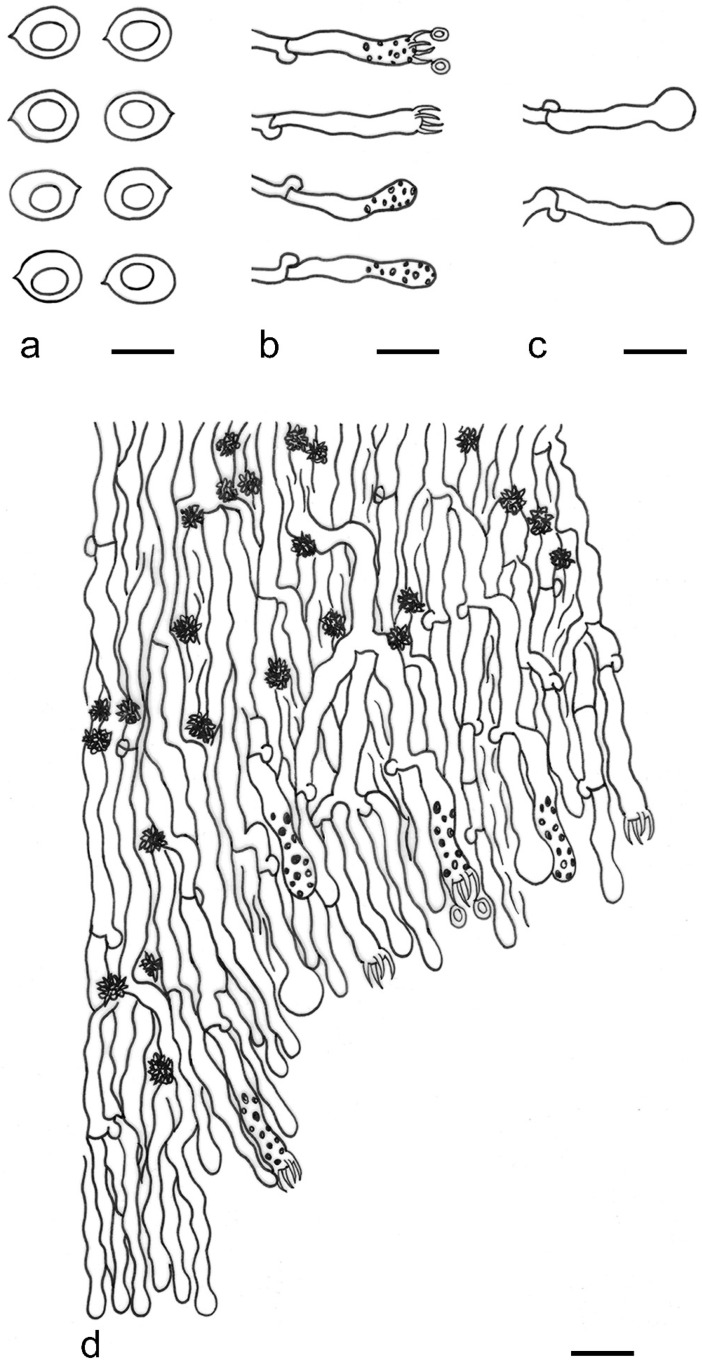
Microscopic structures of *Xylodon yarraensis* (drawn from the holotype). (**a**) Basidiospores; (**b**) basidia and basidioles; (**c**) capitate cystidia; (**d**) a section of the basidiocarp.—Scale bars: **a** = 5 µm; **b**–**d** = 10 µm.

***Xylodon yarraensis*** Xue W. Wang & L.W. Zhou, **sp. nov.** (Figure 34 and Figure 35)

**Index Fungorum identifier:** IF 558467

**Etymology:***Yarraensis* (Latin), refers to the Yarra Ranges National Park.

**Type:** Australia, Victoria, Yarra Ranges National Park, Dom Dom, on fallen angiosperm branch, 9 May 2018, *L.W. Zhou*, LWZ 20180509-7 (holotype MEL, isotype HMAS). 

**Description:** Basidiocarps annual, resupinate, adnate, cracked and brittle when dry. Hymenophore grandinioid, cream in young parts and buff to buff-yellow in old parts. Margin paler than or concolorous with subiculum, abrupt. Hyphal system monomitic; generative hyphae with clamp connections, hyaline, thin-walled, dichotomous branching, tortuous, sometimes encrusted with crystals, 2.5–4.5 μm in diam. Capitate cystidia thin-walled, 25–30 × 2.5–3.5 μm, apically about 5.5–6.5 μm in diam. Basidia subcylindrical or subclavate, 20–25 × 3.5–4.5 μm, with four sterigmata and a clamp connection at the base, usually encrusted with granular crystals. Basidioles similar in shape to basidia, but smaller. Basidiospores ellipsoid, with a large oily drop, hyaline, smooth, thin-walled, cyanophilous, inamyloid, indextrinoid, (4.4–)4.5–5.3(–5.4) × (2.9–)3.1–3.8(–3.9) μm, L = 4.81 μm, W = 3.30 μm, Q = 1.45–1.46 (120/4)

Other specimens (paratypes) examined: Australia, Victoria, Yarra Ranges National Park, Cora Lynn Falls, on fallen angiosperm trunk, 10 May 2018, *L.W. Zhou*, LWZ 20180510-19 (HMAS, paratype); Victoria, Yarra Ranges National Park, Dandenong Ranges Botanic Garden, on fallen angiosperm branch, 12 May 2018, *L.W. Zhou*, LWZ 20180512-21 (HMAS, paratype); ibid., LWZ 20180512-22 (HMAS, paratype).

**Notes:***Xylodon yarraensis* is similar to *X. rimosissimus* in the grandinioid hymenophore, presence of only capitate cystidia and thin-walled basidiospores; however, the latter species differs in its hyphae without encrustation, cystidia with exudation and slightly longer, subcylindrical to cylindrical basidiospores (5–6.5 µm in length) [19].

**Figure 36:**
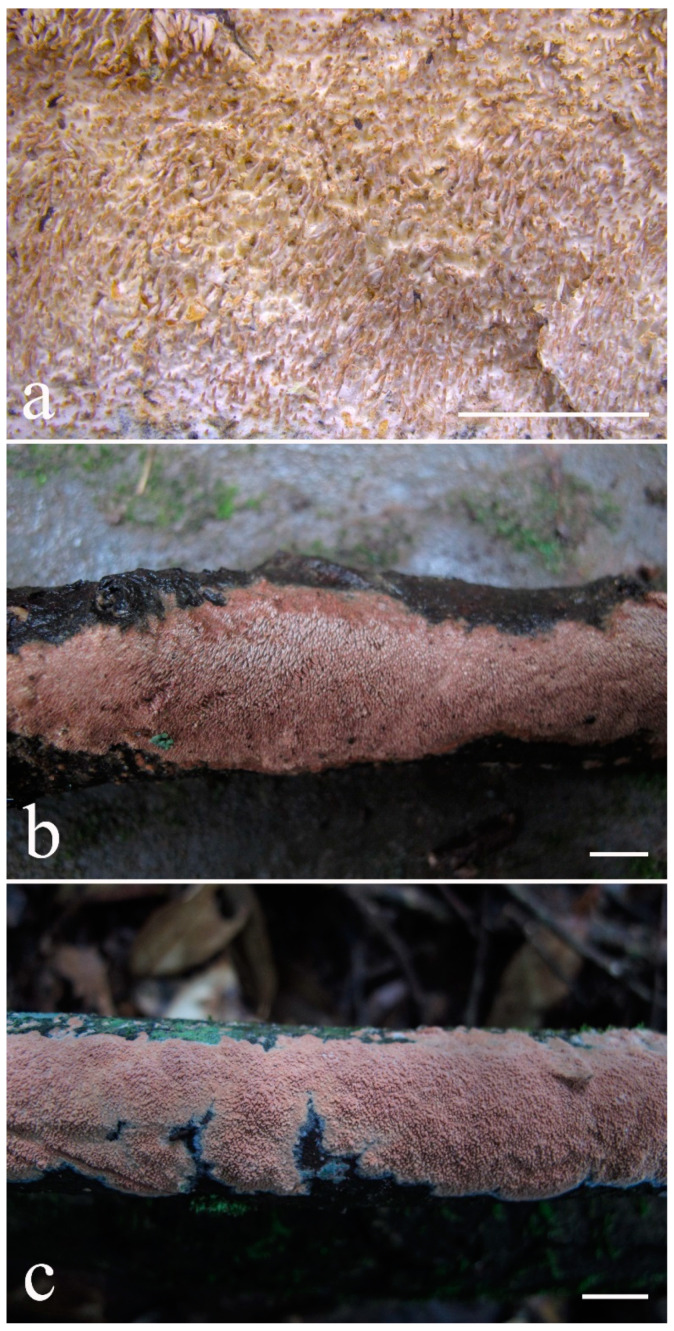
(**a**) Basidiocarps of *Xylodon yunnanensis*; (**b**) LWZ 20180920-12a (holotype); (**c**) LWZ 20180922-47 (paratype).—Scale bars: **a** = 5 mm; **b** = 2 cm; **c** = 1 cm.

**Figure 37:**
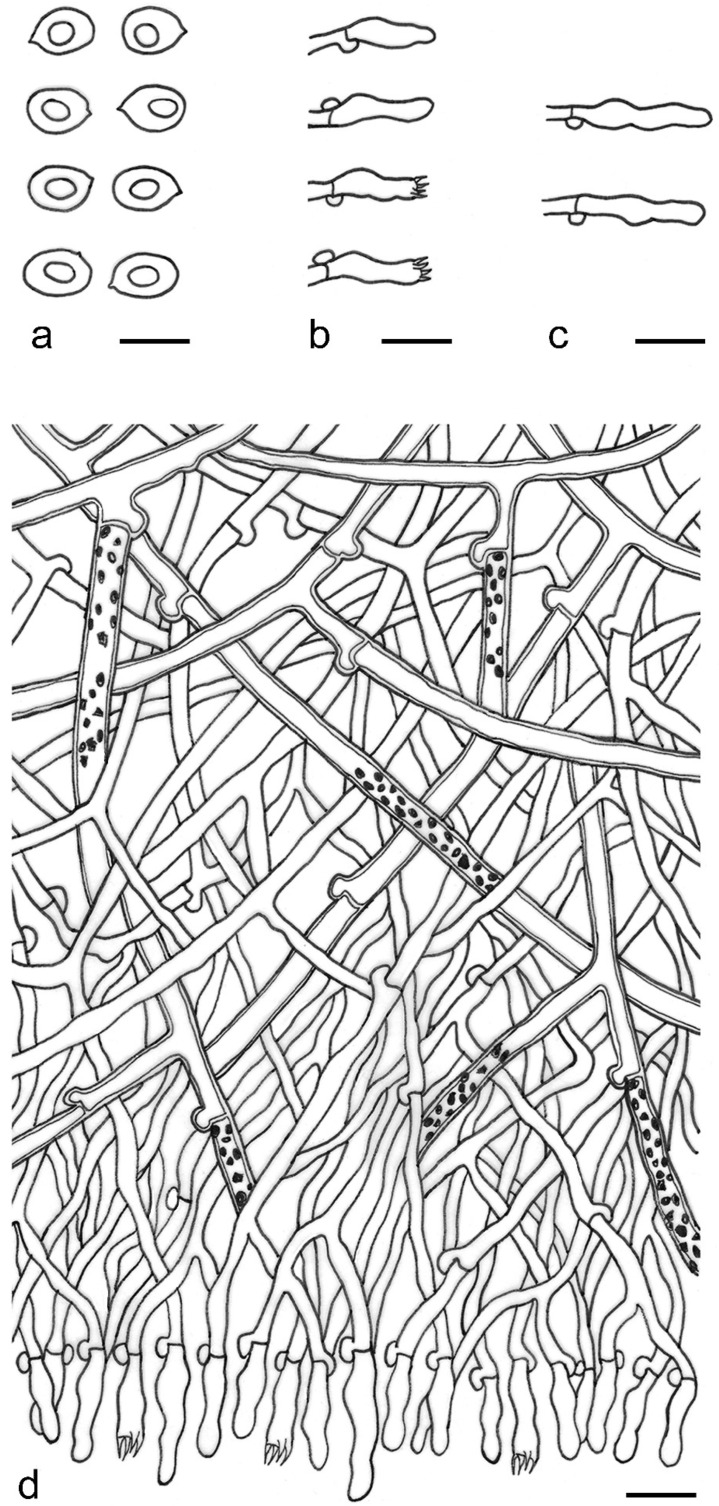
Microscopic structures of *Xylodon yunnanensis* (drawn from the holotype). (**a**) Basidiospores; (**b**) basidia and basidioles; (**c**) clavate to subclavate cystidia; (**d**) a section of the basidiocarp.—Scale bars: **a** = 5 µm; **b**–**d** = 10 µm.

***Xylodon yunnanensis*** Xue W. Wang & L.W. Zhou, **sp. nov.** (Figure 36 and Figure 37)

**Index Fungorum identifier:** IF 558469

**Etymology:***Yunnanensis* (Latin), refers to Yunnan Province, China.

**Type:** China, Yunnan, Chuxiong, Zixi Mountain Forest Park, on fallen angiosperm branch, 20 Sept. 2018, *L.W. Zhou*, LWZ 20180920-12a (holotype HMAS).

**Description:** Basidiocarps annual, resupinate, adnate, cracked and brittle when dry. Hymenophore grandinioid to odontioid, aculei up to 2 mm long, becoming gradually shorter towards margin, cinnamon to orange-brown. Margin white to buff or concolorous with subiculum, slightly fibrillose. Hyphal system monomitic to pseudodimitic; generative hyphae with clamp connections, hyaline, dichotomous branching, interwoven, occasionally encrusted with crystals, thin- to thick-walled (walls up to 0.5–1.2 μm thick), 2.5–5 μm in diam. Clavate to subclavate cystidia thin-walled, 20–24 × 3.5–5 μm. Basidia utriform or subclavate, 15–20 × 4–5 μm, with four sterigmata and a clamp connection at the base; basidioles similar in shape to basidia, but smaller. Basidiospores ellipsoid, with a large oily drop, hyaline, smooth, thin-walled, acyanophilous, inamyloid, indextrinoid, (4.1–)4.2–4.8(–4.9) × (2.8–)2.9–3.6(–3.7) μm, L = 4.54 μm, W = 3.24 μm, Q = 1.33–1.40 (60/2).

Other specimens (paratypes) examined: China, Yunnan, Baoshan, Baihua Ridge, on fallen angiosperm branch, 22 Sept. 2018, *L.W. Zhou*, LWZ 20180922-47 (HMAS, paratype).

**Notes:** The cinnamon to orange-brown color of the hymenophore makes *Xylodon yunnanensis* distinct and reminiscent of *X. australis*. However, *X. australis* has longer subulate cystidia instead of the shorter clavate to subclavate cystidia in *X. yunnanensis*, and distinctly larger basidiospores (5.2–7.3 × 3.5–5.2 µm).
**A key to 87 accepted species of *Xylodon***1a. Hymenophore odontioid or coralloid........................................................................................................................................2b. Hymenophore smooth, tuberculate, grandinioid, irpicoid or poroid....................................................................................662a. Capitate or subcapitate cystidia or cystidioles present............................................................................................................3b. Capitate or subcapitate cystidia or cystidioles absent..............................................................................................................383a. Basidiospores ellipsoid................................................................................................................................................................4b. Basidiospores globose to subglobose, ovoid, cylindrical or allantoid....................................................................................244a. Moniliform or submoniliform cystidia present........................................................................................................................5b. Moniliform or submoniliform cystidia absent..........................................................................................................................115a. Basidiospores thin-walled...........................................................................................................................................................6b. Basidiospores thick-walled...........................................................................................................................................................96a. Basidiospores up to 7.5 μm long......................................................................................................................*X. anmashanensis*b. Basidiospores up to 6 μm long......................................................................................................................................................77a. Moniliform cystidia originating only in subhymenium.....................................................................................*X. spathulatus*b. Moniliform cystidia originating only in subiculum or in subiculum and subhymenium.....................................................88a. Aculei up to 1 mm long, hymenophoral margin thinning out.............................................................................*X. brevisetus*b. Aculei up to 0.3 mm long, hymenophoral margin abrupt or byssoid.................................................................*X. subclavatus*9a. Astrocystidia present; basidiospores cyanophilous............................................................................................*X. ussuriensis*b. Astrocystidia absent; basidiospores acyanophilous................................................................................................................1010a. Hyphoid or subulate cystidia absent.................................................................................................................*X. crassisporus*b. Hyphoid or subulate cystidia present...............................................................................................................*X. anmashanensis*11a. Hymenophore coralloid...............................................................................................................................................*X. archeri*b. Hymenophore odontioid.............................................................................................................................................................1212a. Basidia cylindrical to subcylindrical.......................................................................................................................................13b. Basidia utriform............................................................................................................................................................................1513a. Pin- or sting-shaped cystidia present........................................................................................................................*X. borealis*b. Pin- or sting-shaped cystidia absent...........................................................................................................................................1414a. Aculei 0.03–0.12 mm long, 8–14 per mm; basidiospores thin- or slightly thick-walled............................*X. pseudolanatus*b. Aculei 0.13–0.35 mm long, 4 per mm; basidiospores thin-walled........................................................................*X. vesiculosus*15a. Astrocystidia present.....................................................................................................................................*X. astrocystidiatus*b. Astrocystidia absent.....................................................................................................................................................................1616a. Only capitate cystidia and leptocystidia present......................................................................................*X. heterocystidiatus*b. Different combinations of cystidia.............................................................................................................................................1717a. Besides capitate to subcapitate cystidia, tramacystidia, subulate or apically encrusted hyphoid cystidia present........................................................................................................................................................................................18b. Only capitate cystidia present.....................................................................................................................................................2218a. Tramacystidia present..............................................................................................................................................................19b. Tramacystidia absent...................................................................................................................................................................2019a. Capitate cystidia slightly thick-walled; basidiospores thick-walled, more than 4 µm wide........................*X. magnificus*b. Capitate cystidia thin-walled; basidiospores thin-walled, less than 4 µm wide......................................................*X. lanatus*20a. Subulate cystidia present.......................................................................................................................................*X. pruniaceus*b. Subulate cystidia absent...............................................................................................................................................................2121a. Hymenophore cream; hyphoid cystidia sometimes with stellate crystalline cap..........................................*X. attenuatus*b. Hymenophore white; hyphoid cystidia often heavily encrusted and rarely with stellate crystalline cap.....*X. crystalliger*22a. Basidia subclavate.....................................................................................................................................................*X. capitatus*b. Basidia sinuous or utriform.........................................................................................................................................................2323a. Basidiospores thin-walled, more than 3.5 µm wide.................................................................................................*X. asperus*b. Basidiospores slightly thick-walled, less than 3.5 µm wide.............................................................................*X. kunmingensis*24a. Hymenophore coralloid or odontioid with fimbriate aculei...............................................................................................25b. Hymenophore odontioid without fimbriate aculei..................................................................................................................2825a. Aculei up to 3 mm long............................................................................................................................................*X. quercinus*b. Aculei up to 1 mm long................................................................................................................................................................2626a. Basidiospores more than 3 μm wide...........................................................................................................................*X. archeri*b. Basidiospores less than 3 μm wide.............................................................................................................................................2727a. Aculei up to 200 μm long; basidiospores less than 6 μm long................................................................................*X. nesporii*b. Aculei up to 600 μm long; basidiospores more than 6 μm long................................................................................*X. ramicida*28a. Basidiospores more than 7 μm long........................................................................................................................................29b. Basidiospores less than 7 μm long..............................................................................................................................................3029a. Basidiospores ovoid to allantoid, less than 5 µm wide..................................................................................*X. adhaerisporus*b. Basidiospores globose to subglobose, more than 5 µm wide..........................................................................................*X. follis*30a. Basidiospores slightly thick-walled........................................................................................................................................31b. Basidiospores thin-walled...........................................................................................................................................................3231a. Pin- or sting-like cystidia abundant; basidiospores slightly cylindrical, ellipsoid or broadly ellipsoid...........*X. borealis*b. Pin- or sting-like cystidia absent; basidiospores globose or subglobose.......................................................*X. hyphodontinus*32a. Basidiospores cylindrical or subcylindrical.....................................................................................................*X. rimosissimus*b. Basidiospores differently shaped...............................................................................................................................................3333a. Moniliform cystidia scattered..............................................................................................................................*X. subclavatus*b. Moniliform cystidia absent..........................................................................................................................................................3434a. Fusiform cystidia present in aculei............................................................................................................................*X. hastifer*b. Fusiform cystidia absent..............................................................................................................................................................3535a. Basidiospores less than 5 μm long..........................................................................................................................................36b. Basidiospores more than 5 μm long...........................................................................................................................................3736a. Subcylindrical and hyphoid to narrowly ventricose cystidia absent....................................................................*X. tenellus*b. Subcylindrical and hyphoid to narrowly ventricose cystidia present......................................................................*X. filicinus*37a. Capitate cystidia in hymenium and subhymenium, capitate hyphal endings in subiculum and aculei…......*X. asperus*b. Capitate gloeocystidia or hyphal endings only in hymenium and/or aculei.........................................................*X. capitatus*38a. Subulate cystidia present.........................................................................................................................................................39b. Subulate cystidia absent...............................................................................................................................................................4839a. Basidiospores ellipsoid or narrowly ellipsoid.......................................................................................................................40b. Basidiospores cylindrical, allantoid, ovoid or subglobose......................................................................................................4540a. Leptocystidia present.................................................................................................................................................*X. australis*b. Leptocystidia absent....................................................................................................................................................................4141a. Hyphoid and moniliform to submoniliform cystidia present....................................................................*X. anmashanensis*b. Hyphoid and moniliform to submoniliform cystidia absent..................................................................................................4242a. Basidiospores often glued together in packs of two to six basidiospores...................................................*X. candidissimus*b. Basidiospores separated..............................................................................................................................................................4343a. Subulate cystidia thin- or slightly thick-walled, less than 30 μm long......................................................*X. submucronatus*b. Subulate cystidia thin-walled, more than 30 μm long..............................................................................................................4444a. Basidia utriform with one or two constrictions; subulate cystidia up to 75 μm long.....................................*X. knysnanus*b. Basidia utriform to galzinoid with several constrictions; subulate cystidia up to 50 μm long...........................*X. nudisetus*45a. Basidiospores ovoid to allantoid, more than 7 μm long................................................................................*X. adhaerisporus*b. Basidiospores cylindrical or subglobose, less than 7 μm long................................................................................................4646a. Hymenophore greyish-white to yellowish; subulate cystidia or hyphal endings................................*X. crustosoglobosus*b. Hymenophore white to cream; subulate cystidia up to 50 or 75 μm long..............................................................................4747a. Basidia utriform with one or two constrictions; subulate cystidia up to 75 μm long.....................................*X. knysnanus*b. Basidia utriform to galzinoid with several constrictions; subulate cystidia up to 50 μm long...........................*X. nudisetus*48a. Moniliform or submoniliform cystidia present....................................................................................................................49b. Moniliform or submoniliform cystidia absent..........................................................................................................................5149a. Only moniliform cystidia present...................................................................................................................................*X. lenis*b. Other types of cystidia present....................................................................................................................................................5050a. Hyphoid cystidia and rare capitate cystidioles present..............................................................................*X. anmashanensis*b. Cylindrical gloeocystidia and subclavate cystidia present.............................................................................*X. subscopinellus*51a. Basidiospores suballantoid or subglobose.............................................................................................................................52b. Basidiospores ellipsoid or cylindrical........................................................................................................................................5452a. Basidiospores suballantoid, more than 6 µm long.................................................................................................*X. syringae*b. Basidiospores subglobose, less than 6 µm long........................................................................................................................5353a. Capitate hyphal endings present in aculei....................................................................................................................*X. rudis*b. Capitate hyphal endings absent..............................................................................................................................*X. subglobosus*54a. Cystidia or cystidioles absent..................................................................................................................................................55b. Cystidia or cystidioles present....................................................................................................................................................5655a. Basidiospores narrowly ellipsoid to cylindrical, more than 6 µm long, less than 3 µm wide.........................*X. nesporina*b. Basidiospores ellipsoid, less than 6 µm long, more than 3 µm wide...............................................................*X. mussooriensis*56a. Cylindrical, clavate to subclavate, spathuliform cystidia or leptocystidia present..........................................................57b. Tubular, ventricose, pyriform, hyphoid or sinuous cystidia present.....................................................................................6057a. Clavate to spathuliform cystidia present...............................................................................................................................58b. Clavate to spathuliform cystidia absent.....................................................................................................................................5958a. Hymenophore greyish-white or pale cream; basidiospores thick-walled, more than 4 µm wide.................*X. pruinosus*b. Hymenophore cinnamon to orange-brown; basidiospores thin-walled, less than 4 µm wide......................*X. yunnanensis*59a. Cylindrical gloeocystidia and subclavate cystidia.......................................................................................*X. subscopinellus*b. Leptocystidia present with encrustation.........................................................................................................*X. rhododendricola*60a. Tubular cystidia present..........................................................................................................................................................61b. Tubular cystidia absent................................................................................................................................................................6261a. Tubular cystidia thick-walled, slightly flexuous; basidia with two or rarely four sterigmata; basidiospores slightly thick-walled...............................................................................................................................................................*X. echinatus*b. Tubular tramacystidia thin-walled, snake-like sinuous; basidia with four sterigmata; basidiospores thin-walled...........................................................................................................................................................*X. subserpentiformis*62a. Pyriform cystidia present.........................................................................................................................................*X. pelliculae*b. Pyriform cystidia absent..............................................................................................................................................................6363a. Ventricose cystidia present............................................................................................................................*X. submucronatus*b. Ventricose cystidia absent...........................................................................................................................................................6464a. Basidiospores more than 6 μm long........................................................................................................................*X. lutescens*b. Basidiospores less than 6 μm long..............................................................................................................................................6565a. Basidiospores more than 3.5 µm wide...........................................................................................................................*X. rudis*b. Basidiospores less than 3.5 µm wide.........................................................................................................................*X. papillosus*66a. Hymenophore irpicoid or poroid...........................................................................................................................................67b. Hymenophore smooth, tuberculate or grandinioid.................................................................................................................8967a. Cystidia absent….....................................................................................................................................................*X. nongravis*b. Cystidia present............................................................................................................................................................................6868a. Moniliform or submoniliform cystidia present....................................................................................................................69b. Moniliform or submoniliform cystidia absent..........................................................................................................................7269a. Hymenophore irpicoid.............................................................................................................................................................70b. Hymenophore poroid..................................................................................................................................................................7170a. Hyphoid or subulate cystidia present mostly in aculei...............................................................................*X. anmashanensis*b. Hyphoid or subulate cystidia absent......................................................................................................................*X. spathulatus*71a. Basidiospores ellipsoid, less than 5.5 μm long.....................................................................................................*X. bresinskyi*b. Basidiospores suballantoid, more than 7.5 μm long..................................................................................................*X. syringae*72a. Basidiospores more than 6.5 μm long.....................................................................................................................................73b. Basidiospores less than 6.5 μm long...........................................................................................................................................7473a. Pores less than 1 mm in diam; cystidia capitate; basidiospores less than 8 µm long........................................*X. nothofagi*b. Pores more than 1 mm in diam; cystidia not capitate; basidiospores mostly more than 8 µm long.....................*X. syringae*74a. Basidia with two sterigmata...............................................................................................................................*X. crassihyphus*b. Basidia with four sterigmata.......................................................................................................................................................7575a. Hymenophore pileate; hyphal system trimitic..................................................................................................*X. trametoides*b. Hymenophore resupinate or effused-reflected; hyphal system monomitic to dimitic........................................................7676a. Subulate or cylindrical cystidia present.................................................................................................................................77b. Subulate or cylindrical cystidia absent.......................................................................................................................................7977a. Basidiospores broadly ellipsoid to subglobose, more than 4 μm wide........................................................*X. apacheriensis*b. Basidiospores ellipsoid, less than 4 μm wide............................................................................................................................7878a. Basidiospores more than 5 µm long........................................................................................................................*X. niemelaei*b. Basidiospores less than 5 µm long........................................................................................................................*X. subflaviporus*79a. Bottle-shaped cystidia present, apically with large rhomboid crystals...........................................................*X. cystidiatus*b. Bottle-shaped cystidia absent......................................................................................................................................................8080a. Hyphae without clamp connections.........................................................................................................*X. poroideoefibulatus*b. Hyphae with clamp connections................................................................................................................................................8181a. Capitate cystidia mostly bladder-like....................................................................................................................................82b. Capitate cystidia not bladder-like...............................................................................................................................................8382a. Basidia barrel-shaped to pyriform; basidiospores ellipsoid, less than 3.2 μm wide................................*X. pseudotropicus*b. Basidia utriform to clavate; basidiospores broadly ellipsoid, more than 3.2 µm wide....................................*X. mollissimus*83a. Basidiospores subglobose or ovoid........................................................................................................................................84b. Basidiospores ellipsoid................................................................................................................................................................8584a. Basidiospores subglobose, more than 4 µm wide.............................................................................................*X. hallenbergii*b. Basidiospores ovoid, less than 4 µm wide................................................................................................................*X. flaviporus*85a. Growth on palm or fern as substrate; capitate cystidia rare....................................................................................*X. gracilis*b. Growth on angiosperms or gymnosperms; capitate cystidia not rare...................................................................................8686a. Basidiospores more than 5.5 μm long...................................................................................................................*X. paradoxus*b. Basidiospores less than 5.5 μm long...........................................................................................................................................8787a. Hymenophore cream...........................................................................................................................................*X. taiwanianus*b. Hymenophore orange, rose or buff............................................................................................................................................8888a. Hymenophore beige to slightly orange; capitate cystidia not encrusted.......................................................*X. subtropicus*b. Hymenophore cream, pinkish cream or buff; capitate cystidia partly encrusted with brownish yellow resinous material......................................................................................................................................................................*X. ovisporus*89a. Basidia with two sterigmata......................................................................................................................................*X. bisporus*b. Basidia with four sterigmata.......................................................................................................................................................9090a. Capitate or subcapitate cystidia present................................................................................................................................91b. Capitate or subcapitate cystidia absent....................................................................................................................................10691a. Spores globose to subglobose..................................................................................................................................................92b. Spores cylindrical, ellipsoid to broadly ellipsoid......................................................................................................................9492a. Hymenophore smooth..............................................................................................................................................*X. pumilius*b. Hymenophore grandinioid.........................................................................................................................................................9393a. Capitate cystidia rare; basidiospores less than 4.5 μm long...................................................................................*X. tenellus*b. Capitate cystidia not rare; basidiospores more than 4.5 μm long..................................................................*X. hyphodontinus*94a. Gloeocystidia present..........................................................................................................................................*X. tuberculatus*b. Gloeocystidia absent....................................................................................................................................................................9595a. Tramacystidia present...........................................................................................................................................*X. verecundus*b. Tramacystidia absent...................................................................................................................................................................9696a. Basidiospores thick-walled......................................................................................................................................................97b. Basidiospores thin-walled.........................................................................................................................................................10097a. Basidiospores less than 3.5 µm wide.............................................................................................................................*X. rickii*b. Basidiospores more than 3.5 µm wide.......................................................................................................................................9898a. Hymenophore smooth..............................................................................................................................................*X. pumilius*b. Hymenophore grandinioid.........................................................................................................................................................9999a. Astrocystidia and submoniliform cystidia present, septocystidia absent......................................................*X. ussuriensis*b. Astrocystidia and submoniliform cystidia absent, septocystidia present..................................................*X. septocystidiatus*100a. Only capitate cystidia present.............................................................................................................................................101b. Besides capitate cystidia, other types of cystidia present......................................................................................................102101a. Hyphae not encrusted; cystidia with exudation; basidiospores cylindrical to subcylindrical................*X. rimosissimus*b. Hyphae sometimes encrusted; cystidia without exudation; basidiospores ellipsoid.........................................*X. yarraensis*102a. Basidiospores less than 3.5 μm wide..................................................................................................................................103b. Basidiospores more than 3.5 μm wide.....................................................................................................................................105103a. Cystidia not encrusted................................................................................................................................*X. heterocystidiatus*b. Cystidia strongly encrusted......................................................................................................................................................104104a. Basidiospores broadly ellipsoid, less than 5 µm long.........................................................................................*X. erikssonii*b. Basidiospores cylindrical to subcylindrical, more than 5 µm long......................................................................*X. gamundiae*105a. Hyphoid cystidia absent, lageniform, clavate and cylindrical cystidia present...........................................*X. hjortstamii*b. Hyphoid cystidia present, lageniform, clavate and cylindrical cystidia absent.................................................*X. attenuatus*106a. Gloeocystidia present........................................................................................................................................*X. tuberculatus*b. Gloeocystidia absent..................................................................................................................................................................107107a. Basidiospores globose to subglobose.................................................................................................................................108b. Basidiospores ellipsoid or cylindrical......................................................................................................................................109108a. Subulate cystidia or hyphal endings present, leptocystidia absent; basidiospores more than 5 µm long, more than 3.8 µm wide...................................................................................................................................................*X. crustosoglobosus*b. Subulate cystidia or hyphal endings absent, leptocystidia present; basidiospores more than 5 µm long, more than 3.8 µm wide..................................................................................................................................................................*X. victoriensis*109a. Cystidia absent......................................................................................................................................................................110b. Cystidia present..........................................................................................................................................................................112110a. Cystidioles absent...............................................................................................................................................*X. acystidiatus*b. Cystidioles present.....................................................................................................................................................................111111a. Basidiospores less than 5 µm long.......................................................................................................................*X. papillosus*b. Basidiospores more than 5 µm long....................................................................................................................*X. tenuicystidius*112a. Snake-like sinuous tubular tramacystidia present...........................................................................................................113b. Tubular tramacystidia absent...................................................................................................................................................114113a. Hymenial hyphae encrusted..........................................................................................................................*X. serpentiformis*b. Hymenial hyphae not encrusted...................................................................................................................*X. subserpentiformis*114a. Subulate cystidia present........................................................................................................................................*X. australis*b. Subulate cystidia absent.............................................................................................................................................................115115a. Basidiospores more than 4 µm wide....................................................................................................................*X. pruinosus*b. Basidiospores less than 4 µm wide...........................................................................................................................................116116a. Clavate-sinuous to submoniliform cystidia present; basidiospores narrowly ellipsoid.....................*X. damansaraensis*b. Clavate-sinuous to submoniliform cystidia absent, basidiospores ellipsoid to broadly ellipsoid...................................117117a. Leptocystidia and lagenocystidia absent, cylindrical to clavate cystidia and astrocystidia present............*X. detriticus*b. Leptocystidia and lagenocystidia present, cylindrical to clavate cystidia and astrocystidia absent......*X. lagenicystidiatus*

## 5. Discussion

Even though species diversity of *Hyphodontia* sensu lato has been extensively explored worldwide, the phylogenetic relationships among genera of *Hyphodontia* sensu lato and the phylogenetic positions of these genera within *Hymenochaetales* were unclear. In this study, the most comprehensive phylogenetic analyses to date focusing on genera belonging to *Hyphodontia* sensu lato were performed. The analyses resulted in segregation of these genera at the family level, viz. two previous names *Chaetoporellaceae* and *Schizoporaceae* and a newly proposed name *Hyphodontiaceae*, and placed all these families within *Hymenochaetales*. *Schizoporaceae* includes *Fasciodontia*, *Lyomyces* and *Xylodon*, while the monotypic families *Chaetoporellaceae* and *Hyphodontiaceae*, respectively, accommodate *Kneiffiella* and *Hyphodontia*. Noteworthily, the genus *Hastodontia* was excluded from these three families and treated as a genus of uncertain placement at the family level within *Hymenochaetales*.

*Hymenochaetales* was erected as a monotypic order on the basis of *Hymenochaetaceae* [90]. An additional family *Schizoporaceae* was also accommodated in *Hymenochaetales* by Larsson [8] and recognized in the latest edition of the Dictionary of the Fungi [45]. Later, *Oxyporaceae*, *Neoantrodiellaceae* and *Nigrofomitaceae* were successively delimited within *Hymenochaetales* [91,92,93]. Besides these five families, four additional families have been accepted in *Hymenochaetales* by some authors: *Coltriciaceae*, *Rickenellaceae*, *Repetobasidiaceae* and *Tubulicrinaceae*.

*Coltriciaceae* was typified by *Coltricia* and also included *Aurificaria*, *Coltriciella*, *Cyclomyces*, *Inonotus*, *Onnia* and *Phylloporia* as exemplified genera [2]. Most of taxonomists treated these genera in *Hymenochaetaceae*, which makes *Coltriciaceae* as a synonym of *Hymenochaetaceae* [46,94]. However, a few studies indicated that *Coltricia* and *Coltriciella* were independent from *Hymenochaetaceae* [7]. The current study agrees with the topology of Larsson et al. [7]: the clade including *Hymenochaetaceae* and *Coltriciaceae* did not receive reliable statistical support (Figure 2). Therefore, we accept *Coltriciaceae* for *Coltricia* and *Coltriciella* as an independent family from *Hymenochaetaceae*.

A recently published outline of *Basidiomycota* accepted *Rickenellaceae* in *Hymenochaetales* [46], which was followed by a subsequent outline of *Fungi* and fungus-like taxa [95]. However, *Rickenellaceae* is a nomenclaturally superfluous name, because its original circumscription includes *Repetobasidium*, the type genus of a prior family *Repetobasidiaceae*. According to Art. 52.4 of the Shenzhen Code [96], *Rickenellaceae* could be legitimate only if it no longer includes *Repetobasidium*. The concept of *Rickenellaceae* was used under the name ‘*Rickenella* family’ by Larsson [8] and Ariyawansa et al. [92], but its circumscription was polyphyletic. Korotkin et al. [97] used the name ‘*Rickenella* clade’ for species in *Rickenella* and 13 additional genera falling within a clade that was poorly supported. In the current phylogenetic analysis (Figure 2), two species of *Rickenella* grouped together with six of the above-mentioned 13 genera in an also poorly supported clade, while the other included genera out of the above-mentioned 13 genera were separated from this clade. Regarding *Repetobasidiaceae*, since its erection in 1981, it does not appear to have been considered a family of *Hymenochaetales*, even though species of its type genus *Repetobasidium* were phylogenetically included in *Hymenochaetales* [7]. This may be due to the absence of the generic type *Repetobasidium vile* in any phylogenetic analysis. Therefore, the circumscription of *Repetobasidiaceae* is still ambiguous from a phylogenetic perspective. *Tubulicrinaceae* erected with *Tubulicrinis* as the type genus [2] was another family accepted at one time in *Hymenochaetales* [8]. The clade labeled as *Tubulicrinaceae* with three species from two additional genera *Hyphodontia* and *Sphaerobasidium*, besides three species of *Tubulicrinis*, however, received no strong statistical support [8]. In addition, *Tubulicrinis* was shown to be polyphyletic with two species of *Tubulicrinis* distantly related to the so-called *Tubulicrinaceae* clade [7], and more importantly its type species *T. glebulosus* has not been included in any phylogenetic analysis. Another preliminary phylogenetic analysis on *Hymenochaetales* used *Tubulicrinaceae* for the clade including *Athelopsis lunata* (currently accepted as *Sidera lunata*) [98], *Sphaerobasidium minutum* and four species of *Tubulicrinis*, which received low statistical supports [92]. In the current phylogeny, the weakly supported clade including three species of *Tubulicrinis* and one of *Sphaerobasidium* was separated from *Sidera lunata*, and within this clade, the three species of *Tubulicrinis* did not cluster together (Figure 2). Alternatively, *Tubulicrinaceae* was not accepted in *Hymenochaetales*, but treated as a later synonym of Hydnaceae by the latest edition of the Dictionary of the Fungi [45] and the recent outline of *Basidiomycota* [46]. It is not possible to apply the family names *Rickenellaceae*, *Repetobasidiaceae* and *Tubulicrinaceae* with confidence at the moment. Undoubtfully, a wider sampling including the generic types of the respective families is needed to judge whether the families should be taken up for monophyletic clades within *Hymenochaetales*. Nevertheless, the current phylogeny excludes the possibility of the six genera belonging to *Hyphodontia* sensu lato having overlapping circumscriptions with these three families at the family level (Figure 2).

Taking all this into account, after an arrangement of *Hyphodontia* sensu lato into three families, eight families are accepted in *Hymenochaetales*, viz. *Chaetoporellaceae*, *Coltriciaceae*, *Hymenochaetaceae*, *Hyphodontiaceae*, *Neoantrodiellaceae*, *Nigrofomitaceae*, *Oxyporaceae* and *Schizoporaceae*.

Zhao et al. [99] proposed that the taxonomic units at the same rank, especially higher ranks, should have roughly equivalent stem ages, and overlap between the ranges of different ranks should be minimized. This approach was also applied by He et al. [46]. Lücking [100] provides a critique, arguing that ‘temporal banding should not be employed in an absolute manner, but rather as a tool complementing assessments of phenotypic disparity and differential diagnoses at given rank levels’. Except those for fossil fungi, almost all taxa in the current taxonomic system of fungi are introduced mainly for extant species (crown groups), but not unknown and extinct species (stem groups). For example, in the current case, *Hyphodontiaceae* is newly introduced to accommodate species of *Hyphodontia*. Moreover, excluding mass extinction events that are not known for fungi, the crown group emerges just before the extinction of the stem group [101], which indicates that the crown age is much closer to the genuine emergence time of the crown group than the stem age. Therefore, we use the crown age instead of the stem age, similar to Liu et al. [102], for complementary discussion of families within *Hymenochaetales* in the current study. The current estimations suggest that *Chaetoporellaceae* and *Hyphodontiaceae* share close crown age ranges, while those of *Schizoporaceae*, *Hymenochaetaceae* and *Oxyporaceae* are overlapping and somewhat older (Figure 6). However, compared with these five families, *Coltriciaceae*, *Neoantrodiellaceae* and *Nigrofomitaceae* emerged considerably later (Figure 6). Interestingly, *Coltriciaceae*, *Neoantrodiellaceae* and *Nigrofomitaceae* as well as *Hymenochaetaceae* formed a strongly supported clade together with the three genera *Basidioradulum*, *Fibricium* and *Trichaptum* with uncertain taxonomic positions at the family rank in the phylogeny based on a combined dataset of ITS, nLSU and mt-SSU regions (Figure 2). The clade including these four families emerged in the mean crown age of 191.36 Mya with a 95% HPD of 173.9-208.63 Mya, which, compared with those of *Coltriciaceae*, *Neoantrodiellaceae* and *Nigrofomitaceae*, was much closer to the crown ages of other four families in *Hymenochaetales* (Figure 6). The phylogeny and the much younger crown ages for these three families both indicate that a larger concept of *Hymenochaetaceae* (inclusive of *Coltriciaceae*, *Neoantrodiellaceae* and *Nigrofomitaceae*) is worth considering. Further comprehensive phylogenetic analyses focusing on this clade with a wider taxon sampling will be helpful to generate a reliable phylogenetic resolution for the circumscription of *Hymenochaetaceae*.

*Oxyporaceae* was originally a monotypic family typified by *Oxyporus* [91]. *Leucophellinus* was subsequently added to this family [92]. Later, the type genus of *Oxyporaceae* was considered to be a later synonym of *Rigidoporus*, and species of *Oxyporus* were transferred to this genus as well as to *Bridgeoporus* in *Hymenochaetales* [103]. According to the current phylogeny based on three genes (Figure 2), *Bridgeoporus*, *Leucophellinus* and *Rigidoporus* are all accepted in *Oxyporaceae*. Notably, *Oxyporaceae* did not receive a strongly supported statistical value from the ML algorithm (BS = 72%), although it received a full statistical value from the BI algorithm (Figure 2). Like *Hymenochaetaceae*, further research is required to confirm the circumscription of *Oxyporaceae*.

Besides the eight accepted families, about twenty genera have no available taxonomic position at the family rank in *Hymenochaetales*, partly due to a lack of systematic surveys on their species diversity. In developing a comprehensive classification of *Hymenochaetales*, more taxonomic work on species diversity and phylogeny of these poorly known genera needs to be performed.

As one of the most obvious traits of basidiomycetous fungi, basidiocarps play an essential role in the process of sexual reproduction via protecting reproductive organs and promoting basidiospore dispersal [104]. Previous studies at a higher taxonomic scale (at or above the order level) suggested that the ancestral shape of basidiocarps was resupinate and evolved several times to pileate-stipitate shape [105,106,107]. While this higher taxonomic scale provided a framework for the evolution of basidiocarp shape, the situation within certain orders has rarely been explored. Moreover, besides basidiocarp shape, the hymenophoral configuration is also crucial for sexual reproduction. It is common sense that, for example, poroid hymenia can provide stronger protection for reproductive organs but also an obstacle for basidiospore dispersal in comparison with smooth hymenia. Ancestral state reconstruction on such characters does not appear to have been performed. Species in *Hymenochaetales* have diverse traits of basidiocarps. This means that now that a phylogenetic framework for *Hymenochaetales* has been generated with an emphasis on a wide sampling of species belonging to *Hyphodontia* sensu lato, it is possible to explore for the first time the trait evolution of basidiocarps from the perspectives of basidiocarp shape and hymenophoral configuration simultaneously (Figure 7). The current result indicates that the evolutionary direction of basidiocarp shape below the order level is generally consistent with that at or above the order level (from resupinate to pileate). Meanwhile, neither smooth nor poroid hymenia, but the intermediate character (grandinioid and odontioid hymenia) perhaps representing a balance between protection and dispersal appears to have the adaptive advantage at least in certain lineages, where it has evolved independently. Interestingly, after evolving to the grandinioid state from the poroid state, the hymenophoral configuration in *Hymenochaetaceae* and *Neoantrodiellaceae* evolved back to the poroid state. This evolutionary event coincided with the evolution of basidiocarp shape from resupinate to pileate habit. However, in the lineage indicated by a pentagram mark (Figure 7), another evolutionary event occurred with a transition back to the poroid state from the grandinioid state, and this transition did not correspond to a transition from the resupinate to pileate habit. In *Oxyporaceae*, the ancestral state of basidiocarps was retained, resupinate habit and poroid hymenophoral configuration. So, a relationship between the pileate and poroid states of basidiocarps is indicated but is not strict. These findings provide a number of candidates for genomic analysis in a phylogenetic framework to investigate the evolutionary dynamics of transitions in basidiocarp shape and hymenophoral configuration, especially as far as reversals of hymenophoral configuration.

## Data Availability

Publicly available datasets were analyzed in this study. All resulting alignments were deposited in TreeBASE (http://www.treebase.org; accession number S27307). All newly generated sequences were deposited in GenBank (https://www.ncbi.nlm.nih.gov/genbank/; Table S1). All new taxa were deposited in Index Fungorum (http://www.indexfungorum.org/Names/IndexFungorumRegisterName.asp; Index Fungorum identifiers follow new taxa).

## Supplementary Materials

The following are available online at https://www.mdpi.com/article/10.3390/jof7060478/s1. Table S1: Information of taxa used in phylogenetic analyses; Table S2: Information of species used in molecular clock analysis besides those indicated in Table S1.
