# Supplementary material for: Towards a Natural Classification of Hyphodontia Sensu Lato and the Trait Evolution of Basidiocarps within Hymenochaetales (Basidiomycota)
Source: J Fungi (Basel). 2021 Jun 12;7(6):478. doi: 10.3390/jof7060478 (PMC8231612; doi:10.3390/jof7060478)
Supplement: Supplementary file 1 [file jof-07-00478-s001.zip › Table S1.pdf]

Table S1 Information of taxa used in phylogenetic analyses

Order/family	Species	Voucher	ITS	LSU	mt-SSU	*tef1α*	*rpb1*	*rpb2*	*atp6*
Auriculariales/Auriculariaceae	*Auricularia cornea*	LWZ 20190421-1 (HMAS)*	**MT319406**	**MT319135**	**MT326526**				
Hymenochaetales/Chaetoporellaceae	*Kneiffiella abdita*	Dai 15045	**MW209700**		**MW221940**				
	K. abieticola	GEL 2924	DQ340331						
		KHL 12498[#]	DQ873601	DQ873601					
		UC2022838	KP814342						
	K. alienata	CBS 127219	MH864327	MH875772					
		EL 14-98	AY466401	AY586727					
	K. alutacea	GEL 2284	DQ340340						
		GEL 2937	DQ340338						
	K. barba-jovis	KHL 11730[#]	DQ873609	DQ873610					
	K. cineracea	GEL 4875	DQ340335						
		GEL 4958	DQ340336						
	K. decorticans	SP 415980	KY081795						
	K. efibulata	GB-0151167	KY081796						
	K. eucalypticola	LWZ 20180509-11 (HMAS)[#]*	**MT319410**	**MT319142**	**MT326421**				
		LWZ 20180512-7 (HMAS)	**MT319409**	**MT319141**	**MT326420**				
		LWZ 20180515-9 (HMAS)	**MT319411**	**MT319143**					
	K. floccosa	Berglund 150-02	DQ873618	DQ873618					
		UC2022896	KP814440						

		UC2022902	KP814441					
		UC2022919	KP814153					
	K. microspora	Hjortstam 18578	KY081792					
	= *K. palmae*	FR7	KP689185					
		GEL 3456	DQ340333					
	K. pilaecystidiata	Cui 13239	KY290984					
		Cui 13240	KY290985					
		MSK-F 4723	MK575208	MK598739				
	K. stereicola	Blackwell 2141	KY081797					
	K. subalutacea	GEL 2142	DQ340334					
		GEL 2196	DQ340341					
		KUC 20130726-11	KJ668514					
		LWZ 20170816-9 (IFP)#	**MT319407**	**MT319139**				
		UC2023092	KP814444					
	K. subefibulata	Dai 10526	KT989970					
		Dai 10803	KT989971					
	K. subglobosa	LWZ 20180416-6 (HMAS)*	**MT319413**	**MT319145**	**MT326422**			
		Wu 890805-2	KY081798					
	Kneiffiella sp.	LWZ 20171015-2 (IFP)#*	**MT319412**	**MT319144**	**MT326527**			
	Kneiffiella sp.	LWZ 20180510-31 (HMAS)*	**MT319408**	**MT319140**	**MT326419**			
/*Coltriciaceae*	*Coltricia perennis*	Cui 10319#	KU360687	KU360653		KY693935	KX364848	KX364894
	Coltriciella	Dai 10944#	KY693737	KY693757				KY693908

	dependens				
/*Hymenochaetaceae*	*Fomitiporia hartigii*	Cui 9914 (BJFC)*	KY750527	**MT319381**	**MT326559**
	Fulvoderma scaurum	LWZ 20170816-31 (IFP)*	**MT332135**	**MT319395**	**MT326509**
	Fuscoporia viticola	LWZ 20130510-3 (IFP)	**MT332136**	**MT319382**	
	Hymenochaete rubiginosa	He 1049#*	JQ716407	JQ279667	KU978871
	Inonotus hispidus	LWZ 20180703-1 (HMAS)*	**MT332137**	**MT319383**	**MT326506**
	Mensularia radiata	Yuan 6589 (IFP)*	**MT332140**	**MT319386**	**MT326560**
	Onnia tomentosa	Cui 10048 (BJFC)*	**MT332141**	**MT319387**	**MT326561**
	Phellinidium ferrugineofuscum	Cui 10042 (BJFC)*	KR350573	**MT319388**	**MT326562**
	Phellinus piceicola	Cui 10440 (BJFC)*	JQ828908	**MT319389**	**MT326563**
	Porodaedalea pini	Dai 7624 (IFP)*	**MT332142**	**MT319391**	**MT326565**
	Tropicoporus boehmeriae	LWZ 20140729-10 (IFP)	KT223640	**MT319393**	
/*Hyphodontiaceae*	*Hyphodontia alutaria*	KHL 11889	DQ873603	DQ873603	
		KHL 11978	EU118631		
		UC2023078	KP814431		
	H. arguta	FR-0219450	KR349242		
		FR-0219451	KR349243		
	H. borbonica	FR-0219441	KR349240		
		FR-0219444	KR349241		

H. densispora	LWZ 20170908-5 (IFP)#*	**MT319426**	**MT319160**	**MT326431**		**MT326350**	**MT326261**
	LWZ 20180905-6 (HMAS)	**MT319425**	**MT319159**	**MT326430**			
'*H. arguta*'	Hjm 18726	DQ873605	DQ873605				
H. pallidula	GEL 2097	DQ340317					
H. subdetritica	FR-0261085	KY081793					
	FR-0261087	KY081794					
H. wongiae	LWZ 20180414-16 (HMAS)	**MT319414**	**MT319146**				
	LWZ 20180417-8 (HMAS)	**MT319415**					
	LWZ 20180417-16 (HMAS)	**MT319416**	**MT319147**				
H. zhixiangii	LWZ 20160909-4 (IFP)	KY440396	**MT319154**	**MT326426**			
	LWZ 20160909-8 (IFP)	KY440397	**MT319155**	**MT326427**			
	LWZ 20160909-9 (IFP)	KY440398	**MT319156**	**MT326428**			
	LWZ 20170818-13 (IFP)#*	**MT319420**	**MT319151**	**MT326424**	**MT326397**	**MT326361**	**MT326270**
	LWZ 20170820-27 (IFP)	**MT319421**	**MT319152**	**MT326425**	**MT326408**		
	LWZ 20170820-31 (IFP)	**MT319422**	**MT319153**				

		LWZ 20180903-5 (HMAS)	**MT319423**	**MT319158**	**MT326429**	**MT326398**		**MT326294**
		LWZ 20180903-9 (HMAS)	**MT319424**	**MT319157**				
		LWZ 20180904-12 (HMAS)	**MT319419**	**MT319150**				
	Hyphodontia sp.	LWZ 20170814-15 (IFP)#*	**MT319417**	**MT319148**	**MT326423**			**MT326269**
	Hyphodontia sp.	LWZ 20180511-2 (HMAS)	**MT319418**	**MT319149**				
/Neoantrodiellaceae	*Neoantrodiella gypsea*	Cui 10372#*	KT203290	**MT319396**	**MT326567**			
	N. thujae	Dai 5065#	KT203293	**MT319397**	**MT326568**			
/Nigrofomitaceae	*Nigrofomes melanoporus*	JV 1704/39#	MF629835	MF629831				
	N. sinomelanoporus	Cui 5277#	MF629836	**MT319398**				
/Oxyporaceae	*Bridgeoporus sinensis*	Cui 10013#	KY131832	KY131891				
	Leucophellinus hobsonii	Cui 6468#*	KT203288	KT203309	KT203330			
	L. irpicoides	Yuan 2690*	KT203289	KT203310	KT203331			
	Rigidoporus corticola	Dai 12632*	KF111018	KF111020	KT203334			
	R. cuneatus	Dai 7339*	KT203294	KT203315	KT203335			
	R. ginkgonis	Cui 5555#*	KT203295	KT203316	KT203336			
	R. macroporus	Dai 4044	KT203298	KT203319				

	R. obducens	Dai 6451	KY131884	KY131941	
	R. philadelphi	Wei 1622*	KT203300	KT203321	KT203340
	R. piceicola	Dai 5033*	KT203301	KT203322	KT203341
	R. populinus	Dai 8908*	KY131887	KY131944	KT203342
	R. subpopulinus	Cui 2236*	KT203305	KT203326	KT203346
/*Schizoporaceae*	*Fasciodontia brasiliensis*	MSK-F 7245a	MK575201	MK598734	
	F. bugellensis	MSK-F 5548	MK575204	MK598736	
	Fasciodontia sp.	KUC 20121109-15	KJ668516	KJ668368	
	Lyomyces allantosporus	FR-0249548	KY800397		
		GEL 4933	KY800401		
	L. bambusinus	CLZhao 3675	MN945969		
		CLZhao 4808	MN945970		
		CLZhao 4831	MN945968		
		CLZhao 4840	MN945971		
	L. cremeus	CLZhao 2812	MN945973		
		CLZhao 4138	MN945974		
		CLZhao 8295	MN945972		
	L. crustosus	CFMR 4572	KX065944		
		CLZhao 875	MH114730		
		CLZhao 1157	MG231629		
		GEL 2325	DQ340313		
		GEL 5336	DQ340314		
		GEL 5360	DQ340315		
		HHB 13100	MH260069		

KHL 11731	DQ873614	DQ873614					
KUC 20121109-18	KJ668519						
LWZ 20170814-22 (IFP)#*	**MT319464**	**MT319200**	**MT326445**		**MT326360**	**MT326293**	**MT326570**
LWZ 20170815-23 (IFP)	**MT319465**	**MT319201**	**MT326446**	**MT326400**	**MT326313**	**MT326275**	**MT326571**
LWZ 20170815-32 (IFP)	**MT319466**	**MT319202**	**MT326447**	**MT326402**	**MT326314**	**MT326276**	**MT326572**
LWZ 20170815-46 (IFP)	**MT319467**			**MT326401**	**MT326355**		**MT326574**
LWZ 20170815-53 (IFP)	**MT319670**	**MT319209**					
LWZ 20170816-1 (IFP)	**MT319671**	**MT319208**					
LWZ 20170816-3 (IFP)	**MT319472**	**MT319207**	**MT326451**	**MT326410**	**MT326315**		**MT326575**
LWZ 20170816-20 (IFP)	**MT319463**	**MT319199**	**MT326524**				**MT326573**
LWZ 20180830-7 (HMAS)	**MT319468**	**MT319203**					
LWZ 20180904-2 (HMAS)	**MT319469**	**MT319205**	**MT326449**				
LWZ 20180904-11 (HMAS)	**MT319470**	**MT319206**	**MT326450**	**MT326393**	**MT326316**		
LWZ 20180904-31 (HMAS)	**MT319471**	**MT319204**	**MT326448**		**MT326317**		

	UC2022841	KP814310		
	Wu 0711-90	KC460312		
	Wu 1007-31	KC460313		
	YG-G39	MF382993		
'*L. juniperi*'	Wu 0308-26	KC460311		
L. elaeidicola	LWZ 20180411-2 (HMAS)	**MT319454**	**MT319193**	
	LWZ 20180411-17 (HMAS)	**MT319455**	**MT319191**	
	LWZ 20180411-18 (HMAS)	**MT319456**	**MT319189**	
	LWZ 20180411-19 (HMAS)	**MT319457**	**MT319190**	
	LWZ 20180411-20 (HMAS)*	**MT319458**	**MT319192**	**MT326442**
L. erastii	30SAMHYP	JX857796		
	32bSAMHYP	JX857798		
	36SAMHYP	JX857797		
	LWZ 20160907-5 (IFP)	**MT319453**	**MT319188**	
	MA-Fungi 34336	JX857800		
	YG 022	MF382992		
L. fimbriatus	Wu 911204-4	MK575210	MK598740	
L. gatesiae	LWZ 20180515-3 (HMAS)	**MT319447**	**MT319181**	
	LWZ 20180515-32	**MT319448**	**MT319182**	

	(HMAS)							
L. griseliniae	KHL 12971	DQ873651	DQ873651					
L. juniperi	GEL 4940	DQ340316						
	Wu 0910-95	JX175047						
L. leptocystidiatus	LWZ 20170814-8 (IFP)	**MT319432**	**MT319166**					
	LWZ 20170814-14 (IFP)#*	**MT319429**	**MT319163**	**MT326512**	**MT326395**	**MT326341**	**MT326256**	**MT326576**
	LWZ 20170815-2 (IFP)	**MT319430**	**MT319164**	**MT326513**			**MT326257**	**MT326577**
	LWZ 20170815-30 (IFP)	**MT319427**	**MT319161**	**MT326510**		**MT326345**	**MT326259**	**MT326581**
	LWZ 20170815-43 (IFP)	**MT319428**	**MT319162**	**MT326511**	**MT326409**	**MT326343**	**MT326260**	**MT326582**
	LWZ 20170818-1 (IFP)	**MT319431**	**MT319165**	**MT326514**		**MT326342**		**MT326579**
	LWZ 20170818-8 (IFP)	**MT319433**	**MT319167**			**MT326344**	**MT326258**	**MT326580**
	LWZ 20170818-9 (IFP)	**MT319435**	**MT319169**		**MT326396**	**MT326346**	**MT326292**	**MT326578**
	LWZ 20170908-14 (IFP)	**MT319434**	**MT319168**	**MT326528**				
L. macrosporus	CLZhao 3951	MN945976						
	CLZhao 4516	MN945977						
	CLZhao 4531	MN945978						
	CLZhao 8605	MN945975						

	LWZ 20170817-2 (IFP)[#]	**MT319459**	**MT319194**		**MT326392**	**MT326349**		
	LWZ 20180922-1 (HMAS)	**MT319656**	**MT319195**					
L. mascarensis	GEL 4833	KY800399						
	GEL 4908	KY800400						
L. microfasciculatus	CLZhao 4626	MK343568						
	CLZhao 5109	MN954311						
	GN 2370 (BJFC)	**MT333854**						
	LWZ 20170815-37 (IFP)[#]*	**MT319450**	**MT319184**	**MT326440**		**MT326356**		**MT326583**
	LWZ 20170816-33a (IFP)	**MT319449**	**MT319183**	**MT326439**	**MT326386**			**MT326585**
	LWZ 20170820-18 (IFP)	**MT319451**	**MT319185**	**MT326441**	**MT326387**	**MT326347**	**MT326264**	**MT326584**
	LWZ 20180414-6 (HMAS)	**MT319452**	**MT319186**					
	LWZ 20180921-22 (HMAS)	**MT319654**	**MT319187**					
	TNM F24757	JN129976						
L. orientalis	GEL 3376	DQ340325						
	GEL 3400	DQ340326						
	LWZ 20170909-7 (IFP)*	**MT319436**	**MT319170**	**MT326529**				
L. organensis	MSK 7247	KY800403						
L. pruni	Ryberg 021018	DQ873624	DQ873625					

L. sambuci	18SAMHYP	JX857731					
	80SAMHYP	JX857721					
	83SAMHYP	JX857720					
	112SAMHYP	JX857715					
	125SAMHYP	JX857704					
	174SAMHYP	JX857734					
	176SAMHYP	JX857733					
	JR 7	KY800402					
	LWZ 20170908-13 (IFP)	**MT319445**	**MT319179**	**MT326530**			
	LWZ 20180903-4 (HMAS)	**MT319442**	**MT319176**	**MT326436**	**MT326411**		**MT326286**
	LWZ 20180903-11 (HMAS)	**MT319440**	**MT319174**	**MT326434**	**MT326388**		
	LWZ 20180905-1 (HMAS)	**MT319444**	**MT319178**	**MT326438**	**MT326391**		**MT326291**
	LWZ 20180905-2 (HMAS)	**MT319438**	**MT319172**	**MT326433**	**MT326389**		**MT326287**
	LWZ 20180905-7 (HMAS)[#]	**MT319439**	**MT319173**			**MT326348**	
	LWZ 20180905-8 (HMAS)*	**MT319441**	**MT319175**	**MT326435**	**MT326390**		**MT326288**
	LWZ 20180906-7 (HMAS)	**MT319443**	**MT319177**	**MT326437**			**MT326290**
	LWZ 20180906-10 (HMAS)	**MT319437**	**MT319171**	**MT326432**	**MT326412**		**MT326289**

	r112SAMHYP	JX857716				
	TAAM 104263	MF382989				
L. cf. *sambuci*	DLL 2011-052	KJ140574				
L. cf. *sambuci*	DLL 2011-304	KJ140776				
L. stratosus	LR 9798	KY081805				
	LR 10025	KY081806				
L. vietnamensis	TNM F9073	JX175044				
L. wuliangshanensis	CLZhao 4108	MN945980				
	CLZhao 4144	MN945981				
	CLZhao 4167	MN945979				
	CLZhao 4206	MN945982				
	CLZhao 4475	MN945983				
Lyomyces sp.	170SAMHYP	JX857770				
	GEL 2414	KY800398				
Lyomyces sp.	LWZ 20180512-6 (HMAS)	**MT319446**	**MT319180**			
Lyomyces sp.	LWZ 20180515-2 (HMAS)*	**MT319460**	**MT319196**	**MT326443**		
Lyomyces sp.	LWZ 20180515-17 (HMAS)	**MT319461**	**MT319197**			
Lyomyces sp.	LWZ 20180906-20 (HMAS)[#]*	**MT319462**	**MT319198**	**MT326444**		**MT326318**
Xylodon acystidiatus	LWZ 20180514-9 (HMAS)	**MT319474**	**MT319211**			
	LWZ 20180515-35 (HMAS)	**MT319473**	**MT319210**			

X. apacheriensis	Canfield 180	KY081800		
X. asperus	CLZhao 1168	MG231625		
	CLZhao 1070	MG231621		
	CLZhao 1076	MG231622		
	CLZhao 1183	MG231627		
	GN2418 (BJFC)	**MT333855**		
	KG Nilsson s. n.	DQ873606	DQ873607	
	UC2023169	KP814365		
X. astrocystidiatus	Wu 9211-71	JN129972	JN129973	
X. attenuatus	Spirin 8775	MH324476		
X. australis	LWZ 20180509-8 (HMAS)	**MT319503**	**MT319248**	
	LWZ 20180509-15 (HMAS)*	**MT319500**	**MT319246**	**MT326519**
	LWZ 20180509-20 (HMAS)	**MT319501**	**MT319245**	**MT326457**
	LWZ 20180510-26 (HMAS)	**MT319504**	**MT319250**	**MT326459**
	LWZ 20180512-14 (HMAS)	**MT319502**	**MT319247**	**MT326458**
	LWZ 20180513-6 (HMAS)	**MT319505**	**MT319249**	
X. borealis	Spirin 9416	MH317760	MH638259	
	UC2022850	KP814307		
X. brevisetus	KHL 12386	DQ873612	DQ873612	
	KUN 2352	MH307753		

	UC2023199	KP814485					
X. cf. *brevisetus*	UC2023212	KP814495					
	UC2023234	KP814497					
X. crystalliger	KUN 2312	MH324477					
	LWZ 20170815-7 (IFP)[#]*	**MT319520**	**MT319268**	**MT326467**	**MT326383**		**MT326273**
	LWZ 20170816-33 (IFP)	**MT319521**	**MT319269**	**MT326468**		**MT326334**	**MT326253**
X. cystidiatus	FR-0249200	MH880195	MH884896				
X. damansaraensis	LWZ 20180417-4 (HMAS)*	**MT319496**	**MT319241**	**MT326525**			
	LWZ 20180417-20 (HMAS)	**MT319497**	**MT319242**				
	LWZ 20180417-22 (HMAS)	**MT319498**	**MT319243**				
	LWZ 20180417-23 (HMAS)	**MT319499**	**MT319244**	**MT326456**			
X. detriticus	Zíbarová 26.05.17	MH320794	MH638264				
	Zíbarová 30.10.17	MH320793	MH651372				
X. filicinus	MSK-F 12869	MH880199	MH884899				
	MSK-F 12870	MH880200	MH884900				
X. flaviporus	CLZhao 2384	MH114732					
	Cui 11838 (BJFC)	**MT319544**	**MT319271**				
	Dai 9996 (IFP)	**MT319554**					
	Dai 12797 (BJFC)	**MT319555**	**MT319278**	**MT326541**	**MT326413**		**MT326310**
	Dai 12808 (BJFC)	**MT319551**	**MT319275**	**MT326540**	**MT326414**		**MT326311**

Dai 14651 (BJFC)	**MT319542**					
Dai 16248 (BJFC)	**MT319557**	**MT319280**				
DLL 2011-134	KJ140637					
FCUG 1053	AF145575					
FR-0249797	MH880201	MH884901				
GEL 3462	MH880202					
GEL 5047	MH880203					
ICMP 13836	AF145585					
JV 1808/40	**MT319522**	**MT319290**				
JV 1808/65	**MT319523**	**MT319291**				
JV 1808/137	**MT319524**	**MT319292**				
LWZ 20150707-8 (IFP)	**MT319561**	**MT319283**	**MT326542**			
LWZ 20150708-1 (IFP)	**MT319553**	**MT319277**	**MT326473**	**MT326405**	**MT326328**	**MT326252**
LWZ 20150726-10 (IFP)	**MT319560**	**MT319282**				
LWZ 20150826-1 (IFP)	**MT319548**	**MT319274**	**MT326472**		**MT326333**	
LWZ 20160318-1 (IFP)	**MT319558**	**MT319281**				
LWZ 20160318-6 (IFP)	**MT319559**			**MT326380**		
LWZ 20170814-23 (IFP)	**MT319543**	**MT319270**	**MT326469**	**MT326376**	**MT326331**	**MT326271**
LWZ 20170820-43	**MT319556**	**MT319279**	**MT326474**	**MT326377**	**MT326329**	**MT326272**

	(IFP)						
	LWZ 20171014-2 (IFP)	**MT319563**	**MT319284**	**MT326475**	**MT326381**		
	LWZ 20171026-1 (IFP)	**MT319546**	**MT319272**	**MT326470**	**MT326379**	**MT326330**	
	LWZ 20171026-11 (IFP)#*	**MT319547**	**MT319273**	**MT326471**	**MT326378**	**MT326332**	**MT326277**
	LWZ 20180415-13 (HMAS)	**MT319552**	**MT319276**				
	MA-Fungi 79440	MH260071	MH260066				
	Yuan 4271 (IFP)	**MT319550**					
	Yuan 4314 (IFP)	**MT319562**					
	Yuan 4541 (IFP)	**MT319549**					
	Yuan 4544 (IFP)	**MT319545**					
	Yuan 4588 (IFP)	**MT319564**					
‘*X. ovisporus*’	GC 1609-41	MF540764					
‘*X. ovisporus*’	Wu 910322	MF540765					
‘*X. ovisporus*’	Wu 1207-25	MF540766					
X. follis	FR-0249814	MH880204	MH884902				
X. hastifer	Ryvarden 19767	KY081801					
X. heterocystidiatus	LWZ 20171015-8 (IFP)	**MT319513**	**MT319263**	**MT326465**			
	LWZ 20171015-15 (IFP)	**MT319514**					
	LWZ 20171015-29 (IFP)	**MT319516**	**MT319262**	**MT326464**			**MT326278**

	LWZ 20171015-30 (IFP)*	**MT319517**	**MT319261**	**MT326463**		**MT326336**	
	LWZ 20171015-33 (IFP)[#]	**MT319518**	**MT319264**	**MT326466**	**MT326382**	**MT326337**	**MT326285**
	LWZ 20180921-19 (HMAS)	**MT319676**	**MT319266**				
	LWZ 20180922-45 (HMAS)	**MT319659**	**MT319265**	**MT326538**			
	Wu 9209-27	JX175045					
	Wu 9312-20	JX175046					
X. hyphodontinus	GEL 9222	MH880205	MH884903				
	GG-GUY13-044	MH880206	MH884904				
	GG-MAR12-238	MH880207	MH884905				
	GG-MAR15-127	MH880208	MH884906				
X. kunmingensis	CLZhao 230	MK404528					
	CLZhao 752	MK404529					
	CLZhao 755	MK404530					
	CLZhao 3010	MK404531					
	CLZhao 3019	MK404532					
	LWZ 20170820-41 (IFP)[#]*	**MT319512**	**MT319259**	**MT326462**	**MT326399**	**MT326335**	**MT326274**
	LWZ 20171026-14 (IFP)	**MT319511**	**MT319258**	**MT326461**			**MT326284**
= *X. exilis*	MSK-F 7381	MH880196					
	TUB-FO 42450	MH880197					
	TUB-FO 42565	MH880198	MH884898				

X. lagenicystidiatus	LWZ 20180513-16 (HMAS)	**MT319634**	**MT319368**	
	LWZ 20180515-1 (HMAS)	**MT319632**		
	LWZ 20180515-14 (HMAS)*	**MT319633**	**MT319367**	**MT326499**
X. lenis	Cui 5950	KT989972		
	Wu 0808-32	JX175043		
	Wu 890714-3	KY081802		
X. mollissimus	LWZ 20160318-3 (IFP)*	KY007517	**MT319347**	**MT326555**
	Yuan 4391 (IFP)	**MT319609**		
	Yuan 4562 (IFP)	**MT319610**		
	Yuan 4568 (IFP)	**MT319611**		
X. nesporii	B Nordén 030915	DQ873622	DQ873622	
	CLZhao 879	MH114737		
	CLZhao 898	MG231640		
	CLZhao 944	MH114738		
	CLZhao 958	MH114739		
	CLZhao 1181	MG231645		
	CLZhao 3988	MH114741		
	GEL 3290	DQ340309		
	GEL 3302	DQ340308		
	GEL 3309	DQ340307		
	JR 14	MH880210		
	KUC 20130712-14	KJ668518		

KUC 20161012-50	MF774797					
LWZ 20170815-13 (IFP)	**MT319661**	**MT319239**				
LWZ 20170815-13a (IFP)	**MT319662**	**MT319240**				
LWZ 20170909-9 (IFP)	**MT319495**	**MT319236**	**MT326531**	**MT326403**		
LWZ 20171016-12 (IFP)[#]*	**MT319493**	**MT319235**	**MT326455**	**MT326384**	**MT326353**	**MT326262**
LWZ 20171016-18 (IFP)	**MT319494**	**MT319234**	**MT326454**	**MT326385**	**MT326354**	**MT326263**
LWZ 20180509-4 (HMAS)	**MT319488**	**MT319229**				
LWZ 20180509-14 (HMAS)	**MT319492**	**MT319233**				
LWZ 20180514-1 (HMAS)	**MT319489**	**MT319230**				
LWZ 20180514-12 (HMAS)	**MT319490**	**MT319232**				
LWZ 20180514-13 (HMAS)	**MT319491**	**MT319231**				
LWZ 20180804-27 (HMAS)	**MT319647**	**MT319237**	**MT326532**			
LWZ 20180921-35 (HMAS)	**MT319655**	**MT319238**	**MT326533**			
LWZ 20180922-31	**MT319675**					

	(HMAS)						
X. niemelaei	Cui 11116 (BJFC)	**MT319631**					
	Dai 15358 (BJFC)	**MT319623**	**MT319359**				
	Dai 16269 (BJFC)	**MT319624**	**MT319360**				
	FR-0219860	MH880211					
	FR-0249174	MH880212					
	GC 1508-146	KX857798					
	GEL 4998	EU583422					
	LWZ 20150707-13 (IFP)	**MT319630**	**MT319365**				
	LWZ 20150707-15 (IFP)	**MT319627**					
	LWZ 20150709-2 (IFP)	**MT319626**	**MT319362**		**MT326406**	**MT326358**	**MT326254**
	LWZ 20171015-12 (IFP)[#*]	**MT319625**	**MT319361**	**MT326498**	**MT326407**	**MT326359**	
	Wu 1010-62	KX857799					
	Yuan 5730 (IFP)	**MT319629**	**MT319364**				
	Yuan 5773 (IFP)	**MT319622**	**MT319358**				
	Yuan 5891 (IFP)	**MT319628**	**MT319363**				
‘*X. apacheriensis*’	Wu 0910-58	KX857797					
= *X. jacobaeus*	MA-Fungi 91339	MH430072	MH430071				
	MA-Fungi 91340	MH430073					
	MA-Fungi 91338	MH430074					
= *X. reticulatus*	GC 1512-1	KX857808	KX857813				
	KUC 20160721B-26	MF774798					

	Wu 1109-178	KX857805			
= *X. rhizomorphus*	Dai 12354	KF917544			
	Dai 12367	KF917545			
	Dai 12389	KF917546			
X. nongravis	CHWC 1506-2	KX857800			
	CLZhao 3190	MH114745			
	CLZhao 3196	MH114747			
	Dai 11686 (BJFC)	**MT319614**	**MT319350**		
	Dai 15321	KT989969			
	GC 1412-22	KX857801	KX857818		
	LWZ 20150731-19 (IFP)	**MT319613**	**MT319349**		**MT326394**
	LWZ 20160318-5 (IFP)	**MT319612**	**MT319348**		
	Spirin 5763	MH324469			
X. nothofagi	ICMP 13839	AF145582	MH260064		
	ICMP 13842	AF145583			
	PDD 91630	GQ411524			
X. ovisporus	CLZhao 178	MG231651			
	CLZhao 240	MG231653			
	CLZhao 316	MH114922			
	CLZhao 321	MH114923			
	CLZhao 759	MG231668			
	CLZhao 1050	MG231674			
	CLZhao 1508	MG231691			
	CLZhao 4028	MH114774			

CLZhao 4739	MH114779					
CLZhao 4757	MH114786					
Cui 12060 (BJFC)	**MT319581**	**MT319319**	**MT326549**			**MT326297**
Cui 12304 (BJFC)	**MT319596**	**MT319332**				**MT326306**
Dai 13058 (BJFC)	**MT319593**					**MT326295**
Dai 13824 (BJFC)	**MT319587**	**MT319325**	**MT326552**			**MT326301**
Dai 14958 (BJFC)	**MT319605**					**MT326299**
Dai 15460 (BJFC)	**MT319582**	**MT319320**	**MT326550**			**MT326300**
Dai 15618 (BJFC)	**MT319606**	**MT319339**				**MT326304**
Dai 15626 (BJFC)	**MT319575**					**MT326296**
Dai 15627 (BJFC)	**MT319576**	**MT319314**				**MT326302**
Dai 15628 (BJFC)	**MT319595**					
Dai 15682 (BJFC)	**MT319588**	**MT319326**	**MT326553**			**MT326307**
Dai 15688 (BJFC)	**MT319571**	**MT319312**				**MT326303**
Dai 15699 (BJFC)	**MT319572**					
Dai 15722 (BJFC)	**MT319594**	**MT319331**				**MT326305**
Dai 15724 (BJFC)	**MT319585**	**MT319323**	**MT326551**			**MT326308**
Dai 15730 (BJFC)	**MT319592**	**MT319329**	**MT326554**			**MT326298**
Dai 16306 (BJFC)	**MT319591**					
GEL 3493	EU583421					
ICMP 13830	AF145584					
ICMP 13835	AF145586	MH260063				
KUC 20130725-29	KJ668513					
LWZ 20140726-7 (IFP)	**MT319604**			**MT326367**		
LWZ 20141122-1	**MT319574**			**MT326404**	**MT326352**	**MT326240**

(IFP)						
LWZ 20150622-6 (IFP)	**MT319598**	**MT319333**				
LWZ 20150705-1 (IFP)	**MT319573**	**MT319313**				
LWZ 20150708-15 (IFP)	**MT319579**	**MT319317**	**MT326486**		**MT326319**	**MT326251**
LWZ 20150710-3 (IFP)	**MT319580**	**MT319318**	**MT326548**			
LWZ 20150710-5 (IFP)	**MT319602**	**MT319338**				
LWZ 20150727-1 (IFP)	**MT319603**	**MT319337**	**MT326492**			
LWZ 20150802-7 (IFP)	**MT319599**	**MT319334**				
LWZ 20150802-19 (IFP)	**MT319577**	**MT319315**				
LWZ 20170814-6 (IFP)	**MT319607**	**MT319340**	**MT326493**	**MT326374**	**MT326351**	**MT326247**
LWZ 20170814-12 (IFP)	**MT319583**	**MT319321**	**MT326487**	**MT326368**	**MT326322**	**MT326246**
LWZ 20170814-18 (IFP)	**MT319584**	**MT319322**		**MT326371**	**MT326323**	**MT326243**
LWZ 20170815-16 (IFP)	**MT319663**	**MT319343**				
LWZ 20170815-31	**MT319666**	**MT319346**				

(IFP)						
LWZ 20170815-33 (IFP)#*	**MT319589**	**MT319327**	**MT326523**	**MT326369**	**MT326325**	**MT326248**
LWZ 20170815-39 (IFP)	**MT319600**	**MT319335**	**MT326490**		**MT326324**	**MT326241**
LWZ 20170815-41 (IFP)	**MT319667**	**MT319345**				
LWZ 20170815-45 (IFP)	**MT319669**	**MT319344**				
LWZ 20170816-13 (IFP)	**MT319586**	**MT319324**	**MT326488**	**MT326370**	**MT326320**	**MT326245**
LWZ 20170817-4 (IFP)	**MT319578**	**MT319316**			**MT326357**	**MT326250**
LWZ 20170818-7 (IFP)	**MT319608**	**MT319341**	**MT326494**	**MT326373**	**MT326326**	**MT326244**
LWZ 20170818-19 (IFP)	**MT319601**	**MT319336**	**MT326491**	**MT326372**	**MT326321**	**MT326249**
LWZ 20171015-9 (IFP)	**MT319597**	**MT319330**				
LWZ 20171015-10 (IFP)	**MT319590**	**MT319328**	**MT326489**	**MT326375**	**MT326327**	**MT326242**
LWZ 20180919-12 (HMAS)	**MT319648**	**MT319342**				
LWZ 20180920-1 (HMAS)	**MT319649**	**MT319289**	**MT326546**			
LWZ 20180920-10	**MT319650**	**MT319286**	**MT326543**			

	(HMAS)						
	LWZ 20180920-22 (HMAS)	**MT319652**	**MT319287**	**MT326544**			
	LWZ 20180921-4 (HMAS)	**MT319653**	**MT319288**	**MT326545**			
'*X. flaviporus*'	GC 1509-71	MF540761					
	GC 1607-3	MF540762					
	Wu 0211-53	MF540763					
X. paradoxus	CBS 316.53	MH857217					
	CBS 317.53	MH857218					
	CBS 318.53	MH857219					
	CBS 319.53	MH857220					
	Dai 14983 (BJFC)*	**MT319519**	**MT319267**	**MT326539**	**MT326416**		
	FCUG 1517	AF145572					
	FCUG 2425	AF145571	AY059067				
	JR 06	MH880219					
	MA-Fungi 70444	MH260070	MH260065				
	MA-Fungi 81294	MH260072					
	Miettinen 7978	FN907912					
X. pseudotropicus	Dai 10758	KF917542					
	Dai 10765	KT989974					
	Dai 10768	KF917543					
	Dai 16167 (BJFC)	**MT319509**	**MT319255**	**MT326536**			**MT326312**
	LWZ 20170817-1 (IFP)*	**MT319507**	**MT319254**	**MT326460**			**MT326255**
X. pruinosus	Spirin 2877	MH332700					

	UC2023108	KP814412						
X. pseudolanatus	FP-150922	MH880220	MH884909					
X. quercinus	H 6013352	KT361632						
	KHL 11076	KT361633	AY586678					
	Kotiranta 27060	MH320792						
	MA-Fungi 91311	MH260073	MH260067					
	Spirin 8565	MH316007						
	Spirin 8840	MH320791						
X. ramicida	Spirin 7664	KT361634						
X. rhododendricola	LWZ 20180512-4 (HMAS)*	**MT319619**	**MT319355**	**MT326497**				
	LWZ 20180513-3 (HMAS)	**MT319620**	**MT319356**					
	LWZ 20180513-9 (HMAS)	**MT319621**	**MT319357**					
X. rimosissimus	DLL 2011-081	KJ140600						
	Ryberg 021031	DQ873627	DQ873628					
	UC2022842	KP814311						
	UC2023109	KP814414						
	UC2023147	KP814193						
	UC2023148	KP814194						
X. serpentiformis	FO 40675	MH880228						
	FO 42688	MH880229	MH884913					
	GEL 3668	MH880227						
	LWZ 20170815-14 (IFP)[#]*	**MT319477**	**MT319214**	**MT326517**	**MT326363**	**MT326362**	**MT326265**	**MT326586**

	LWZ 20170815-17 (IFP)	**MT319664**	**MT319216**					
	LWZ 20170815-18 (IFP)	**MT319478**	**MT319215**	**MT326518**	**MT326366**	**MT326338**	**MT326267**	**MT326588**
	LWZ 20170815-29 (IFP)	**MT319665**	**MT319220**					
	LWZ 20170815-42 (IFP)	**MT319668**	**MT319219**					
	LWZ 20170816-11 (IFP)	**MT319475**	**MT319212**	**MT326515**	**MT326364**	**MT326340**	**MT326266**	**MT326589**
	LWZ 20170816-12 (IFP)	**MT319672**	**MT319217**					
	LWZ 20170816-15 (IFP)	**MT319673**	**MT319218**					
	LWZ 20170818-4 (IFP)	**MT319476**	**MT319213**	**MT326516**	**MT326365**	**MT326339**	**MT326268**	**MT326587**
X. spathulatus	CLZhao 184	MG231628						
	Cui 6834 (BJFC)	**MT319618**						
	Cui 12887	KY290982						
	Cui 12888	KY290983						
	GEL 2690	KY081803						
	KHL 7085	KY081804						
	LWZ 20180804-10 (HMAS)	**MT319646**	**MT319354**	**MT326556**				
	LWZ 20180808-4 (HMAS)#*	**MT319616**	**MT319352**	**MT326495**			**MT326280**	

	LWZ 20180808-5 (HMAS)	**MT319617**	**MT319353**	**MT326496**			**MT326281**
	LWZ 20180906-19 (HMAS)	**MT319615**	**MT319351**				
	MMS 7224	MH880230					
	MSK-F 12931	MH880231	MH884914				
	Wu 1307-42	KX857802					
	Wu 1407-105	KX857804	KX857811				
X. subclavatus	FO 42167	MH880232					
X. subflaviporus	A46	KC414243					
	CLZhao 2160	MH114755					
	CLZhao 2176	MH114758					
	Dai 16413 (BJFC)	**MT319567**	**MT319310**	**MT326547**	**MT326415**		**MT326309**
	Dollinger 698	KY264045					
	Dollinger 772	KY264046					
	FLAS-F-61200	MH399868					
	GC 1609-42	MF540767					
	GEL 3466	MH880233					
	ICMP 13837	AF145587					
	LWZ 20150731-11 (IFP)	**MT319566**	**MT319309**	**MT326484**			
	LWZ 20171014-9 (IFP)*	**MT319569**	**MT319311**	**MT326485**			
	LWZ 20180415-1 (HMAS)	**MT319568**					
	Wu 0809-76	KX857803	KX857815				

	Wu 0606-140	MF540768		
	Yuan 4399 (IFP)	**MT319570**		
X. subserpentiformis	LWZ 20180509-2 (HMAS)*	**MT319481**	**MT319223**	**MT326452**
	LWZ 20180512-16 (HMAS)	**MT319486**	**MT319226**	**MT326453**
	LWZ 20180513-28 (HMAS)	**MT319480**	**MT319222**	
	LWZ 20180515-15 (HMAS)	**MT319482**		
	LWZ 20180515-16 (HMAS)	**MT319483**	**MT319224**	
	LWZ 20180515-19 (HMAS)	**MT319484**	**MT319225**	
	LWZ 20180515-22 (HMAS)	**MT319479**	**MT319221**	
X. subtropicus	LWZ 20180509-1 (HMAS)	**MT319526**	**MT319293**	
	LWZ 20180509-3 (HMAS)	**MT319532**	**MT319299**	
	LWZ 20180509-6 (HMAS)*	**MT319537**	**MT319305**	**MT326520**
	LWZ 20180509-16 (HMAS)	**MT319535**	**MT319303**	
	LWZ 20180509-18 (HMAS)	**MT319536**	**MT319302**	**MT326481**

LWZ 20180509-21 (HMAS)	**MT319530**	**MT319297**	**MT326478**			
LWZ 20180509-22 (HMAS)	**MT319531**	**MT319298**	**MT326479**			
LWZ 20180510-3 (HMAS)	**MT319538**	**MT319304**	**MT326482**			**MT326279**
LWZ 20180510-15 (HMAS)	**MT319527**	**MT319294**	**MT326476**			
LWZ 20180510-24 (HMAS)	**MT319541**	**MT319308**				
LWZ 20180512-15 (HMAS)	**MT319539**	**MT319306**	**MT326521**			
LWZ 20180513-12 (HMAS)	**MT319534**	**MT319301**	**MT326480**			
LWZ 20180514-3 (HMAS)	**MT319525**					
LWZ 20180514-8 (HMAS)	**MT319529**	**MT319296**	**MT326477**			
LWZ 20180514-15 (HMAS)	**MT319533**	**MT319300**				
LWZ 20180514-21 (HMAS)	**MT319540**	**MT319307**	**MT326483**			
LWZ 20180515-12 (HMAS)	**MT319528**	**MT319295**				
NZFS 4546	MH409968					
P. CH-4	KF562013					

	UC2022947	KP814552			
	Wu 1508-2	KX857806	KX857812		
	Wu 9806-105	KX857807	KX857809		
= *X. laurentianus*	DLL 2009-049	JQ673187	KY962866	KY967075	
	HHB 719	KY962845	KY962865	KY967076	
= *X. novozelandicus*	ICMP 13829	AF145577	KY962850	KY967071	KY967067
	ICMP 13833	AF145580	KY962853	KY967073	KY967068
	ICMP 13838	AF145578	KY962851	KY967069	
	ICMP 13840	AF145576	KY962849	KY967070	KY967066
	ICMP 13841	AF145579	KY962852	KY967072	KY967065
	PDD 91616	GQ411525			
= *X. patagonicus*	ICMP 13832	AF145581	KY962848	KY967074	KY967058
	MA-Fungi 90702	KY962836	KY962854		KY967062
	MA-Fungi 90704	KY962840			KY967060
	MA-Fungi 90705	KY962835			KY967063
	MA-Fungi 90706	KY962838	KY962856		KY967064
	MA-Fungi 90707	KY962837	KY962855		KY967061
	MA-Fungi 90708	KY962839	KY962857		KY967059
= *X. raduloides*	Dai 12631	KT203307			
	JR 02	MH880221			
	JR 03	MH880222			
	JR 09	MH880223			
	JR 10	MH880224			
	JR 26	MH880225	MH884910		
	LR 18813	MH880226	MH884911		
	MA-Fungi 22499	KY962822	KY962861		

	MA-Fungi 22513	KY962823	KY962862				
	MA-Fungi 35643	KY962831	KY962858				KY967054
	MA-Fungi 75130	KY962824	KY962863		KY967079		KY967057
	MA-Fungi 75272	KY962829	KY962859		KY967077		KY967053
	MA-Fungi 75310	KY962825	KY962864		KY967080		KY967055
	MA-Fungi 90709	KY962844	KY962860		KY967078		KY967056
X. ussuriensis	KUN 1989	MH324468					
X. verecundus	KHL 12261	DQ873642					
X. victoriensis	LWZ 20180510-29 (HMAS)	**MT319487**	**MT319228**				
	LWZ 20180512-11 (HMAS)	**MT319485**	**MT319227**				
X. yarraensis	LWZ 20180509-7 (HMAS)	**MT319640**	**MT319371**				
	LWZ 20180510-4 (HMAS)*	**MT319635**	**MT319370**	**MT326500**			**MT326282**
	LWZ 20180510-5 (HMAS)	**MT319639**	**MT319378**	**MT326505**			
	LWZ 20180510-16 (HMAS)	**MT319637**	**MT319373**				
	LWZ 20180510-19 (HMAS)	**MT319638**	**MT319377**				
	LWZ 20180510-25 (HMAS)	**MT319636**	**MT319369**				
	LWZ 20180512-19 (HMAS)	**MT319645**					

	LWZ 20180512-21 (HMAS)	**MT319641**	**MT319372**	**MT326501**
	LWZ 20180512-22 (HMAS)	**MT319642**	**MT319374**	**MT326502**
	LWZ 20180512-23 (HMAS)	**MT319643**	**MT319375**	**MT326503**
	LWZ 20180512-29 (HMAS)	**MT319644**	**MT319376**	**MT326504**
X. yunnanensis	Cui 8129 (BJFC)	**MT319506**	**MT319251**	
	LWZ 20180920-12a (HMAS)*	**MT319651**	**MT319252**	**MT326534**
	LWZ 20180922-47 (HMAS)	**MT319660**	**MT319253**	**MT326535**
Xylodon sp.	Dai 12590 (BJFC)	**MT319508**	**MT319256**	
Xylodon sp.	H Berglund 1117	DQ873633	DQ873634	
Xylodon sp.	LWZ 20171015-26 (IFP)*	**MT319515**	**MT319260**	**MT326537**
Xylodon sp.	LWZ 20180517-34 (HMAS)	**MT319565**	**MT319285**	
Xylodon sp.	LWZ 20180518-8 (HMAS)	**MT319510**	**MT319257**	
Xylodon sp.	LWZ 20180904-28 (HMAS)	**MT319674**	**MT319366**	
Xylodon sp.	LWZ 20180922-19 (HMAS)*	**MT319657**	**MT319379**	**MT326557**
Xylodon sp.	LWZ 20180922-26	**MT319658**	**MT319380**	**MT326558**

		(HMAS)*			
	'*X. raduloides*'	FCUG 2433	AF145570	AJ406466	
/Incertae sedis	*Atheloderma mirabile*	TAA 169235	DQ873592	DQ873592	
	Basidioradulum radula	AFTOL-ID 451	DQ234537	AY700184	
	Blasiphalia pseudogrisella	Lutzoni 930728-3	U66437	U66437	
	Contumyces rosella	Redhead 7501	U66452	U66452	
	Cotylidia sp.	AFTOL-ID 700	AY854079	AY629317	
	Fibricium rude	CBS 339.66	MH858815	MH870454	
	Globulicium hiemale	Hjm 19007	DQ873595	DQ873595	
	Hastodontia halonata	HHB-17058 (CFMR)	MK575207	MK598738	
	H. hastata	GEL 2143	DQ340323		
		GEL 3124	DQ340311		
		KHL 14646	MH638232	MH638232	
	Leifia brevispora	LWZ 20170820-46	MK343469	**MT319400**	
	Leifia sp.	LWZ 20171015-36 (IFP)	MK343471	**MT319402**	**MT326522**
	Leifia sp.	LWZ 20171015-38 (IFP)	MK343472	**MT319403**	**MT326569**
	Loreleia marchantiae	Lutzoni 930826-1	U66432	U66432	
	Odonticium romellii	1514b	DQ873639	DQ873639	
	Peniophorella	KHL 13164	DQ873597	DQ873597	

	praetermissa				
	Repetobasidium conicum	KHL 12338	DQ873647	DQ873647	
	Rickenella fibula	AFTOL-ID 486	DQ241782	AY700195	
	R. mellea	Lamoure 74	U66438	U66438	
	Sidera lunata	JS 15063	DQ873593	DQ873593	
	Skvortzovia furfuraceum	KHL 11738	DQ873648	DQ873648	
	S. furfurella	KHL 10180	DQ873649	DQ873649	
	S. georgica	KHL 12019	DQ873645	DQ873645	
	S. pinicola	KHL 12224	DQ873637	DQ873637	
	Sphaerobasidium minutum	KHL 11714	DQ873652	DQ873653	
	Sphagnomphalia brevibasidiata	Lutzoni 930826-1	U66441	U66441	
	Trichaptum abietinum	NH 12842	AF347104	AF347104	
	Tubulicrinis globisporus	KHL 12133	DQ873655	DQ873655	
	T. hirtellus	KHL 11717	DQ873657	DQ873657	
	T. inornatus	KHL 11763	DQ873659	DQ873659	
Polyporales /Incertae sedis	*Donkioporiella mellea*	LWZ 20140622-12	KX258957	KX258955	
/*Phanerochaetaceae*	*Ceriporia* sp.	MEL 2400115	**MT332133**	**MT319137**	**MT326417**
	Phanerochaete sp.	MEL 2379809	**MT332134**	**MT319138**	**MT326418**
Thelephorales/*Thele*	*Lenzitopsis daii*	Yuan 2959	JN169799	JN169795	

phoraceae

‘Voucher’[#] means this specimen was used in estimating the divergence times of families within *Hymenochaetales*

‘Voucher’* means this specimen was used in exploring the evolution of hymenophore morphological characters within *Hymenochaetales*.

‘species name’ means this species name is misapplied for the sequence and the accepted species name for this sequence is the name in its previous line of the table.

= species name means this species name is a synonym of the name in its previous line of the table.

[generic name] means this generic name is uncertain.

Newly generated sequences are in bold.
