# Supplementary material for: Towards a Natural Classification of Hyphodontia Sensu Lato and the Trait Evolution of Basidiocarps within Hymenochaetales (Basidiomycota)
Source: J Fungi (Basel). 2021 Jun 12;7(6):478. doi: 10.3390/jof7060478 (PMC8231612; doi:10.3390/jof7060478)
Supplement: Supplementary file 1 [file jof-07-00478-s001.zip › Table S2.pdf]

Table S2 Information of taxa used in molecular clock analysis besides those indicated in Table S1

Phylum/class/order/family	Species	Voucher	ITS	LSU	*tef1α*	*rpb1*	*rpb2*
Ascomycota/*Sordariomycetes*/*Sordariales*/*Sordariaceae*	*Neurospora crassa*	OR74A	HQ271348	AF286411	XM959775		AF107789
Basidiomycota/*Agaricomycetes*/*Agaricales*/*Agaricaceae*	*Lepiota cristata*	ZRL20151133	LT716026	KY418841	KY419048	KY418963	KY418992
-/-/-/*Strophariaceae*	*Gymnopilus picreus*	ZRL2015011	LT716066	KY418882	KY419077	KY418980	KY419027
-/-/*Amylocorticiales*/*Amylocorticiaceae*	*Anomoloma myceliosum*	MJL 4413	GU187500	GU187559	GU187677	GU187441	GU187766
	Amylocorticium cebennense	HHB 2808	GU187505	GU187561	GU187675	GU187439	GU187770
-/-/*Atheliales*/*Atheliaceae*	*Athelia arachnoidea*	CBS 418.72	GU187504	GU187557	GU187672	GU187436	GU187769
	Leptosporomyces raunkiaeri	HHB 7628	GU187528	GU187588	GU187719	GU187471	GU187791
-/-/*Auriculariales*/*Auriculariaceae*	*Auricularia heimuer*	Xiaoheimao	LT716074	KY418890	KY419083	KY418982	KY419035
-/-/*Boletales*/*Gomphidiaceae*	*Gomphidius roseus*	MB 95-038	DQ534570	DQ534669	GU187702	GU187459	GU187818
-/-/-/*Suillaceae*	*Suillus pictus*	AFTOL 717	AY854069	AY684154	AY883429	AY858965	AY786066
-/-/*Cantharellales*/*Clavulinaceae*	*Multiclavula mucida*	AFTOL 1130	DQ521417	AY885163			
-/-/-/*Hydnaceae*	*Hydnum repandum*	BB 07.341		KF294643	JX192980		KF294720
-/-/*Corticiales*/*Corticiaceae*	*Corticium roseum*	MG43	GU590877	AY463401			
-/-/-/*Vuilleminiaceae*	*Australovuilleminia coccinea*	MG75	HM046875	HM046931			
-/-/*Geastrales*/*Geastraceae*	*Pyrenogaster pityophilus*	OSC 59743		DQ218519	DQ219232		DQ219057
	Geastrum recolligens	OSC 41996		DQ218486	DQ219230		DQ219052
-/-/*Gloeophyllales*/*Gloeophyllaceae*	*Gloeophyllum sepiarium*	Wilcox-3BB	HM536091	HM536061	HM536110		HM536109
	Gloeophyllum striatum	ARIZAN 027866	HM536092	HM536063	HM536111		HM640259
-/-/*Gomphales*/*Gomphaceae*	*Ramaria rubella*	AFTOL 724	AY854078	AY645057	AY883435		AY786064
	Gautieria otthii	AFTOL 466	AY883434	AF393058	AY883434		AY218486
-/-/*Hymenochaetales*/*Hymenochaetaceae*	*Fomitiporia mediterranea*	AFTOL 688	AY854080	AY684157	AY885149	AY864870	AY803748

	F. hartigii	MUCL 53551	JX093789	JX093833	JX093746		JX093877
	Porodaedalea chinensis	Cui 10252	KX673606	MH152358	MG585301		MH101479
-/-/-/*Oxyporaceae*	*Rigidoporus corticola*	ZRL20151459	LT716075	KY418899	KY419087		KY419038
-/-/*Hysterangiales*/*Hysterangiaceae*	*Aroramyces gelatinosporus*	H4010		DQ218524	DQ219118		DQ218941
-/-/-/*Mesophelliaceae*	*Chondrogaster pachysporus*	OSC 49298		DQ218538	DQ219136		DQ218958
-/-/*Jaapiales*/*Jaapiaceae*	*Jaapia argillacea*	CBS 252.74	GU187524	GU187581	GU187711	GU187463	GU187788
-/-/*Phallales*/*Phallaceae*	*Dictyophora duplicata*	OSC 38819		DQ218481	DQ219265		DQ219087
	Phallus costatus	MB 02040		DQ218513	DQ219279		DQ219104
-/-/*Polyporales*/*Meripilaceae*	*Grifola frondosa*	AFTOL 701	AY854084	AY629318	AY885153	AY864876	AY786057
-/-/-/*Polyporaceae*	*Trametes versicolor*	ZRL20151477	LT716079	KY418903	KY419091	KY418984	KY419041
-/-/*Russulales*/*Bondarzewiaceae*	*Bondarzewia montana*	AFTOL 452	DQ200923	DQ234539	DQ059044	DQ256049	AY218474
-/-/*Sebacinales*/*Sebacinaceae*	*Craterocolla cerasi*	TUB 020203	KF061265	KF061265			KF061300
	Tremellodendron sp.	PBM 2324	DQ411526		DQ029196		DQ408132
-/-/*Stereopsidales*/*Stereopsidaceae*	*Stereopsis* sp.	OKHL 15544			KC203519		KC203505
	Stereopsis radicans	OLR 45395		KC203496	KC203516		KC203502
-/-/*Thelephorales*/*Bankeraceae*	*Boletopsis leucomelaena*	AFTOL 1527	DQ484064	DQ154112	GU187763	GU187494	GU187820
-/-/-/*Thelephoraceae*	*Thelephora ganbajun*	ZRL20151295	LT716082	KY418908	KY419093	KY418987	KY419043
-/-/*Trechisporales*/*Hydnodontaceae*	*Brevicellicium olivascens*	KHL 8571	HE963792	HE963793			
	Trechispora alnicola	AFTOL 665	DQ411529	AY635768	DQ059052		
-/*Dacrymycetes*/*Dacrymycetales*/*Dacrymycetaceae*	*Calocera cornea*	AFTOL 438	AY789083	AY701526	AY881019		AY536286
	Dacryopinax spathularia	AFTOL 454	AY854070	AY701525	AY881020		AY786054
-/*Tremellomycetes*/*Tremellales*/*Tremellaceae*/	*Cryptococcus humicola*	AFTOL 1552	DQ645516	DQ645514	DQ645519		DQ645517
-/*Ustilaginomycetes*/*Ustilaginales*/*Ustilaginaceae*	*Ustilago maydis*	AFTOL 505	AY854090	AF453938	AY885160		AY485636
